# Supplementary material for: Saturation Mutagenesis of the Transmembrane Region of HokC in Escherichia coli Reveals Its High Tolerance to Mutations
Source: Int J Mol Sci. 2021 Sep 26;22(19):10359. doi: 10.3390/ijms221910359 (PMC8509063; doi:10.3390/ijms221910359)
Supplement: Supplementary file 1 [file ijms-22-10359-s001.zip › ijms-1387147-supplementary.pdf]

SUPPORTING INFORMATION FOR

Saturation mutagenesis of the transmembrane region of HokC in *Escherichia coli* reveals its high tolerance to mutations

Maria Teresa Lara Ortiz, Victor Martinell Garcia, Gabriel Del Rio

I. Supplemental Tables

Table S1. Colonies counted under different IPTG concentrations.

IPTG (mM)	Exp 1	Control	Exp 2	Control	Exp 3	Control
0.01	86	237	105	105	107	162
0.05	---	253	---	241	---	151
0.1	---	124	---	230	---	334
0.2	---	192	---	120	---	268
0.4	---	213	---	211	---	218
0.6	---	227	---	245	---	231
0.8	---	263	---	210	---	202

Exp: Experiment; cells were grown in the presence of Kan 10 microg/mL and the indicated concentration of IPTG. Control: cells were grown in the absence of IPTG, only in the presence of Kan 10 microg/mL.

Table S2. Observed phenotypes per mutated region of HokC.

**Region**	**Total isolated colonies**	**Wild-type phenotype**	**Mutant phenotype**
**I (2-6)**	**960**	**531**	**429**
**II (7-9)**	**1015**	**807**	**208**
**III (10-12)**	**931**	**346**	**585**
**IV (13-15)**	**914**	**320**	**594**
**V* (16-18)**	**945**	**246**	**699**
**VI (19-21)**	**877**	**657**	**220**
**VII (22-24)**	**777**	**299**	**478**

The mutated regions (I toVII; in parenthesis the positions included in each region is reported), with their corresponding number of isolated colonies identified with

mutant or wild-type phenotype are presented. Region V was reported previously by our group and is presented here for comparison.

Table S3. Single-residue mutations found in every mutant of HokC with wild-type (A) and mutant (B) phenotypes.

A)

Mutation	Occurrences
V9*	302
K2T	11672
Y47X	15
K2S	10488
K2R	40027
K2Q	2167
Y47T	6732
K2P	826
Y47S	102
K2N	8232
Y47R	5184
K2M	965
K2L	111
Y47N	37
K2I	121
K2H	395
K2G	327
K2F	46
K2E	3448
K2D	688
Y47H	59
K2C	118
Y47F	65
K2A	2241
Y47E	1

Y47D	32
Y47C	146
K2*	843
Y47*	58
V20Y	1
V20X	3871
V20W	9452
V20T	12239
V20S	114
V20R	508
V20Q	546
V20P	5867
V20N	251
V20M	2389
V20L	3140
V20K	277
V20I	287
V20H	15
V20G	14772
V20F	291
V20E	1713
V20D	1290
V20C	11
V20A	9061
V19Y	3132
V19X	3891
V19W	15
V19T	2374
V19S	6610

V19R	1896
V19Q	9543
V19P	5428
V19N	2653
V19M	929
V19L	11277
V19K	10
V19I	456
V20*	18
V19H	1205
V19G	7525
V19F	2628
V19E	1089
V19D	420
V19C	326
V19A	297
T45X	1
T45V	1
T45S	136
T45R	6750
T45Q	2
T45P	330
T45M	665
T45L	1
T45K	70
T45I	1
T45G	5177
V19*	6827
T45E	1

T45D	2
T45A	529
E50Y	1
A10Y	1599
E50X	1
A10X	206
E50W	1
E50V	5220
A10W	958
A10V	5845
A10T	4005
E50S	6729
A10S	5778
A10R	2876
E50Q	8
A10Q	18
A10P	53509
A10N	623
A10M	36
A10L	1722
E50K	132
A10K	1255
A10I	1039
E50H	1
A10H	621
E50G	74
A10G	23322
A10F	771
A10E	275

E50D	30
A10D	1705
T45*	2
A10C	1697
E50A	20
E50*	39
A10*	49
R26X	2
I8Y	105
I8X	338
R26V	1
I8W	2428
I8V	12185
R26T	11
R26S	113
I8T	2865
I8S	3836
R26Q	2
I8R	8471
I8Q	355
I8P	7347
R26N	4
I8N	756
I8M	10274
R26K	5104
I8L	23759
E48X	1
I8K	1704
R26I	119

E48V	23
R26G	117
I8H	1161
I8G	11455
R26E	6784
E48S	1
I8F	49002
A22Y	31
I8E	815
A22X	74
E48Q	12
R26C	1
A22W	570
I8D	381
E48P	3
I8C	5923
A22V	1280
R26A	2
E48N	6730
A22T	3327
I8A	5448
A22S	2062
A22R	8937
E48K	99
A22Q	255
A22P	3352
E48I	5186
A22N	708
E48G	57

A22M	134
A22L	883
E48D	150
A22K	181
A22I	78
A22H	490
E48A	49
A22G	727
A22F	103
A22E	1959
A22D	526
R26*	36
I8*	100
A21Y	517
A21X	361
A21W	13
A21V	1920
C30Y	34
A21T	11407
C30X	4
A21S	3034
C30W	110
A21R	10148
C30V	6760
A21Q	7
A21P	11992
E48*	30
C30S	67
C30R	64

A21N	330
A21M	744
A21L	1247
A21K	26
A21I	415
A22*	9
C30M	1
A21H	379
C30L	7
A21G	14051
A21F	200
A21E	329
A21D	99
A21C	6
C30G	126
C30F	126
A6Y	24
A6X	325
A6W	840
A6V	3659
A6T	453
A6S	807
A6R	8360
V13Y	2121
A6Q	145
A6P	2946
V13X	3870
V13W	685
A6N	7

A6M	42
V13T	2000
A6L	554
V13S	9585
A6K	53
V13R	159
V13Q	23
A6I	5
V13P	4283
A6H	118
A6G	52275
V13N	225
A6F	17
A6E	4551
V13M	81
V13L	4313
A6D	2429
A6C	44
V13K	55
A21*	31
V13I	1961
V13H	4806
V13G	3677
C30*	5088
V13F	14707
V13E	737
V13D	2992
V13C	481
V13A	12157

A6*	95
I34X	5
V13*	109
I34V	81
I34T	68
I34S	7057
I34R	2
I34P	5084
I34N	155
I34M	19
I34L	150
Q3Y	34
I34K	4
Q3X	374
Q3W	150
Q3V	143
I34H	10
Q3T	1746
I34F	33
Q3S	5993
Q3R	33876
I34C	1
Q3P	8363
Q3N	1051
Q3M	147
Q3L	2424
Q3K	7971
Q3I	42
Q3H	11346

Q3G	822
Q3F	5
Q3E	4026
Q3D	471
Q3C	236
Q3A	1989
Q3*	310
L11Y	190
L11X	514
L11W	7195
L11V	14823
L11T	2094
L11S	3732
V43X	1
L11R	25224
L11Q	1545
L11P	4684
L11N	194
L11M	5666
L11K	1613
L11I	869
L11H	2080
V43M	1
L11G	14067
V43L	6
L11F	2835
L11E	2683
L11D	4146
V43I	48

L11C	479
V43H	1
V43G	96
L11A	4395
V43F	11947
V43E	2
V43D	31
V43C	4
V43A	30
L11*	7137
T25X	27
T25V	1
T25S	74
T25R	6808
T25Q	8
T25P	299
T25M	45
T25L	1
T25K	86
T25G	2
T25E	4978
V41X	2
T25A	463
V41S	1
V41L	6738
V41I	30
V41G	56
V41F	68
V41E	1

V41D	23
V41C	5156
V41A	25
T25*	4
S49Y	58
S49X	1
S49T	119
S49R	5187
S49P	6808
S49N	2
S49L	1
S49H	1
S49G	1
S49F	60
S49C	10
S49A	262
I16Y	265
I16X	3812
I16W	2
I16V	1269
I16T	52882
I16S	15550
I16R	372
I16Q	8
I16P	7345
I16N	1802
I16M	164
I16L	9567
I16K	73

I16H	5277
I16G	51
I16F	4518
I16E	3
I16D	82
I16C	356
I16A	811
I16*	6
I14Y	485
I14X	3826
I14W	607
I14V	5437
I14T	4596
I14S	12235
I14R	3575
I14Q	14
I14P	1110
I14N	2301
I14M	3211
I14L	20774
I14K	6441
I14H	171
I14G	1412
I14F	31394
I14E	37
I14D	71
I14C	1418
I14A	667
I14*	341

I12Y	1079
I12X	482
I12W	2643
I12V	9956
I12T	12825
I12S	24472
I12R	10151
I12Q	838
I12P	3632
I12N	757
I12M	8093
I12L	18933
I12K	364
L29X	2
I12H	216
L29V	18
I12G	14335
I12F	20626
L29T	1
L29S	6763
I12E	486
L29R	207
I12D	88
I12C	5590
L29P	79
I12A	10191
R35X	3
L29I	68
L29H	90

L29G	2
R35S	1
L29F	30
R35Q	168
R35P	26
R35N	5084
L29A	3
R35L	69
R35H	1
R35G	19
R35E	6752
I12*	2175
R35D	3
V24Y	242
V24X	42
R35A	1
V24W	16
V24T	419
V24S	3150
V24R	3929
V24Q	649
V24P	3457
V24N	5841
V24M	19
V24L	1763
V24K	8
V24I	147
V24H	1108
V24G	939

V24F	431
V24E	112
V24D	285
V24C	138
V24A	319
R35*	62
V24*	7586
T39X	1
T39W	1
T39V	3
T39S	80
T39R	6773
T39Q	4
T39P	175
T39M	47
T39L	1
T39K	38
L23Y	1
L23X	10
L23W	6773
L23V	2945
T39G	5116
L23T	699
T39E	5
L23S	36
L23R	880
L23Q	172
H33Y	57
L23P	6430

T39A	276
H33X	5
L23M	932
H33T	6751
L23K	1580
H33S	5
M7Y	493
H33R	278
H33Q	95
L23I	939
M7X	159
M7W	8711
H33P	342
L23H	210
A18Y	5
A18X	3798
M7V	17044
L23G	5491
H33N	77
L23F	98
M7T	2797
L23E	856
A18V	57
H33L	107
M7S	5151
L23D	3
M7R	17064
A18T	318
H33K	2

L23C	50
M7Q	1096
A18S	74
M7P	4495
A18R	4826
L23A	168
H33I	1
A18P	6984
M7N	1235
M7L	27167
A18N	1
M7K	1912
H33D	12
A18L	2
M7I	5711
M7H	363
M7G	11714
M7F	2268
A18H	4
M7E	482
A18G	104
M7D	2714
M7C	2002
A18E	4
A18D	109
M7A	5540
L23*	37
M7*	7592
T36Y	1

T36X	2
T36W	5087
T36S	157
T36R	2
T36P	360
T36N	82
T36M	2
T36L	6759
T36K	1
T36I	40
T36H	1
T36G	1
T36D	3
T36C	1
T36A	376
T36*	2
E40X	1
E40W	1
E40V	49
E40S	18
E40R	6733
E40Q	7
E40K	57
E40G	5265
E40D	82
E40A	59
E40*	43
M1T	2061
M1S	29

M1R	65816
M1K	9962
M1I	11826
D28Y	89
D28V	824
D28T	6772
D28S	8
D28R	1
D28Q	1
D28P	5004
D28N	47
D28K	3
D28I	4
D28H	21
D28G	1240
A46X	2
D28F	5
D28E	162
F44Y	59
F44X	65
A46V	56
D28C	4
A46T	633
F44V	63
D28A	1447
A46S	53
F44S	6796
A46P	18
A46N	2

F44P	1
A46L	11915
F44N	2
A46K	1
F44L	164
F44I	41
A46G	38
F44H	5171
A46F	1
F44G	1
A46E	1
A46D	110
F44C	101
H4Y	1022
H4X	629
H4W	41
H4V	306
H4T	896
H4S	682
H4R	4119
F44*	1
H4Q	46515
H4P	1337
H4N	14709
H4M	270
H4L	4525
H4K	14108
H4I	7084
H4G	214

H4F	46
H4E	1897
H4D	1753
H4C	62
H4A	55
H4*	2287
Q38X	7
Q38V	1
Q38T	1
Q38S	4
Q38R	6838
Q38P	43
Q38N	7
Q38L	31
Q38K	124
Q38H	46
Q38G	3
Q38E	52
Q38D	5100
Q38C	1
A42X	3
A42W	1
C15Y	7296
A42V	323
C15X	3821
C15W	1448
A42T	124
C15V	9711
A42S	52

A42R	3
C15T	626
C15S	3397
A42P	4
C15R	4939
C15Q	118
C15P	2806
A42L	6732
C15N	142
C15L	2802
Q38*	75
C15K	551
A42G	43
C15I	106
C15H	6470
A42E	1
C15G	13446
A42D	59
C15F	16295
A42C	5161
C15E	4607
E31Y	2
C15D	401
E31X	5
E31W	3
E31V	67
C15A	853
E31S	14
E31R	6744

E31Q	14
E31N	1
E31K	66
E31G	5211
E31D	141
T17Y	1
T17X	3817
E31A	114
T17S	3433
C15*	3496
T17R	4813
T17P	51372
T17N	39
T17L	3
T17K	1
T17I	43
T17H	8
T17G	2
T17D	8
T17C	6
T17A	15882
E31*	70
K5Y	13
K5X	438
K5W	180
K5V	240
K5T	2657
K5S	496
K5R	103567

K5Q	1446
K5P	843
K5N	246
K5M	1214
K5L	272
K5I	13
K5H	94
K5G	10270
K5F	16
K5E	6297
K5D	42
V32Y	1
K5C	15
V32X	1
K5A	793
V32S	5061
V32N	1
V32M	1
V32L	37
V32I	73
K27X	88
V32G	124
V32F	6878
V32E	2
V32D	44
K27T	106
V32C	3
K27S	2
K27R	5101

V32A	35
K27Q	112
K5*	397
G37X	5
K27P	3
G37W	2
K27N	183
G37V	78
V9Y	352
K27M	1
V9X	190
G37T	1
V9W	30
G37S	66
G37R	15
K27I	28
V9T	987
G37P	5094
K27H	2
V9S	10631
K27G	1
V9R	5556
G37N	1
V9Q	459
K27E	74
V9P	7616
K27D	2
G37L	2
V9N	655

V9M	319
V9L	4822
V9K	459
G37F	1
V9I	966
G37D	848
V9H	870
V9G	30343
G37C	56
V32*	2
V9F	3555
V9E	2254
G37A	6774
V9D	2385
V9C	562
V9A	44989
K27*	40
K2Y	40
K2X	213
K2W	39
K2V	892

B)

**Mutation**	**Occurrences**
V9*	755
K2T	8207
Y47X	12
K2S	11657
K2R	24565

K2Q	2131
Y47T	9969
K2P	299
Y47S	111
K2N	5575
Y47R	6898
K2M	1199
K2L	318
Y47N	38
K2I	269
K2H	1122
Y47L	3
K2G	1089
K2F	1
K2E	2740
K2D	343
Y47H	33
K2C	110
Y47F	78
K2A	2997
Y47E	1
Y47D	49
Y47C	137
Y47A	1
K2*	935
Y47*	60
V20Y	1
V20X	10501
V20W	10082

V20T	1952
V20S	50
V20R	736
V20Q	18
V20P	4959
V20N	10
V20M	133
V20L	2199
V20I	14
V20H	8
V20G	9711
V20F	13
V20E	13320
V20D	183
V20C	34
V20A	7189
V19Y	400
V19X	8572
V19W	6
V19T	694
V19S	7130
V19R	45
V19Q	27
V19P	19209
V19N	6
V19M	3
V19L	391
V19K	4
V19I	1924

V20*	1
V19H	25
V19G	11908
V19F	555
V19E	90
V19D	11
V19C	34
V19A	5067
T45X	1
T45W	1
T45S	159
T45R	9997
T45Q	5
T45P	405
T45M	524
T45L	2
T45K	105
T45G	6893
V19*	10404
T45E	3
T45A	602
A10Y	655
E50X	2
A10X	3538
E50V	6917
A10W	1145
A10V	2240
A10T	4179
E50S	9965

A10S	5900
E50R	1
A10R	5950
E50Q	15
A10Q	305
E50P	1
A10P	46475
E50N	1
A10N	833
A10M	12
A10L	1976
E50K	88
A10K	1576
A10I	36
A10H	2290
E50G	86
A10G	21474
A10F	499
A10E	3506
E50D	49
A10D	2791
A10C	1624
E50A	33
E50*	31
A10*	375
R26X	1
I8Y	49
I8X	3497
R26V	3

I8W	417
I8V	8042
R26T	19
R26S	121
I8T	3087
I8S	2697
R26Q	2
I8R	4344
I8Q	97
I8P	5122
R26N	2
R26M	1
I8N	1151
I8M	9020
R26K	6748
E48Y	1
I8L	19153
I8K	1464
R26I	139
E48V	18
R26G	147
I8H	1688
I8G	7556
R26E	10012
I8F	25155
E48R	2
A22Y	1
R26D	1
A22X	4532

I8E	1495
E48Q	7
R26C	1
A22W	1305
I8D	614
E48P	1
I8C	6178
A22V	1234
R26A	2
E48N	9968
I8A	4502
A22T	2864
A22S	1753
A22R	12449
E48K	88
A22Q	116
E48I	6898
A22P	866
E48G	68
A22N	5
A22M	1526
A22L	429
E48D	181
A22K	126
A22I	52
A22H	2769
E48A	75
A22G	2201
A22F	454

A22E	247
A22D	1
R26*	39
I8*	142
A21Y	63
A21X	12960
A21W	500
A21V	6036
C30Y	51
A21T	2035
C30X	18
A21S	2701
C30W	133
C30V	10003
A21R	10122
A21Q	11
A21P	4218
E48*	39
C30S	78
C30R	76
A21N	6
A21M	5
A21L	4509
A21K	37
C30N	1
A22*	2606
A21I	29
A21H	3747
C30L	8

A21G	7469
A21F	380
A21E	1438
A21D	1478
C30G	167
A21C	2
C30F	101
C30E	1
A6Y	15
C30D	1
A6X	1221
A6W	526
A6V	3204
C30A	1
A6T	448
A6S	747
A6R	11288
A6Q	518
V13Y	628
A6P	2089
V13X	1183
V13W	419
A6N	7
A6M	290
V13T	1263
A6L	1034
V13S	13241
A6K	127
V13R	778

V13Q	114
A6I	2
V13P	4787
A6H	145
A6G	30582
V13N	1325
A6F	4
A6E	3888
V13M	1555
V13L	6108
A6D	3618
V13K	1007
A21*	3864
A6C	31
V13I	1984
V13H	6520
V13G	18395
C30*	6765
V13F	9562
V13E	3969
V13D	4914
V13C	1738
V13A	13043
A6*	229
I34X	1
V13*	92
I34V	88
I34T	80
I34S	10335

I34R	3
I34P	6760
I34N	194
I34M	18
I34L	161
Q3Y	29
I34K	3
Q3X	978
Q3W	923
Q3V	124
I34H	6
I34G	1
Q3T	1191
I34F	32
Q3S	9186
Q3R	21695
I34C	1
Q3P	5650
Q3N	507
Q3M	274
Q3L	2172
Q3K	6402
Q3I	25
Q3H	7237
Q3G	1059
Q3F	4
Q3E	3172
Q3D	338
Q3C	312

Q3A	3048
Q3*	451
L11Y	245
L11X	2389
L11W	5545
L11V	9196
L11T	2693
V43Y	1
L11S	1761
L11R	29759
V43W	1
L11Q	5527
L11P	9772
V43T	1
L11N	74
V43S	1
L11M	2533
L11K	2786
L11I	229
V43N	2
L11H	1517
L11G	16308
V43L	11
L11F	1107
V43K	1
L11E	3236
L11D	5811
V43I	43
L11C	276

V43G	100
L11A	2636
V43F	16905
V43E	2
V43D	33
V43C	6
V43A	26
L11*	13613
T25X	1211
T25W	1
T25V	2
T25S	121
T25R	10043
T25Q	9
T25P	321
T25M	48
T25L	1
T25K	94
T25G	1
T25E	6640
V41Y	4
T25D	1
V41X	4
V41W	2
T25A	359
V41S	2
V41M	1
V41L	9980
V41I	25

V41G	76
V41F	70
V41E	1
V41D	27
V41C	6855
V41A	25
T25*	2
S49Y	85
S49X	1
S49T	72
S49R	6898
S49P	10028
S49N	2
S49F	44
S49C	14
S49A	195
I16Y	164
I16X	101
I16W	4
I16V	1314
I16T	26982
I16S	15520
I16R	333
I16Q	8
I16P	3658
I16N	1604
I16M	213
I16L	6224
I16K	75

I16H	6675
I16G	47
I16F	3615
I16E	4
I16D	65
S49*	8
I16C	304
I16A	541
I16*	14
I14Y	409
I14X	251
I14W	1830
I14V	7014
I14T	7920
I14S	16992
I14R	10481
I14Q	490
I14P	5830
I14N	3376
I14M	3975
I14L	13087
I14K	1677
I14H	368
I14G	5702
I14F	16671
I14E	1848
I14D	2223
I14C	1787
I14A	3920

I14*	153
I12Y	1712
I12X	2135
I12W	2487
I12V	9998
I12T	10486
I12S	21608
I12R	16617
I12Q	1767
I12P	10841
I12N	2596
I12M	3574
I12L	10455
I12K	2126
L29Y	1
L29X	1
I12H	2487
L29V	30
I12G	14911
I12F	10518
L29T	1
L29S	10005
I12E	1921
I12D	1391
L29R	251
I12C	2810
L29Q	4
L29P	72
I12A	6497

L29N	1
R35Y	1
L29M	1
R35X	2
L29I	81
L29H	101
L29F	24
R35Q	139
R35P	16
L29C	2
R35N	6762
L29A	1
R35L	50
R35K	3
R35G	15
R35E	10000
I12*	3079
R35D	4
V24Y	9
V24X	1638
V24W	1903
V24T	2510
V24S	3992
V24R	403
V24Q	2
V24P	625
V24N	6725
V24M	4140
V24L	4003

V24K	308
V24I	916
V24H	135
V24G	546
V24F	5
V24E	475
V24D	186
V24C	2
V24A	3617
R35*	45
V24*	10796
T39W	1
T39S	95
T39R	10012
T39Q	1
T39P	203
T39M	47
T39L	2
T39K	49
L23X	1105
T39I	1
L23W	9991
T39G	6801
L23V	2440
L23T	65
T39E	4
L23S	478
L23R	3832
L23Q	247

H33Y	51
L23P	1303
T39A	285
H33X	1
H33V	1
L23M	3803
H33T	10005
L23K	7
H33S	2
M7Y	331
H33R	356
H33Q	91
M7X	1600
L23I	91
M7W	4613
H33P	321
L23H	63
M7V	9764
L23G	6994
A18X	73
L23F	512
H33N	113
M7T	2389
L23E	3770
A18V	34
M7S	2765
H33L	130
L23D	5
M7R	11193

A18T	306
H33K	4
L23C	53
M7Q	2276
A18S	194
M7P	2332
A18R	6396
L23A	1266
H33I	1
A18Q	1
A18P	10115
M7N	883
M7L	14968
A18N	1
M7K	1000
H33D	17
A18L	5
M7I	5724
M7H	368
M7G	10770
A18I	2
M7F	975
M7E	2361
A18G	152
M7D	5121
M7C	625
A18E	6
A18D	157
M7A	2413

A18C	1
L23*	5552
H33*	1
M7*	12027
T36W	6767
T36S	154
T36R	1
T36P	376
T36N	76
T36M	2
T36L	10004
T36I	27
T36H	3
T36F	1
T36D	2
T36C	1
T36A	476
T36*	3
E40X	2
E40V	35
E40S	14
E40R	9969
E40Q	10
E40N	1
E40M	1
E40L	1
E40K	51
E40G	6990
E40D	62

E40A	66
E40*	47
M1T	1768
M1S	39
M1R	43423
M1K	6743
M1I	10860
D28Y	122
D28X	25
D28V	983
D28T	10006
D28S	10
D28R	1
D28Q	2
D28P	6680
D28N	65
D28L	1
D28K	2
D28I	3
D28H	15
D28G	1572
D28F	8
D28E	195
F44Y	55
A46V	45
F44X	80
D28C	15
A46T	480
F44V	70

D28A	1698
A46S	71
F44S	10021
A46P	12
F44R	1
F44Q	1
A46N	2
A46L	16864
F44N	3
F44L	164
A46G	44
F44I	49
F44H	6885
F44G	1
A46D	107
F44C	118
H4Y	635
H4X	1493
H4W	488
H4V	183
H4T	417
H4S	577
H4R	3714
F44*	4
H4Q	28009
H4P	2020
H4N	9810
H4M	193
H4L	3542

H4K	7321
H4I	10003
H4G	304
H4F	35
H4E	1458
H4D	2503
H4C	184
H4A	90
H4*	3348
Q38X	8
Q38W	1
Q38T	1
Q38S	6
Q38R	10054
Q38P	44
Q38N	5
Q38L	13
Q38K	102
Q38I	1
Q38H	36
Q38E	51
Q38D	6791
Q38A	1
A42X	2
A42W	1
C15Y	10383
A42V	267
C15X	238
A42T	78

C15W	5157
C15V	13566
A42S	42
A42R	6
C15T	780
C15S	6745
A42P	8
C15R	9394
C15Q	478
C15P	5533
A42L	9973
C15N	1267
C15L	1684
Q38*	56
C15K	3366
A42H	1
A42G	46
C15I	86
A42F	1
C15H	649
C15G	18155
A42D	72
C15F	8680
A42C	6868
C15E	2738
E31Y	3
C15D	2236
E31X	18
E31W	4

E31V	45
C15A	2268
E31S	7
E31R	9990
E31Q	8
E31N	1
E31M	1
E31K	70
E31G	6875
E31D	174
T17Y	3
T17X	93
E31C	1
E31A	129
T17V	2
C15*	5173
T17S	3286
T17R	6382
T17P	36194
T17N	43
T17L	2
T17I	47
T17H	12
T17G	5
T17D	8
T17C	7
T17A	12562
E31*	83
K5Y	3

K5X	1634
K5W	263
K5V	409
K5T	2455
K5S	503
K5R	67004
K5Q	1174
K5P	301
K5N	310
K5M	1170
K5L	474
K5I	15
K5H	25
K5G	9493
K5F	2
K5E	4797
K5D	491
V32Y	1
K5C	12
K5A	357
V32T	1
V32S	6734
V32N	1
V32M	3
V32L	48
V32I	87
K27X	1555
V32G	163
V32F	10155

V32E	1
K27V	1
V32D	47
K27T	168
V32C	3
K27S	5
K27R	6813
V32A	36
K27Q	113
K5*	369
K27P	3
G37X	1
G37W	2
K27N	176
G37V	57
V9Y	163
K27M	1
V9X	3619
G37T	1
K27L	1
G37S	60
V9W	24
G37R	14
K27I	38
G37P	6779
V9T	261
K27H	1
V9S	11973
K27G	1

V9R	6370
V9Q	54
K27E	101
V9P	7498
K27D	2
G37L	1
V9N	380
V9M	43
V9L	1888
V9K	398
G37F	1
V9I	319
G37E	3
V9H	657
G37D	730
V9G	22030
G37C	52
V9F	1821
V9E	3590
G37A	10018
V9D	2873
V9C	470
V9A	27269
K27*	34
K2Y	49
K2X	527
K2W	43
K2V	105

* represents a mutation that rendered a stop mutation. X is a mutation that rendered an indel. The mutation is described following the format [wild-type residue][position][mutated residue]; the residues are specified by a one-letter code.

Table S4. Single-residue mutations on the TM region of HokC.

Position	Tolerance	Intolerance	Substitutions in the alignment
M1	R,K,I		A,M
K2	T,S,R,Q,N,E	M	**M**,N,T,P,S,I,K,L,Q,W
Q3	R,P,N,K,H,E	L	Q,R,P,S
H4	R,Q,P,N,L,K,I,E,D	Y	Q,L,N,R,H,G,A
K5	T,R,Q,P,N,M,G,E		K,H,R,Q,N,S
A6	V,S,P,G,E	T	V,**T**,P,S,C,Y,A,I
M7	V,T,R,L,I	W,K	M,G,R,V,I,F,A,L
I8	V,T,S,R,P,N,M,L,G,F,D,C		I,V,L,S
V9	Y,S,I,G,F,D,C,A		V,M,G,W,L,A,F,I
A10	V,T,S,R,P,G,F,D,C		T,S,M,G,A,I,C
L11	W,V,R,Q,M,G,F,A	P	L,T,V,A,I
I12	W,V,T,R,P,N,M,L,K,G,F	S,D,C	G,F,I,V,L
V13	P,L,G,F,D,A	I	A,M,**I**,V,T
I14	Y,V,T,R,N,M,L,F,A	S,P	M,F,I,V,L,**S**
C15	Y,W,S,R,P,G,F,E,A		C
I16	V,T,S,N,M,L,F		C,F,I,A,M,V,L
T17	S,P,I,A		T
A18	V,T,S,P,D	G	I,A,M,V,L
V19	Y,Q,P,I,F,A	L,G	I,**L**,V,P
V20	P,M,L,G,A		F,I,L,M,G,C,V,T,S
A21	W,V,S,P,G,E	T	F,I,A,**T**,L
A22	V,R,Q,P,N,G	T,S	V,**T**,L,S,A,I
L23	V,T,Q,P,M,I		Y,L,W,F,C

V24	Y,S,R,L,I,G,A	V,L,I,M

Amino acid residue names are represented using the one-letter codes. Every amino acid and its position are indicated in the Position column. Mutations rendering “Wild-type Phenotype” or “Mutant Phenotype” are shown in columns labeled “Tolerance” and “Intolerance”, respectively. For instance, position V13 presents 6 mutations (for amino acids P, L, G, F, D, A) rendering a wild-type phenotype and 1 mutation (V13I) rendering a mutant phenotype. It may be noticed that all positions tolerate most substitutions (Tolerance/Intolerance > 1). The last column show the substitutions observed for every position in the multiple sequence alignment (MSA) generated with TCOFFEE (see Methods); the letters in bold in this last column mark mutations identified in our experimental mutagenesis.

Table S5. Multiple mutations in HokC with wild type phenotype.

Mutation	***Count***
**M7W,I12S**	613
**I12S,I14S**	317
**L11P,I12S**	276
**M7W,I12C**	221
**M7W,I14S**	220
**M7W,L11P**	184
**I12S,V19G**	145
**I12S,A22T**	136
**I12S,V13I**	99
**I12C,I14S**	80
**M7W,A22T**	79
**I12S,I14P**	74
**I14S,V19G**	72
**M7W,V19G**	71
**I12S,A22S**	70
**L11P,I14S**	66
**I12S,V19L**	64

**Q3L,I12S**	60
**M7W,V13I**	49
**M7W,I14P**	48
**K2M,I12S**	44
**I12S,A21T**	44
**M7K,I12S**	42
**I12C,A22T**	39
**V13I,I14S**	38
**I12C,V13I**	37
**I14S,A22T**	36
**H4Y,I12S**	36
**M7W,A22S**	33
**M7W,I12S,I14S**	31
**I12C,V19G**	30
**I14S,V19L**	28
**H4Y,M7W**	28
**V13I,V19G**	26
**Q3L,I12C**	26
**M7K,I14S**	25
**I12C,A22S**	25
**Q3L,M7W**	24
**A6T,I12S**	24
**Q3L,I14S**	23
**K2M,M7W**	23
**V19L,A21T**	22
**L11P,V19G**	22
**L11P,V13I**	22
**L11P,I14P**	22
**I14S,A21T**	22
**I12C,I14P**	21

**H4Y,I12C**	21
**K2M,V19G**	20
**A18G,V19G**	20
**V13I,A22T**	19
**Q3L,V13I**	19
**Q3L,L11P**	18
**K2M,Q3L**	18
**V13I,A22S**	17
**L11P,I12C**	17
**K2M,H4Y**	17
**Q3L,V19L**	16
**K2M,V19L**	16
**I14S,A22S**	16
**Q3L,A21T**	15
**K2M,I14S**	15
**K2M,I12C**	15
**H4Y,I14S**	15
**M7K,V19L**	14
**M7K,V13I**	14
**V13I,V19L**	13
**Q3L,V19G**	13
**M7W,V19L**	13
**M7K,V19G**	13
**I12C,V19L**	13
**H4Y,V13I**	13
**Q3L,H4Y**	11
**M7W,I12C,I14S**	11
**H4Y,L11P**	11
**M7W,L11P,I12S**	10
**L11P,V19L**	10

**K2M,V13I**	10
**A6T,I14S**	10
**V13I,A21T**	9
**M7K,L11P**	9
**M7K,A22T**	9
**I12C,A21T**	9
**H4Y,A22T**	9
**M7K,I12C**	8
**L11P,A22S**	8
**L11P,A21T**	8
**K2M,L11P**	8
**I12S,I14S,V19G**	8
**A6T,I12C**	8
**A6T,A21T**	8
**V19L,A22T**	7
**M7W,I12S,V13I**	7
**M7W,A21T**	7
**L11P,I12S,I14S**	7
**K2M,I14P**	7
**H4Y,V19G**	7
**A6T,V19G**	7
**A6T,V13I**	7
**Q3L,I14P**	6
**Q3L,A22T**	6
**Q3L,A22S**	6
**M7W,I12S,A22T**	6
**M7K,A21T**	6
**L11P,A22T**	6
**I12S,V13I,I14S**	6
**H4Y,M7W,I14S**	6

**H4Y,A22S**	6
**A6T,L11P**	6
**V19L,A22S**	5
**Q3L,M7K**	5
**M7W,L11P,I14S**	5
**M7W,I12C,V13I**	5
**L11P,I12S,V19G**	5
**K2M,M7K**	5
**I14P,V19G**	5
**I12S,I14S,A22T**	5
**I12S,A18G**	5
**V13I,I14P**	4
**Q3L,A6T**	4
**M7W,L11P,V13I**	4
**M7W,I12S,I14P**	4
**M7W,I12C,A22T**	4
**L11P,I12S,V13I**	4
**K2M,I12S,I14S**	4
**K2M,A22S**	4
**H4Y,V19L**	4
**H4Y,I14P**	4
**A6T,M7W**	4
**A6T,I14P**	4
**A6T,A22T**	4
**A6T,A22S**	4
**V19G,A21T**	3
**Q3L,M7W,I12C**	3
**Q3L,L11P,I12S**	3
**Q3L,I12S,V13I**	3
**Q3L,I12S,I14S**	3

**M7W,V13I,I14S**	3
**M7W,I12S,V19G**	3
**M7W,I12S,A22S**	3
**M7W,I12C,V19G**	3
**M7K,I14P**	3
**M7K,A22S**	3
**M7K,A18G,V19G**	3
**K2M,M7W,I12S**	3
**K2M,A22T**	3
**K2M,A21T**	3
**I14S,A18G**	3
**I12S,V13I,A21T**	3
**I12S,I14S,V19L**	3
**I12D,V13I**	3
**I12C,V13I,I14S**	3
**H4Y,M7W,I12S**	3
**H4Y,A21T**	3
**A6T,M7W,I12S**	3
**A6T,I12S,I14S**	3
**A21T,A22T**	3
**V19G,A22S**	2
**V13I,I14S,A22T**	2
**Q3L,V13I,I14S**	2
**Q3L,M7W,I14S**	2
**Q3L,M7W,I12S**	2
**Q3L,L11P,V19G**	2
**Q3L,I12S,V19G**	2
**Q3L,A18G**	2
**M7W,I14S,V19G**	2
**M7W,I14S,A22T**	2

**M7W,I14S,A22S**	2
**M7W,I12D**	2
**M7K,V13I,A21T**	2
**M7K,A18G**	2
**L11P,I12S,V19L**	2
**K2M,M7W,L11P**	2
**I14P,A22S**	2
**I12D,I14S**	2
**H4Y,M7K**	2
**H4Y,I12S,I14S**	2
**H4Y,I12S,A22T**	2
**H4Y,A6T**	2
**A6T,V19L**	2
**A6T,M7K**	2
**A6T,I12S,V13I**	2
**A18G,V19L**	2
**A18G,A22S**	2
**V19G,A22T**	1
**V13I,I14S,V19L**	1
**V13I,I14S,A22S**	1
**V13I,A18G**	1
**Q3L,V19L,A21T**	1
**Q3L,V13I,V19L**	1
**Q3L,M7W,V19L**	1
**Q3L,M7W,V13I**	1
**Q3L,M7W,L11P**	1
**Q3L,M7W,I12S,I14S**	1
**Q3L,M7W,A22T**	1
**Q3L,M7K,L11P**	1
**Q3L,M7K,I14S**	1

**Q3L,M7K,I12S**	1
**Q3L,L11P,V13I**	1
**Q3L,I14S,A21T**	1
**Q3L,I12S,A21T**	1
**Q3L,I12C,V19L**	1
**Q3L,I12C,V19G**	1
**Q3L,H4Y,M7W,I12S**	1
**Q3L,H4Y,I12C**	1
**Q3L,H4Y,A22T**	1
**Q3L,A6T,M7K**	1
**Q3L,A6T,I12S**	1
**Q3L,A6T,I12D**	1
**M7W,V13I,I14P**	1
**M7W,V13I,A22T**	1
**M7W,L11P,V19G**	1
**M7W,L11P,I14P**	1
**M7W,L11P,A22T**	1
**M7W,I12S,V19L**	1
**M7W,I12S,V13I,A22T**	1
**M7W,I12S,I14S,A22T**	1
**M7W,I12S,A18G**	1
**M7W,I12D,V19G**	1
**M7W,I12C,V19L**	1
**M7W,I12C,I14P**	1
**M7W,I12C,A22S**	1
**M7W,A21T,A22S**	1
**M7K,V13I,A22S**	1
**M7K,L11P,I12S**	1
**M7K,I14S,V19G**	1
**M7K,I12S,V19L**	1

**M7K,I12S,V13I**	1
**M7K,I12S,A21T**	1
**M7K,I12D**	1
**M7K,I12C,V13I**	1
**L11P,V19L,A22S**	1
**L11P,V13I,V19G**	1
**L11P,V13I,I14S**	1
**L11P,I14S,V19G**	1
**L11P,I12S,V13I,I14S**	1
**L11P,I12S,I14P**	1
**L11P,I12D,V13I**	1
**L11P,I12D**	1
**L11P,A18G,V19G**	1
**K2M,V13I,A22S**	1
**K2M,Q3L,V19L**	1
**K2M,Q3L,I12S,V19G**	1
**K2M,Q3L,H4Y,V19L**	1
**K2M,Q3L,A21T**	1
**K2M,M7W,V19G**	1
**K2M,M7W,I14S**	1
**K2M,M7W,I12C**	1
**K2M,L11P,I14S**	1
**K2M,L11P,I14P**	1
**K2M,I14S,V19L**	1
**K2M,I12S,V19L**	1
**K2M,I12S,V19G**	1
**K2M,I12S,I14S,V19G**	1
**K2M,I12S,A21T**	1
**K2M,I12D**	1
**K2M,I12C,V13I**	1

**K2M,I12C,I14S**	1
**K2M,I12C,I14P**	1
**K2M,H4Y,V19L**	1
**K2M,H4Y,V19G**	1
**K2M,H4Y,V13I**	1
**K2M,H4Y,L11P**	1
**K2M,H4Y,I12S**	1
**K2M,H4Y,I12C**	1
**K2M,H4Y,A21T**	1
**K2M,A6T,A22T**	1
**K2M,A6T**	1
**I14S,V19L,A21T**	1
**I14S,A18G,V19G**	1
**I14P,V19L**	1
**I14P,A22T**	1
**I12S,V19L,A22T**	1
**I12S,V19L,A21T**	1
**I12S,V13I,A22S**	1
**I12S,I14S,A22S**	1
**I12S,A18G,V19G**	1
**I12D,V19L**	1
**I12D,V19G**	1
**I12D,I14P**	1
**I12D,A22T**	1
**I12D,A21T**	1
**I12C,I14P,V19G**	1
**H4Y,V13I,A22S**	1
**H4Y,M7W,L11P**	1
**H4Y,L11P,I14S**	1
**H4Y,L11P,I12S,V19G**	1

H4Y,L11P,I12D,V13I	1
H4Y,I12S,V19G	1
H4Y,I12S,V13I	1
H4Y,A6T,V13I	1
A6T,V13I,A22T	1
A6T,V13I,A22S	1
A6T,L11P,V13I	1
A6T,L11P,I14S	1
A6T,L11P,I12S	1
A6T,I12S,I14S,V19G	1
A6T,I12S,I14P	1
A6T,I12S,A22S	1
A6T,I12S,A21T	1
A6T,I12D,V13I	1
A6T,I12C,A22S	1
A6T,A18G	1
A18G,A21T	1

Table S6. Occurrence of deleterious single-point mutations in compensatory mutations.

Intolerated single-point mutation	Occurrence
K2M	46
Q3L	51
H4Y	39
A6T	36
M7K	30
M7W	65
L11P	52
I12C	35

**I12D**	16
**I12S**	79
**V13I**	58
**I14P**	24
**I14S**	59
**A18G**	15
**V19G**	43
**V19L**	36
**A21T**	29
**A22T**	31
**A22S**	29

The value Occurrence reports the number of multiple compensatory mutations that presented the particular single-point mutation reported in column labeled "Intolerated single-point mutation".

Table S7. Multiple sequence alignment of 148 protein sequences from PF01848 family obtained with TM-COFFEE.

IN	HEL	OUT

```
HOKC_ECOLI    ------------------------------------------------MK----------------
F3VWM0_SHIBO  ------------------------------------------------MS----------------
H5R5N6_ECOLX  ------------------------------------------------ML----------------
C6UVK1_ECO5T  ------------------------------------------------ML----------------
HOKF_ECO57    M------------------------------------------------L----------------
H4V506_ECOLX  M------------------------------------------------L----------------
E2QGR9_ECOLX  ------------------------------------------------MP----------------
D6J7Q5_ECOLX  ------------------------------------------------ML----------------
F4TJV8_ECOLX  M------------------------------------------------L----------------
G0F2P1_ECOLX  ------------------------------------------------MP----------------
G1Z0S1_ECOLX  M------------------------------------------------L----------------
G1YEQ3_ECOLX  M------------------------------------------------L----------------
F9CTD1_ECOLX  ------------------------------------------------MS----------------
H4IYN1_ECOLX  ------------------------------------------------MP----------------
F5NNJ6_SHIFL  ------------------------------------------------MP----------------
E1J5E3_ECOLX  ------------------------------------------------ML----------------
B7N6Z5_ECOLU  M------------------------------------------------L----------------
H1FMW9_ECOLX  ------------------------------------------------ML----------------
F9CSC2_ECOLX  ------------------------------------------------AM----------------
H8D654_ECOLX  ------------------------------------------------ML----------------
E9TXJ2_ECOLX  ------------------------------------------------ML----------------
H5J5L9_ECOLX  M------------------------------------------------L----------------
H5NSR7_ECOLX  ------------------------------------------------MS----------------
E2X956_SHIDY  ------------------------------------------------ML----------------
D8E1P5_ECOLX  M------------------------------------------------L----------------
H5PSJ3_ECOLX  ------------------------------------------------MP----------------
D2AAD3_SHIF2  ------------------------------------------------MP----------------
H1DN41_ECOLX  MKPQDDISINP---------------GAATPNQTIWMVVPSSHGGNINML----------------
E9VJD7_ECOLX  ------------------------------------------------ML----------------
E9TYV6_ECOLX  ------------------------------------------------MQ----------------
```

```
F4UN92_ECOLX    ------------------------------------------------MKYLNTTDCSLFLAERSK
F8XK11_ECOLX    ------------------------------------------------ML----------------
G1ZVT9_ECOLX    ------------------------------------------------ML----------------
E9Y7R9_ECOLX    ------------------------------------------------M-----------------
H4LZX6_ECOLX    ------------------------------------------------MP----------------
D6HWC9_ECOLX    ------------------------------------------------ML----------------
E2KHD1_ECO57    ------------------------------------------------ML----------------
F9S7G0_9VIBR    ------------------------------------------------MP----------------
G5W2A4_ECOLX    ------------------------------------------------MS----------------
E8HUC2_ECOLX    ------------------------------------------------MK----------------
HOKD_ECO57      ------------------------------------------------MK----------------
B2U569_SHIB3    ------------------------------------------------MP----------------
H9YDF6_ECOKO    ------------------------------------------------MP----------------
E1IYD1_ECOLX    ------------------------------------------------MK----------------
G2FAK9_ECOLX    ------------------------------------------------ML----------------
E9XTT2_ECOLX    ------------------------------------------------MW----------------
E8II98_ECOLX    MPEAFRFQVYSEVWKDEAPGDISINPGAATPNQTIWMVVPSSHGGNINML----------------
G5Y5U9_ECOLX    ------------------------------------------------ML----------------
B3HJT0_ECOLX    ------------------------------------------------ML----------------
E9U427_ECOLX    ------------------------------------------------ML----------------
D8E8Z0_ECOLX    MKPQEIFLSIL---------------GLPLRASTTRMVVPSSHGGNINML----------------
G2S0L6_ENTAL    ------------------------------------------------MT----------------
H5JJ49_ECOLX    ------------------------------------------------MS----------------
E1PB25_ECOAB    MVV------------------------------------PSSHGGNINML----------------
H5GVQ2_ECOLX    M------------------------------------------------L----------------
E7HA11_ECOLX    ------------------------------------------------MP----------------
G8XCJ0_KLEPN    ------------------------------------------------MK----------------
H4JR53_ECOLX    M------------------------------------------------L----------------
G5XXC8_ECOLX    ------------------------------------------------MS----------------
Q3Z5Z9_SHISS    ------------------------------------------------ML----------------
E0IZ09_ECOLW    ------------------------------------------------ML----------------
G5KK22_ECOLX    ------------------------------------------------ML----------------
H6MBN5_ECOLX    ------------------------------------------------MK----------------
D5CWF7_ECOKI    ------------------------------------------------ML----------------
B2TTD6_SHIB3    MKPQEIFLSIL---------------GLPLRASTTRMVVPSSHGGNINML----------------
D3H5D3_ECO44    ------------------------------------------------MK----------------
D6J6E3_ECOLX    ------------------------------------------------ML----------------
F5VK79_ENTSA    ------------------------------------------------MK----------------
B6ZP83_ECO57    M------------------------------------------------L----------------
B3I927_ECOLX    M------------------------------------------------L----------------
H5FWT6_ECOLX    ------------------------------------------------MS----------------
H5BXU3_ECOLX    ------------------------------------------------MP----------------
G5W9P6_ECOLX    M------------------------------------------------L----------------
D6IMK6_ECOLX    ------------------------------------------------MK----------------
H4Q809_ECOLX    ------------------------------------------------MP----------------
E5B936_ERWAM    ------------------------------------------------MK----------------
D8C2A4_ECOLX    ------------------------------------------------MP----------------
F9HSF5_ECOLX    ------------------------------------------------------------------
E8YBF8_ECOKO    ------------------------------------------------MK----------------
H6MHP8_ECOLX    M------------------------------------------------L----------------
F4VXG3_ECOLX    ------------------------------------------------MN----------------
E1IRH6_ECOLX    ------------------------------------------------ML----------------
H5NFV4_ECOLX    ------------------------------------------------MP----------------
H7CRD5_ECO57    ------------------------------------------------MK----------------
F1XMB4_ECO57    ------------------------------------------------MP----------------
E8I9N6_ECOLX    ------------------------------------------------MK----------------
C8TWH1_ECO26    ------------------------------------------------MKYLNTTDCSLFLAERSK
E1IQK3_ECOLX    M------------------------------------------------L----------------
B2NP37_ECO57    ------------------------------------------------MP----------------
D6GL38_9ENTR    MHEHHKSS----------------------------LLLVENQHWRRGMP----------------
G5TUD2_ECOLX    ------------------------------------------------ML----------------
E7TAB1_SHIFL    ------------------------------------------------M-----------------
E9Y8K4_ECOLX    ------------------------------------------------MI----------------
H1F377_ECOLX    ------------------------------------------------ML----------------
F8XED9_ECOLX    ------------------------------------------------MP----------------
F7MUV5_ECOLX    ------------------------------------------------MS----------------
H4NJR3_ECOLX    ------------------------------------------------MP----------------
H4KT12_ECOLX    ------------------------------------------------MK----------------
H1FAP9_ECOLX    ------------------------------------------------ML----------------
F9CEP4_ECOLX    ------------------------------------------------ML----------------
B2U6D2_ECOLX    ------------------------------------------------MK----------------
B6ZRV0_ECO57    ------------------------------------------------MP----------------
B3X849_ECOLX    ------------------------------------------------MP----------------
E1I0A5_ECOLX    ------------------------------------------------MK----------------
H5A0H9_ECOLX    M------------------------------------------------L----------------
G5X3P4_ECOLX    ------------------------------------------------ML----------------
H5KI64_ECOLX    M------------------------------------------------L----------------
```

```
G5WI34_ECOLX   ------------------------------------------------MM----------------
F3Q9V6_9ENTR   ------------------------------------------------------------------
D8B2D1_ECOLX   ------------------------------------------------MN----------------
E9VCC9_ECOLX   ------------------------------------------------MK----------------
H5AV32_ECOLX   ------------------------------------------------MS----------------
F3V7U1_SHIDY   ------------------------------------------------MS----------------
E9Y8K6_ECOLX   MGL-------------------------PLQTRTIWMVVPSSHGGNINML----------------
H4YAG3_ECOLX   ------------------------------------------------------------------
E1IRH4_ECOLX   MKPQEIFLSIL---------------GLPLRASTTRMVVPSSHGGNINML----------------
G5UXX9_ECOLX   MPEHHKVS----------------------------LLLVGNQHRGLGMP----------------
C6UR89_ECO5T   ------------------------------------------------ML----------------
Q0H0B2_ECOLX   ------------------------------------------------M-----------------
C8SY02_KLEPR   ------------------------------------------------ML----------------
H5H178_ECOLX   ------------------------------------------------MK----------------
C8TPL3_ECO26   ------------------------------------------------ML----------------
H9YME7_ECOKO   ------------------------------------------------ML----------------
G5XGE5_ECOLX   M------------------------------------------------L----------------
Q8Z607_SALTI   ------------------------------------------------MK----------------
I0QXL4_9ENTR   ------------------------------------------------MP----------------
G5WKB9_ECOLX   MPEHHKVS----------------------------LLLVGNQHRGLGMP----------------
F4UZC8_ECOLX   MKPQDDISINP---------------GAATPNQTIWMVVPSSHGGNINML----------------
H3M9M9_KLEOX   MNLNNSDV---------------------------SLFF----AGRQDML----------------
B3H9D0_ECOLX   ------------------------------------------------MS----------------
F1XSU4_ECO57   M------------------------------------------------L----------------
D3RCX9_KLEVT   ------------------------------------------------MP----------------
H4FPV2_ECOLX   ------------------------------------------------ML----------------
F9CJU5_ECOLX   ------------------------------------------------MS----------------
H5JUR0_ECOLX   ------------------------------------------------ML----------------
H5AS66_ECOLX   ------------------------------------------------MP----------------
C8UQU5_ECO1A   ------------------------------------------------ML----------------
G5X8B3_ECOLX   ------------------------------------------------MS----------------
H4MLQ7_ECOLX   M------------------------------------------------L----------------
E7JA92_ECOLX   MPEHHKVS----------------------------LLLVGNQHRGLGMP----------------
G0GSM6_KLEPN   M------------------------------------------------L----------------
G9RE52_9ENTR   M------------------------------------------------L----------------
H5CWM5_ECOLX   ------------------------------------------------MK----------------
G7RV84_KLEPN   ------------------------------------------------MK----------------
HOKC_SHIFL     ------------------------------------------------MK----------------
B3AGC6_ECO57   ------------------------------------------------MP----------------
H8WUZ6_SALTS   ------------------------------------------------------------------
B5R4Z4_SALEP   ------------------------------------------------ML----------------
E8H236_ECO57   ------------------------------------------------MK----------------
G2AM71_ECOLX   ------------------------------------------------MP----------------
```

```
HOKC_ECOLI     -------------------------------------------QHKA-------------------
F3VWM0_SHIBO   -------------------------------------------Q-KS-------------------
H5R5N6_ECOLX   --T------------------------------------------KY-------------------
C6UVK1_ECO5T   --NTC--------------------R-VPLTDRKVKEKRAMK-QHKV-------------------
HOKF_ECO57     --T------------------------------------------KY-------------------
H4V506_ECOLX   --T------------------------------------------KY-------------------
E2QGR9_ECOLX   --Q------------------------------------------KY-------------------
D6J7Q5_ECOLX   --T------------------------------------------KY-------------------
F4TJV8_ECOLX   --T------------------------------------------KY-------------------
G0F2P1_ECOLX   --Q------------------------------------------KY-------------------
G1Z0S1_ECOLX   --T------------------------------------------KY-------------------
G1YEQ3_ECOLX   --T------------------------------------------KY-------------------
F9CTD1_ECOLX   -------------------------------------------Q-KS-------------------
H4IYN1_ECOLX   --Q------------------------------------------KY-------------------
F5NNJ6_SHIFL   --Q------------------------------------------KY-------------------
E1J5E3_ECOLX   --NTC--------------------R-VPLTDRKVKEKRAMK-QHKA-------------------
B7N6Z5_ECOLU   --T------------------------------------------KY-------------------
H1FMW9_ECOLX   --T------------------------------------------KY-------------------
F9CSC2_ECOLX   -------------------------------------------SQKS-------------------
H8D654_ECOLX   --T------------------------------------------KY-------------------
E9TXJ2_ECOLX   --HKRR---------------------LASYEPKGKEKQAMK-QQKA-------------------
H5J5L9_ECOLX   --T------------------------------------------KY-------------------
H5NSR7_ECOLX   -------------------------------------------Q-KS-------------------
E2X956_SHIDY   --T------------------------------------------KY-------------------
D8E1P5_ECOLX   --T------------------------------------------KY-------------------
H5PSJ3_ECOLX   --Q------------------------------------------KY-------------------
D2AAD3_SHIF2   --Q------------------------------------------KY-------------------
H1DN41_ECOLX   --T------------------------------------------KY-------------------
E9VJD7_ECOLX   --T------------------------------------------KY-------------------
E9TYV6_ECOLX   --NTC--------------------R-VPLTDRKVKEKRAMK-QHKA-------------------
F4UN92_ECOLX   FMT------------------------------------------KY-------------------
```

```
F8XK11_ECOLX   --YTRR---------------------LASYVPKGKEKQAMK-QQKA-------------------
G1ZVT9_ECOLX   --T------------------------------------------KY-------------------
E9Y7R9_ECOLX   -------------------------------------------PQRT-------------------
H4LZX6_ECOLX   --Q------------------------------------------KY-------------------
D6HWC9_ECOLX   --CTCR---------------------LASYAPEGKEKQAMK-QQKA-------------------
E2KHD1_ECO57   --T------------------------------------------KY-------------------
F9S7G0_9VIBR   -------------------------------------------RNKT-------------------
G5W2A4_ECOLX   --HKCR---------------------LASYVPKGKGKQAMK-QQKA-------------------
E8HUC2_ECOLX   -------------------------------------------QHKV-------------------
HOKD_ECO57     -------------------------------------------QQKA-------------------
B2U569_SHIB3   --Q------------------------------------------KY-------------------
H9YDF6_ECOKO   --Q------------------------------------------KY-------------------
E1IYD1_ECOLX   --------------------------------------------HNP-------------------
G2FAK9_ECOLX   --NTC--------------------R-VPLTDRKVKEKRAMK-QHKA-------------------
E9XTT2_ECOLX   --QKDKSPVVNFSLTHEAPACLDNIR-IASYRALRKEKKAMKLPRSS-------------------
E8II98_ECOLX   --T------------------------------------------KY-------------------
G5Y5U9_ECOLX   --NTC--------------------R-VPLTDRKVKEKRAMK-QHKA-------------------
B3HJT0_ECOLX   --T------------------------------------------KY-------------------
E9U427_ECOLX   --T------------------------------------------KY-------------------
D8E8Z0_ECOLX   --T------------------------------------------KY-------------------
G2S0L6_ENTAL   -------------------------------------------PLKT-------------------
H5JJ49_ECOLX   -------------------------------------------Q-KS-------------------
E1PB25_ECOAB   --T------------------------------------------KY-------------------
H5GVQ2_ECOLX   --T------------------------------------------KY-------------------
E7HA11_ECOLX   -------------------------------------------Q-KT-------------------
G8XCJ0_KLEPN   ------------------------------------------LPRNT-------------------
H4JR53_ECOLX   --T------------------------------------------KY-------------------
G5XXC8_ECOLX   -------------------------------------------Q-KS-------------------
Q3Z5Z9_SHISS   --NTC--------------------R-VPLTDRKVKEKRAMK-QHKA-------------------
E0IZ09_ECOLW   --NTC--------------------R-VPLTDRKVKEKRAMK-QHKA-------------------
G5KK22_ECOLX   --T------------------------------------------KY-------------------
H6MBN5_ECOLX   -------------------------------------------QQKA-------------------
D5CWF7_ECOKI   --T------------------------------------------KY-------------------
B2TTD6_SHIB3   --T------------------------------------------KY-------------------
D3H5D3_ECO44   --L----------------------------------------PGNA-------------------
D6J6E3_ECOLX   --NTC--------------------R-VPLTDRKVKEKRAMK-QHKA-------------------
F5VK79_ENTSA   -------------------------------------------PLHY-------------------
B6ZP83_ECO57   --T------------------------------------------KY-------------------
B3I927_ECOLX   --T------------------------------------------KY-------------------
H5FWT6_ECOLX   -------------------------------------------Q-KS-------------------
H5BXU3_ECOLX   --Q------------------------------------------KY-------------------
G5W9P6_ECOLX   --T------------------------------------------KY-------------------
D6IMK6_ECOLX   -------------------------------------------QQKA-------------------
H4Q809_ECOLX   --Q------------------------------------------KY-------------------
E5B936_ERWAM   ------------------------------------------LPANC-------------------
D8C2A4_ECOLX   --Q------------------------------------------KY-------------------
F9HSF5_ECOLX   ------------------------------------------------------------------
E8YBF8_ECOKO   --Q-----------------------------------------QKA-------------------
H6MHP8_ECOLX   --T------------------------------------------KY-------------------
F4VXG3_ECOLX   --GKSR---------------------LASYVPKGKEKQAMK-QQKA-------------------
E1IRH6_ECOLX   --T------------------------------------------KY-------------------
H5NFV4_ECOLX   --Q------------------------------------------KY-------------------
H7CRD5_ECO57   ------------------------------------------LPRSS-------------------
F1XMB4_ECO57   -------------------------------------------Q-KT-------------------
E8I9N6_ECOLX   -------------------------------------------QQKA-------------------
C8TWH1_ECO26   FMT------------------------------------------KY-------------------
E1IQK3_ECOLX   --T------------------------------------------KY-------------------
B2NP37_ECO57   -------------------------------------------Q-KT-------------------
D6GL38_9ENTR   --Q------------------------------------------KY-------------------
G5TUD2_ECOLX   --T------------------------------------------KY-------------------
E7TAB1_SHIFL   -----------------------------------------K-QHKA-------------------
E9Y8K4_ECOLX   --FYYQ---------PEAPYMLNTCR-VPLTDRKVKEKRAMK-QHKA-------------------
H1F377_ECOLX   --NTC--------------------R-VPLTDRKVKEKRAMK-QHKA-------------------
F8XED9_ECOLX   --Q------------------------------------------KY-------------------
F7MUV5_ECOLX   -------------------------------------------Q-KS-------------------
H4NJR3_ECOLX   -------------------------------------------Q-KT-------------------
H4KT12_ECOLX   -------------------------------------------QHKA-------------------
H1FAP9_ECOLX   --DTCR---------------------LASYVPKGKEKQAMK-QQKA-------------------
F9CEP4_ECOLX   --T------------------------------------------KY-------------------
B2U6D2_ECOLX   --L----------------------------------------PGNA-------------------
B6ZRV0_ECO57   --Q------------------------------------------KY-------------------
B3X849_ECOLX   --Q------------------------------------------KY-------------------
E1I0A5_ECOLX   --------------------------------------------HNP-------------------
H5A0H9_ECOLX   --T------------------------------------------KY-------------------
G5X3P4_ECOLX   --NTC--------------------R-VPLTDRKVKEKRAMK-QHKA-------------------
H5KI64_ECOLX   --T------------------------------------------KY-------------------
G5WI34_ECOLX   -------------------------------------------PQRT-------------------
```

```
F3Q9V6_9ENTR   ------------------------------------------------------------------
D8B2D1_ECOLX   --GKSR---------------------LASYVPKGKEKQAMK-QQKA-------------------
E9VCC9_ECOLX   -------------------------------------------QHKA-------------------
H5AV32_ECOLX   -------------------------------------------Q-KS-------------------
F3V7U1_SHIDY   -------------------------------------------Q-KS-------------------
E9Y8K6_ECOLX   --T------------------------------------------KY-------------------
H4YAG3_ECOLX   ------------------------------------------------------------------
E1IRH4_ECOLX   --T------------------------------------------KY-------------------
G5UXX9_ECOLX   --Q------------------------------------------KY-------------------
C6UR89_ECO5T   --DTCR---------------------LASYAPKGKEKQAMK-QQKA-------------------
Q0H0B2_ECOLX   --T------------------------------------------KY-------------------
C8SY02_KLEPR   --HQHQVTPLLT------------LRYLQYLCELTQEKKAMKLPRNA-------------------
H5H178_ECOLX   -------------------------------------------QHKA-------------------
C8TPL3_ECO26   --HKRR---------------------LASYAPKGKEKQVMK-QQKA-------------------
H9YME7_ECOKO   --T------------------------------------------KY-------------------
G5XGE5_ECOLX   --T------------------------------------------KY-------------------
Q8Z607_SALTI   --Q-----------------------------------------QKA-------------------
I0QXL4_9ENTR   --T------------------------------------------NIKVAPHLLKRKGKHAMNSLM
G5WKB9_ECOLX   --Q------------------------------------------KY-------------------
F4UZC8_ECOLX   --T------------------------------------------KY-------------------
H3M9M9_KLEOX   --T------------------------------------------KY-------------------
B3H9D0_ECOLX   -------------------------------------------Q-KS-------------------
F1XSU4_ECO57   --T------------------------------------------KY-------------------
D3RCX9_KLEVT   --Q------------------------------------------KY-------------------
H4FPV2_ECOLX   --NTC--------------------R-VPLTDRKVKEKRAMK-QHKA-------------------
F9CJU5_ECOLX   -------------------------------------------Q-KS-------------------
H5JUR0_ECOLX   --T------------------------------------------KY-------------------
H5AS66_ECOLX   -------------------------------------------Q-KT-------------------
C8UQU5_ECO1A   --VTFRVA---------------------SYPLKCKEKKAMKLPNQP-------------------
G5X8B3_ECOLX   -------------------------------------------Q-KS-------------------
H4MLQ7_ECOLX   --T------------------------------------------KY-------------------
E7JA92_ECOLX   --Q------------------------------------------KY-------------------
G0GSM6_KLEPN   --T------------------------------------------KY-------------------
G9RE52_9ENTR   --T------------------------------------------KY-------------------
H5CWM5_ECOLX   -------------------------------------------QHKA-------------------
G7RV84_KLEPN   ------------------------------------------LPRNT-------------------
HOKC_SHIFL     -------------------------------------------QHKA-------------------
B3AGC6_ECO57   --Q------------------------------------------KY-------------------
H8WUZ6_SALTS   ------------------------------------------------------------------
B5R4Z4_SALEP   --CTCR---------------------LASYVPKGKEKQAMK-QQKA-------------------
E8H236_ECO57   -------------------------------------------QHKV-------------------
G2AM71_ECOLX   --Q------------------------------------------KY-------------------


HOKC_ECOLI     -MIVALIVICITAVVAALVTRKDLCEVHIRTGQTEVAVFTAYES--E-------------------
F3VWM0_SHIBO   -L--TAITFCVTAILIIWMLHGSLCEIRMSFWGAEFAAFLQCK---Q-------------------
H5R5N6_ECOLX   -ALAAVIVLCLTVLGFTLLVGDSLCEFTVKERNIEFKVVLAYEPK---------------------
C6UVK1_ECO5T   -MIVALIVICITAVVAALVTRKDLCEVHIRTGQTEVAVFTAYES--E-------------------
HOKF_ECO57     -ALVAVIVLCLTVPGFTLLVGDSLCEFTVKERNIEFRAVLAYEPK---------------------
H4V506_ECOLX   -ALVAIIVLCFTVLGFTLMVGDSLCELSIRERGMEFKAVLAYESK---------------------
E2QGR9_ECOLX   -RLLSLIVICFTLLFFTWMIRDSLCELHIKQESYELAAFLACKLKE--------------------
D6J7Q5_ECOLX   -ALAAVIVLCLTVLGFTLLVGDSLCEFTVKERNIEFKAVLAYEPK---------------------
F4TJV8_ECOLX   -ALVAIIVLCCTVLGFTLMVGDSLCELSIRERGMEFKAVLAYESK---------------------
G0F2P1_ECOLX   -RLLSLIVICFTLLFFTWMIRDSLCELHIKQESYELAAFLACKLKE--------------------
G1Z0S1_ECOLX   -ALAAVIVLCLTVLGFTLLVGDSLCEFTVKERNIEFKAVLAYEPK---------------------
G1YEQ3_ECOLX   -ALVAIIVLCCTVLGFTLMVGDSLCELSIRERGMEFKAVLAYESK---------------------
F9CTD1_ECOLX   -L--ITVTICMTVIFTIWMLHGSLCEFRLNLWGAEFAAFLQCK---Q-------------------
H4IYN1_ECOLX   -RLLSLIVICFTLLFFTWMIRDSLCELHIKQESYELAAFLACKLKE--------------------
F5NNJ6_SHIFL   -RLLSLIVICFTLLFFTWMIRDSLCELHIKQESYELAAFLAYKLKE--------------------
E1J5E3_ECOLX   -MIVALIVICITAVVAALVTRKDLCEVHIRTGQTEVAVFTAYES--E-------------------
B7N6Z5_ECOLU   -ALVAIIVLCCTVLGFTLMVGDSLCELSIRERGMEFKAVLAYESK---------------------
H1FMW9_ECOLX   -ALVAVIVLCLTVLGFTLLVGDSLCEFTVKERNIEFKAVLAYEPK---------------------
F9CSC2_ECOLX   -L--TAIMFCVTVILIIWMLHGSLCEIRMSFWGAEFAAFLQCK---Q-------------------
H8D654_ECOLX   -ALVAVIVLCLTVLGFTLLVGDSLCEFTVKERNIEFKAVLAYEPK---------------------
E9TXJ2_ECOLX   -MLIALIVICLTVIVTALVTRKDLCEVRIRTGQTEVAVFTA-------------------------
H5J5L9_ECOLX   -ALVAIIVLCCTVLGFTLMVGDSLCELSIRERGMEFKAVLAYESK---------------------
H5NSR7_ECOLX   -L--TAITSCVTAILIIWMLHGSLCEIRMSFWGAEFAAFLQCK---Q-------------------
E2X956_SHIDY   -ALVAVIVLCLTVLGFTLLVGDSLCEFTVKERNIEFKAVLTYEPK---------------------
D8E1P5_ECOLX   -ALVAIIVLCCTVLGFTLMVGDSLCELSIRERGMEFKAVLAYESK---------------------
H5PSJ3_ECOLX   -RLLSLIVICFTLLFFTWMIRDSLCELHIKQGSYELAAFLACNLK---------------------
D2AAD3_SHIF2   -RLLSLIVICFTLLFFTWMIRDSLCELHIKQESYELAAFLAYKLKE--------------------
H1DN41_ECOLX   -ALVAVIVLCLTVLGFTLLVGDSLCEFTVKERNIEFKAVLAYEPK---------------------
E9VJD7_ECOLX   -ALVAVIVLCLTVLGFTLLVGDSLCEFTVKERNIEFKAVLAYEPK---------------------
E9TYV6_ECOLX   -MIVALIVICITAVVAALVTRKDLCEVHIRTGQTEVAVFTAYES--E-------------------
F4UN92_ECOLX   -ALIGLLAVCATVLCFLLIFRERLCELNIHRGNTVVQVTLAYEAR---------------------
F8XK11_ECOLX   -MLIALIVICLTVIVTALVTRKDLCEVRIRIGQTEVAVFTAYEPE-E-------------------
```

```
G1ZVT9_ECOLX    -ALVAVIVLCLTVLGFTLLVGDSLCEFTVKERNIEFKAVLAYEPK---------------------
E9Y7R9_ECOLX    -FLMMLIVICVTILCFVWMVRDSLCGLRLQQGNTVLVATLAYEVK-R-------------------
H4LZX6_ECOLX    -RLLSLIVICFTLLFFTWMIRDSLCELHIKQGSYELAAFLACNLK---------------------
D6HWC9_ECOLX    -MLIALIVICLTVIMTALVTRKDLCEVRIRTGQTEVAVFTAYES--E-------------------
E2KHD1_ECO57    -ALVAVIVLCLTVLGFTLLVGDSLCEFTVKERNIEFKAVLAYEPK---------------------
F9S7G0_9VIBR    -ALLGLIVICLTILCFTWMTRGTLCELHFKDGNSVISATLAYES--R-------------------
G5W2A4_ECOLX    -MLIALIVICLTVIVTALVTRKDLCEVRLRTGQTEVAVFTAYEPE-E-------------------
E8HUC2_ECOLX    -MIVALIVICITAVVAALVTRKDLCEVHIRTGQTEVAVFTAYES--E-------------------
HOKD_ECO57      -MLIALIVICLTVIVTALVTRKDLCEVRIRTGQTEVAVFTAYEPE-E-------------------
B2U569_SHIB3    -GLLSLIVICFTLLFFTWMVRDSLCELHIKQGRYELAAFLACNLKE--------------------
H9YDF6_ECOKO    -RLLSLIVICFTLLFFTWMIRDSLCELHIKQGSYELAAFLACNLKELESSAGSYSRLSYGVAHSH-
E1IYD1_ECOLX    -LVVCLLIICITILTFTLLTRQTLYELRFRDGDKEVAALMACTS--R-------------------
G2FAK9_ECOLX    -MIVALIVICITAVVAALVTRKDLCEVHIRTGQTEVAVFTAYES--E-------------------
E9XTT2_ECOLX    -LVWCVLIVCLTLLIFTYLTRKSLCEIRYRDGHREVAAFMAYESG-K-------------------
E8II98_ECOLX    -ALVAVIVLCLTVPGFTLLVGDSLCEFTVKERNIEFRAVLAYEPK---------------------
G5Y5U9_ECOLX    -MIVALIVICITAVVAALVTRKDLCEVHTRTGQTEVAVFTAYES--E-------------------
B3HJT0_ECOLX    -ALVAVIVLCLTVLGFTLLVGDSLCEFTVKERNIEFKAVLAYEPK---------------------
E9U427_ECOLX    -ALVAVIVLCLTVLGFTLLVGDSLCEFTVKERNIEFKAVLAYEPK---------------------
D8E8Z0_ECOLX    -ALVAVIVLCLTVLGFTLLVGDSLCEFTVKERNIEFKAVLAYEPK---------------------
G2S0L6_ENTAL    -VSGIVFIICLTIVIFTFINRGRLCELTIKSEHQEVAAKLACLA--G-------------------
H5JJ49_ECOLX    -L--TAITFCVTVILIIWMLHGSLCEIRMSFWGAEFAAFLQCK---Q-------------------
E1PB25_ECOAB    -ALVAVIVLCLTVLGFTLLVGDSLCEFTVKERNIEFKAVLAYEPK---------------------
H5GVQ2_ECOLX    -ALVAIIVLCCTVLGFTLMVGDSLCELSIRERGMEFKAVLAYESK---------------------
E7HA11_ECOLX    -I--IVGMLCLTMLLTVWVLHASPCEFRVSFMWSEIAAFLQCK---P-------------------
G8XCJ0_KLEPN    -LLGCVLIVCLTLLIFTYLTRKSLCEIRYRDTNREVAAFLAYESA-K-------------------
H4JR53_ECOLX    -ALVAIIVLCCTVLGFTLMVGDSLCELSIRERGMEFKAVLAYESK---------------------
G5XXC8_ECOLX    -L--TAITFCVTAILIIWMLHGSLCEIRMSFWGAEFAAFLQCK---Q-------------------
Q3Z5Z9_SHISS    -MIVALIVICITAVVAALVTRKDLCEVHIRTGQTEVAVFTAYES--E-------------------
E0IZ09_ECOLW    -MIVALIVICITAVVAALVTRKDLCEVHIRTGQTEVAVFTAYES--E-------------------
G5KK22_ECOLX    -ALVAVIVLCLTVLGFTLLVGDSLCEFTVKERNIEFKAVLAYEPK---------------------
H6MBN5_ECOLX    -MLIALIVICLTVIVTALVTRKDLCEVRIRTGQTEVAVFTAYEPE-E-------------------
D5CWF7_ECOKI    -ALVAVIVLCLTVLGFTLLVGDSLCEFTVKERNIEFKAVLAYEPK---------------------
B2TTD6_SHIB3    -ALAAVIVLCLTVLGFTLLVGDSLCEFTVKERNIEFKAVLAYEPK---------------------
D3H5D3_ECO44    -LIWCVLIVCCTLLIFTFLTRNRLCEVRLKDGYREVTATMAYESGGK-------------------
D6J6E3_ECOLX    -MIVALIVICITAVVAALVTRKDLCEVHIRTGQTEVAVFTAYES--E-------------------
F5VK79_ENTSA    -LLACLFMVCVTILIFALMNQGTLCEHTIRSGSQEVAAKLACTG--K-------------------
B6ZP83_ECO57    -ALVAVIVLCLTVPGFTLLVGDSLCEFTVKERNIEFRAVLAYEPK---------------------
B3I927_ECOLX    -ALVAIIVLCCTVLGFTLMVGDSLCELSIRERGMEFKAVLAYESK---------------------
H5FWT6_ECOLX    -L--TAITFCVTAILIIWMLHGSLCEIRMSFWGAEFAAFLQCK---Q-------------------
H5BXU3_ECOLX    -RLLSLIVICFTLLFFTWMIRDSLCELHIKQGSYELAAFLACNLK---------------------
G5W9P6_ECOLX    -ALVAIIVLCCTVLGFTLMVGDSLCELSIRERGMEFKAVLAYESK---------------------
D6IMK6_ECOLX    -MLIALIVICLTVIVTALVTRKDLCEVRIRTGQTEVAVFTAYEPE-E-------------------
H4Q809_ECOLX    -RLLSLIVICFTLLFFTWMIRDSLCELHIKQGSYELAAFLACNLK---------------------
E5B936_ERWAM    -LIWCVLIVCVTLLIFTYLTRKSLCEIRYKDGYREVAAFMAYESG-K-------------------
D8C2A4_ECOLX    -RLLSLIVICFTLLFFTWMIRDSLCELHIKQESYELAAFLACKLKE--------------------
F9HSF5_ECOLX    ----------MTVLGFTLLVGDSLCEFTVKERNIEFKAVLAYEPK---------------------
E8YBF8_ECOKO    -MLIALIVICLTVIVTALVTRKDLCEVRIRTGQTEVAVFVDYESR-E-------------------
H6MHP8_ECOLX    -ALVAIIVLCCTVLGFTLMVGDSLCELSIRERGMEFKAVLAYESK---------------------
F4VXG3_ECOLX    -MLIALIVICLTVIVTALVTRKDLCEVRIRTGQTEVAVFTAYEPE-E-------------------
E1IRH6_ECOLX    -ALAAVIVLCLTVLGFTLLVGDSLCEFTVKERNIEFKVVLAYEPK---------------------
H5NFV4_ECOLX    -RLLSLIVICFTLLFFTWMIRDSLCELHIKQGSYELAAFLACNLK---------------------
H7CRD5_ECO57    -LVWCVLIVCLTLLIFTYLTRKSLCEIRYRDGYREVAAFMAYESG-K-------------------
F1XMB4_ECO57    -I--IVGMLCLTMLLTVWVLHASPCEFRVSFMWSEIAAFLQCK---P-------------------
E8I9N6_ECOLX    -MLIALIVICLTVIVTALVTRKDLCEVRIRTGQTEVAVFTAYEPE-E-------------------
C8TWH1_ECO26    -ALIGLLAVCATVLCFSLIFRERLCELNIYRGNTVVQVTLAYEAR---------------------
E1IQK3_ECOLX    -ALVAIIVLCFTVLGFTLMVGDSLCELSIRERGMEFKAVLAYESK---------------------
B2NP37_ECO57    -I--IVGMLCLTMLLTVWVLHASPCEFRVSFMWSEIAAFLQCK---P-------------------
D6GL38_9ENTR    -LLFGLVVICFTILLLTWMVRDSLCELQLRQGNIELVAFLACDIKQ--------------------
G5TUD2_ECOLX    -ALVAVIVLCLTVLGFTLLVGDSLCEFTVKERNIEFKAVLAYEPK---------------------
E7TAB1_SHIFL    -MIVALIVICITAVVAALVTRKDLCEVHIRTGQTEVAVFTAYES--E-------------------
E9Y8K4_ECOLX    -MIVALIVICITAVVAALVTRKDLCEVHIRTGQTEVAVFTAYES--E-------------------
H1F377_ECOLX    -MIVALIVICITAVVAALVTRKDLCEVHIRTGQTEVAVFTAYES--E-------------------
F8XED9_ECOLX    -RLLSLIVICFTLLFFTWMIRDSLCELHIKQGSYELAAFLACNLK---------------------
F7MUV5_ECOLX    -L--TAITFCVTVILIIWMLHGSLCEIRMSFWGAEFAAFLQCK---Q-------------------
H4NJR3_ECOLX    -I--IVGMLCLTMLLTVWVLHASPCEFRVSFMWSEIAAFLQCK---P-------------------
H4KT12_ECOLX    -MIVALIVICITAVVAALVTRKDLCEVHIRIGQTEVAVFTAYES--E-------------------
H1FAP9_ECOLX    -MLIALIVICLTVIVTALVTRKDLCEVRIRTGQTEVAVFTAYEPE-E-------------------
F9CEP4_ECOLX    -ALAAVIVLCLTVLGFTLLVGDSLCEFTVKERNIEFKVVLAYEPK---------------------
B2U6D2_ECOLX    -LIWCVLIVCCTLLIFTLLTRNRLCEVRLKDGYREVTATMAYESGGK-------------------
B6ZRV0_ECO57    -RLLSLIVICFTLLFFTWMIRDSLCELHIKQGSYELAAFLACNLK---------------------
B3X849_ECOLX    -RLLSLIVICFTLLFFTWMIRDSLCELHIKQESYELAAFLACKLKE--------------------
E1I0A5_ECOLX    -LVVCLLIICITILTFTLLTRQTLYELRFRDGDKEVAALMACTS--R-------------------
H5A0H9_ECOLX    -ALVAIIVLCCTVLGFTLMVGDSLCELSIRERGMEFKAVLAYESK---------------------
G5X3P4_ECOLX    -MIVALIVICITAVVAALVTRKDLCEVHTRTGQTEVAVFTAYES--E-------------------
H5KI64_ECOLX    -ALVAIIVLCCTVLGFTLMVGDSLCELSIRERGMEFKAVLAYESK---------------------
G5WI34_ECOLX    -FLMMLIVVCVTILCFVWMVRDSLCGLRLQQGNTVLVATLAYEVK-R-------------------
F3Q9V6_9ENTR    ---MAIIVLCITVLGFTLLVHSSLCELSIKERNIEFKAVLAYESK---------------------
```

```
D8B2D1_ECOLX    -MLIALIVICLTVIVTALVTRKDLCEVRIRTGQTEVAVFTAYEPE-E-------------------
E9VCC9_ECOLX    -MIVALIVICITAVVAALVTRKDLCEVHIRTGQTEVAVFTAYES--E-------------------
H5AV32_ECOLX    -L--TTITFCVTAILIIWMLHGSLCEIRMSFWGAEFAAFLQCK---Q-------------------
F3V7U1_SHIDY    -L--TAITFCVTVILIIWMLHGSLCEIRMSFWGAEFAAFLQCK---Q-------------------
E9Y8K6_ECOLX    -ALAAVIVLCLTVLGFTLLVGDSLCEFTVKERNIEFKAVLAYEPK---------------------
H4YAG3_ECOLX    --------MCATVLCFSLIFRERLCELNIHRGNTVVQVTLAYEARQCEVFQGQTPYIPETADPARA
E1IRH4_ECOLX    -ALVAVIVLCLTVLGFTLLVGDSLCEFTVKERNIEFKAVLAYEPK---------------------
G5UXX9_ECOLX    -RLLSLIVICFTLLFFTWMIRDSLCELHIKQGSYELAAFLACNLK---------------------
C6UR89_ECO5T    -MLIALIVICITVIVTALVTRKDLCEVRIRTGQTEVAVFTAYEPE-E-------------------
Q0H0B2_ECOLX    -ALIGVLAVCATVLCFLLIFRERLCELNIHRGNTVVQVTLAYEAR---------------------
C8SY02_KLEPR    -LVWCVLIVCLTLLIFTCLTRNRLCEVRLKDGNREVAASLAYESNGK-------------------
H5H178_ECOLX    -MIVALIVICITAVVAALVTRKDLCEVHIRTGQTEVAVFTAYES--E-------------------
C8TPL3_ECO26    -MLIALIVICLTVIVTALVTRKDLCEVRIRTGQTEVAVFTAYEPE-E-------------------
H9YME7_ECOKO    -ALVAVIVLCLTVLGFTLLVGDSLCEFTVKERNIEFKAVLAYEPK---------------------
G5XGE5_ECOLX    -ALVAIIVLCCTVLGFTLMVGDSLCELSIRERGMEFKAVLAYESK---------------------
Q8Z607_SALTI    -MLIALIVICLTVIVTALVTRKDLCEVRIRTGQTEVAVFVDYESR-E-------------------
I0QXL4_9ENTR    LFIAGLIVICVTLLSFTWMTRDSLCEIRFSDGIKEVVALMACETTR--------------------
G5WKB9_ECOLX    -RLLSLIVICFTLLFFTWMIRDSLCELHIKQGSYELAAFLACNLK---------------------
F4UZC8_ECOLX    -ALVAVIVLCLTVLGFTLLVGDSLCEFTVKERNIEFKAVLAYEPK---------------------
H3M9M9_KLEOX    -ALVAIVVLCITVLGFTLLVRSSLCELSIKERSMEFKAVLAYESK---------------------
B3H9D0_ECOLX    -L--TAITFCVTVILIIWMLHGSLCEIRMSFWGAEFAAFLQCK---Q-------------------
F1XSU4_ECO57    -ALVAVIVLCLTVPGFTLLVGDSLCEFTVKERNIEFRAVLAYEPK---------------------
D3RCX9_KLEVT    -LLFGLVVICFTILLLTWMVRDSLCELQLRQGNIELVAFLACDIKQ--------------------
H4FPV2_ECOLX    -MIVALIVICITAVVAALVTRKDLCEVHIRTGQTEVAVFTAYES--E-------------------
F9CJU5_ECOLX    -L--TAIMFCVTVIFXIWMLHGSLCEFRLNLWGAEFAAFLQCK---Q-------------------
H5JUR0_ECOLX    -ALAAVIVLCLTVLGFTLLVGDSLCEFTVKERNIEFKVVLAYEPK---------------------
H5AS66_ECOLX    -I--IVGMLCLTMLLTVWVLHASPCEFRVSFMWSEIAAFLQCK---P-------------------
C8UQU5_ECO1A    -VVLCVLIVCLTLLIFTWLTRNSLCELRLKDGTREVSAVMDYESG-K-------------------
G5X8B3_ECOLX    -L--TAITFCVTAILIIWMLHGSLCEIRMSFWGAEFAAFLQCK---Q-------------------
H4MLQ7_ECOLX    -ALVAVIVLCLTVPGFTLLVGDSLCEFTVKERNIEFRAVLAYEPK---------------------
E7JA92_ECOLX    -RLLSLIVICFTLLFFTWMIRDSLCELHIKQGSYELAAFLACNLK---------------------
G0GSM6_KLEPN    -ALVAIIVLCITVLGFTLLVHSSLCELSIKERNIEFKAVLAYESK---------------------
G9RE52_9ENTR    -ALVAIIVLCITVLGFTLLVHSSLCELSIKERNIEFKAVLAYESK---------------------
H5CWM5_ECOLX    -MIVALIVICITAVVAALVTRKDLCEVHIRTGQTEVAVFTAYES--E-------------------
G7RV84_KLEPN    -LLGCVLIVCLTLLIFTYLTRKSLCEIRYRDTNREVAAFMAYESA-K-------------------
HOKC_SHIFL      -MIVALIVICITAVVAALVTRKDLCEVHIRTGQTEVAVFTAYES--E-------------------
B3AGC6_ECO57    -RLLSLIVICFTLLFFTWMIRDSLCELHIKQGSYELAAFLACNLK---------------------
H8WUZ6_SALTS    ---MMLIVICVTILCFVWMVRDSLCGLRLQQGNTVLVATLAYEVK-R-------------------
B5R4Z4_SALEP    -MLIALIVICLTVIVTALVTRKDLCEVRIRTGQTEVAVFTAYEPE-E-------------------
E8H236_ECO57    -MIVALIVICITAVVAALVTRKDLCEVHIRTGQTEVAVFTAYES--E-------------------
G2AM71_ECOLX    -RLLSLIVICFTLLFFTWMIRDSLCELHIKQGSYELAAFLACNLK---------------------
                           *      :                . .


HOKC_ECOLI      ---------
F3VWM0_SHIBO    ---------
H5R5N6_ECOLX    --------K
C6UVK1_ECO5T    ---------
HOKF_ECO57      --------K
H4V506_ECOLX    --------K
E2QGR9_ECOLX    ---------
D6J7Q5_ECOLX    --------K
F4TJV8_ECOLX    --------K
G0F2P1_ECOLX    ---------
G1Z0S1_ECOLX    --------K
G1YEQ3_ECOLX    --------K
F9CTD1_ECOLX    ---------
H4IYN1_ECOLX    ---------
F5NNJ6_SHIFL    ---------
E1J5E3_ECOLX    ---------
B7N6Z5_ECOLU    --------K
H1FMW9_ECOLX    --------K
F9CSC2_ECOLX    ---------
H8D654_ECOLX    --------K
E9TXJ2_ECOLX    ---------
H5J5L9_ECOLX    --------K
H5NSR7_ECOLX    ---------
E2X956_SHIDY    --------K
D8E1P5_ECOLX    --------K
H5PSJ3_ECOLX    --------E
D2AAD3_SHIF2    ---------
H1DN41_ECOLX    --------K
E9VJD7_ECOLX    --------K
E9TYV6_ECOLX    ---------
F4UN92_ECOLX    --------K
F8XK11_ECOLX    ---------
G1ZVT9_ECOLX    --------K
```

```
E9Y7R9_ECOLX      ---------
H4LZX6_ECOLX      --------E
D6HWC9_ECOLX      ---------
E2KHD1_ECO57      --------K
F9S7G0_9VIBR      ---------
G5W2A4_ECOLX      ---------
E8HUC2_ECOLX      ---------
HOKD_ECO57        ---------
B2U569_SHIB3      ---------
H9YDF6_ECOKO      CTQIYFSQK
E1IYD1_ECOLX      ---------
G2FAK9_ECOLX      ---------
E9XTT2_ECOLX      ---------
E8II98_ECOLX      --------K
G5Y5U9_ECOLX      ---------
B3HJT0_ECOLX      --------K
E9U427_ECOLX      --------K
D8E8Z0_ECOLX      --------K
G2S0L6_ENTAL      ---------
H5JJ49_ECOLX      ---------
E1PB25_ECOAB      --------K
H5GVQ2_ECOLX      --------K
E7HA11_ECOLX      ---------
G8XCJ0_KLEPN      ---------
H4JR53_ECOLX      --------K
G5XXC8_ECOLX      ---------
Q3Z5Z9_SHISS      ---------
E0IZ09_ECOLW      ---------
G5KK22_ECOLX      --------K
H6MBN5_ECOLX      ---------
D5CWF7_ECOKI      --------K
B2TTD6_SHIB3      --------K
D3H5D3_ECO44      ---------
D6J6E3_ECOLX      ---------
F5VK79_ENTSA      ---------
B6ZP83_ECO57      --------K
B3I927_ECOLX      --------K
H5FWT6_ECOLX      ---------
H5BXU3_ECOLX      --------E
G5W9P6_ECOLX      --------K
D6IMK6_ECOLX      ---------
H4Q809_ECOLX      --------E
E5B936_ERWAM      ---------
D8C2A4_ECOLX      ---------
F9HSF5_ECOLX      --------K
E8YBF8_ECOKO      ---------
H6MHP8_ECOLX      --------K
F4VXG3_ECOLX      ---------
E1IRH6_ECOLX      --------K
H5NFV4_ECOLX      --------E
H7CRD5_ECO57      ---------
F1XMB4_ECO57      ---------
E8I9N6_ECOLX      ---------
C8TWH1_ECO26      --------K
E1IQK3_ECOLX      --------K
B2NP37_ECO57      ---------
D6GL38_9ENTR      ---------
G5TUD2_ECOLX      --------K
E7TAB1_SHIFL      ---------
E9Y8K4_ECOLX      ---------
H1F377_ECOLX      ---------
F8XED9_ECOLX      --------E
F7MUV5_ECOLX      ---------
H4NJR3_ECOLX      ---------
H4KT12_ECOLX      ---------
H1FAP9_ECOLX      ---------
F9CEP4_ECOLX      --------K
B2U6D2_ECOLX      ---------
B6ZRV0_ECO57      --------E
B3X849_ECOLX      ---------
E1I0A5_ECOLX      ---------
H5A0H9_ECOLX      --------K
G5X3P4_ECOLX      ---------
H5KI64_ECOLX      --------K
G5WI34_ECOLX      ---------
F3Q9V6_9ENTR      --------K
D8B2D1_ECOLX      ---------
```

```
E9VCC9_ECOLX    ---------
H5AV32_ECOLX    ---------
F3V7U1_SHIDY    ---------
E9Y8K6_ECOLX    --------K
H4YAG3_ECOLX    STQ-----V
E1IRH4_ECOLX    --------K
G5UXX9_ECOLX    --------E
C6UR89_ECO5T    ---------
Q0H0B2_ECOLX    --------K
C8SY02_KLEPR    ---------
H5H178_ECOLX    ---------
C8TPL3_ECO26    ---------
H9YME7_ECOKO    --------K
G5XGE5_ECOLX    --------K
Q8Z607_SALTI    ---------
I0QXL4_9ENTR    ---------
G5WKB9_ECOLX    --------E
F4UZC8_ECOLX    --------K
H3M9M9_KLEOX    --------K
B3H9D0_ECOLX    ---------
F1XSU4_ECO57    --------K
D3RCX9_KLEVT    ---------
H4FPV2_ECOLX    ---------
F9CJU5_ECOLX    ---------
H5JUR0_ECOLX    --------K
H5AS66_ECOLX    ---------
C8UQU5_ECO1A    ---------
G5X8B3_ECOLX    ---------
H4MLQ7_ECOLX    --------K
E7JA92_ECOLX    --------E
G0GSM6_KLEPN    --------K
G9RE52_9ENTR    --------K
H5CWM5_ECOLX    ---------
G7RV84_KLEPN    ---------
HOKC_SHIFL      ---------
B3AGC6_ECO57    --------E
H8WUZ6_SALTS    ---------
B5R4Z4_SALEP    ---------
E8H236_ECO57    ---------
G2AM71_ECOLX    --------E
```

Supplemental Table S8. ConSurf Results.

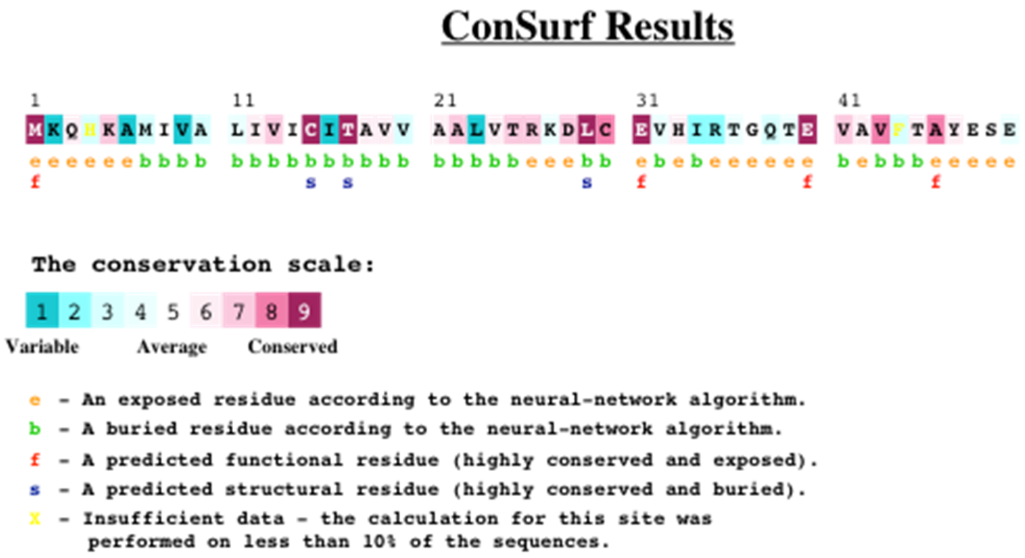

Table S9. Predictions of deleterious mutations in HokC family by PROVEAN.

Position

Variants

Score
Predicted mutation
1
M,A,M
-2.534
Deleterious
2
K,M,N,T,P,S,I,K,L,Q,W
0.264
Neutral
3
Q,Q,R,P,S
0.000
Neutral
4
H,Q,L,N,R,H,G,A
0.518
Neutral
5
K,K,H,R,Q,N,S
0.000
Neutral
6
A,V,T,P,S,C,Y,A,I
-1.188
Neutral
7
M,M,G,R,V,I,F,A,L
0.000
Neutral
8
I,I,V,L,S
0.000
Neutral
9
V,V,M,G,W,L,A,F,I
0.000
Neutral
10
A,T,S,M,G,A,I,C
-1.254
Neutral
11
L,L,T,V,A,I
0.000
Neutral
12
I,G,F,I,V,L
-6.218
Deleterious
13
V,A,M,I,V,T
-3.567
Deleterious
14
I,M,F,I,V,L,S
-2.495
Neutral

15
C,C
0.000
Neutral
16
I,C,F,I,A,M,V,L
-2.476
Neutral
17
T,T
0.000
Neutral
18
A,I,A,M,V,L
2.002
Neutral
19
V,I,L,V,P
0.741
Neutral
20
V,F,I,L,M,G,C,V,T,S
-2.136
Neutral
21
A,F,I,A,T,L,X
3.270
Neutral
22
A,V,T,L,S,A,I
0.901
Neutral
23
L,Y,L,W,F,C
0.127
Neutral
24
V,V,L,I,M
0.000
Neutral

Table S10. Multiple deleterious mutations found in sequences of the HokC family.

Uniprot sequence ID	*Intolerable mutations*
**G2AM71_ECOLX**	V19L, A22T
**G0F2P1_ECOLX**	V19L, A22T
**E9XTT2_ECOLX**	V13I, V19L, A22T
**G5KK22_ECOLX**	V19L, A22T
**B2TTD6_SHIB3**	V19L, A22T
**E2X956_SHIDY**	V19L, A22T

**H4IYN1_ECOLX**	V19L, A22T
**F9CEP4_ECOLX**	V19L, A22T
**H5A0H9_ECOLX**	V19L, A22T
**F9HSF5_ECOLX**	V19L, A22T
**H5AS66_ECOLX**	A6T, V19L, A21T
**E7JA92_ECOLX**	V19L, A22T
**E1IQK3_ECOLX**	V19L, A22T
**E9Y8K6_ECOLX**	V19L, A22T
**G8XCJ0_KLEPN**	A6T, V13I, V19L, A22T
**H5JUR0_ECOLX**	V19L, A22T
**H1FMW9_ECOLX**	V19L, A22T
**H5BXU3_ECOLX**	V19L, A22T
**G7RV84_KLEPN**	A6T, V13I, V19L, A22T
**C8TWH1_ECO26**	V19L, A22S
**G5W9P6_ECOLX**	V19L, A22T
**G2S0L6_ENTAL**	A6T, V13I, A22T
**H5R5N6_ECOLX**	V19L, A22T
**C8UQU5_ECO1A**	V13I, V19L, A22T
**E1PB25_ECOAB**	V19L, A22T
**E1I0A5_ECOLX**	V13I, V19L, A22T
**B2U6D2_ECOLX**	V13I, V19L, A22T
**G1Z0S1_ECOLX**	V19L, A22T
**H7CRD5_ECO57**	V13I, V19L, A22T
**D8E8Z0_ECOLX**	V19L, A22T
**H4JR53_ECOLX**	V19L, A22T
**B3X849_ECOLX**	V19L, A22T
**E2KHD1_ECO57**	V19L, A22T
**G1YEQ3_ECOLX**	V19L, A22T
**F8XED9_ECOLX**	V19L, A22T
**F4UZC8_ECOLX**	V19L, A22T

**C8SY02_KLEPR**	V13I, V19L, A22T
**D2AAD3_SHIF2**	V19L, A22T
**H9YME7_ECOKO**	V19L, A22T
**G5UXX9_ECOLX**	V19L, A22T
**E7HA11_ECOLX**	A6T, V19L, A21T
**E1IRH6_ECOLX**	V19L, A22T
**B3HJT0_ECOLX**	V19L, A22T
**B3AGC6_ECO57**	V19L, A22T
**D6J7Q5_ECOLX**	V19L, A22T
**E1IRH4_ECOLX**	V19L, A22T
**D8C2A4_ECOLX**	V19L, A22T
**E9VJD7_ECOLX**	V19L, A22T
**H4V506_ECOLX**	V19L, A22T
**D3RCX9_KLEVT**	V19L, A22T
**G5TUD2_ECOLX**	V19L, A22T
**B7N6Z5_ECOLU**	V19L, A22T
**H6MHP8_ECOLX**	V19L, A22T
**H4LZX6_ECOLX**	V19L, A22T
**G5XGE5_ECOLX**	V19L, A22T
**F3Q9V6_9ENTR**	V19L, A22T
**D3H5D3_ECO44**	V13I, V19L, A22T
**D6GL38_9ENTR**	V19L, A22T
**B3I927_ECOLX**	V19L, A22T
**H5PSJ3_ECOLX**	V19L, A22T
**E1IYD1_ECOLX**	V13I, V19L, A22T
**B6ZRV0_ECO57**	V19L, A22T
**I0QXL4_9ENTR**	V19L, A22T
**G9RE52_9ENTR**	V19L, A22T
**H3M9M9_KLEOX**	V19L, A22T
**F5NNJ6_SHIFL**	V19L, A22T

**E2QGR9_ECOLX**	V19L, A22T
**B2U569_SHIB3**	V19L, A22T
**H5J5L9_ECOLX**	V19L, A22T
**E5B936_ERWAM**	V13I, V19L, A22T
**H4YAG3_ECOLX**	V19L, A22S
**B2NP37_ECO57**	A6T, V19L, A21T
**G5WI34_ECOLX**	K2M, A6T, V19L
**G5WKB9_ECOLX**	V19L, A22T
**F4TJV8_ECOLX**	V19L, A22T
**G1ZVT9_ECOLX**	V19L, A22T
**H5NFV4_ECOLX**	V19L, A22T
**E9Y7R9_ECOLX**	A6T, V19L
**H9YDF6_ECOKO**	V19L, A22T
**H4NJR3_ECOLX**	A6T, V19L, A21T
**H5KI64_ECOLX**	V19L, A22T
**E9U427_ECOLX**	V19L, A22T
**D5CWF7_ECOKI**	V19L, A22T
**F9S7G0_9VIBR**	A6T, V19L, A22T
**H4Q809_ECOLX**	V19L, A22T
**H5GVQ2_ECOLX**	V19L, A22T
**G0GSM6_KLEPN**	V19L, A22T
**H8D654_ECOLX**	V19L, A22T
**D8E1P5_ECOLX**	V19L, A22T
**H1DN41_ECOLX**	V19L, A22T
**F1XMB4_ECO57**	A6T, V19L, A21T

Supplementary Table S11A. Globular proteins used to compute the correlated mutation index.

PDB ID	***Chain ID***

**1a3a**	A
**1a6m**	A
**1a70**	A
**1aap**	A
**1aba**	A
**1ag6**	A
**1aoe**	A
**1atl**	A
**1atz**	A
**1avs**	A
**1bdo**	A
**1beb**	A
**1beh**	A
**1bkr**	A
**1brf**	A
**1bsg**	A
**1c44**	A
**1c52**	A
**1c9o**	A
**1cc8**	A
**1chd**	A
**1cjw**	A
**1cke**	A
**1ctf**	A
**1cxy**	A
**1czn**	A
**1d0q**	A
**1d1q**	A
**1d4o**	A
**1dbx**	A

**1dix**	A
**1dlw**	A
**1dmg**	A
**1dqg**	A
**1dsx**	A
**1eaz**	A
**1ej0**	A
**1ej8**	A
**1ek0**	A
**1f6b**	A
**1fcy**	A
**1fk5**	A
**1fl0**	A
**1fna**	A
**1fqt**	A
**1fvg**	A
**1fvk**	A
**1fx2**	A
**1g2r**	A
**1g9o**	A
**1gbs**	A
**1gmi**	A
**1gmx**	A
**1guu**	A
**1gz2**	A
**1gzc**	A
**1h0p**	A
**1h2e**	A
**1h4x**	A
**1h98**	A

**1hdo**	A
**1hfc**	A
**1hh8**	A
**1htw**	A
**1hxn**	A
**1i1j**	A
**1i1n**	A
**1i4j**	A
**1i58**	A
**1i5g**	A
**1i71**	A
**1ihz**	A
**1iib**	A
**1im5**	A
**1iwd**	A
**1j3a**	A
**1jbe**	A
**1jbk**	A
**1jfu**	A
**1jfx**	A
**1jkx**	A
**1jl1**	A
**1jo0**	A
**1jo8**	A
**1jos**	A
**1jvw**	A
**1jwq**	A
**1jyh**	A
**1k6k**	A
**1k7c**	A

**1k7j**	A
**1kid**	A
**1kq6**	A
**1kqr**	A
**1ktg**	A
**1ku3**	A
**1kw4**	A
**1lm4**	A
**1lo7**	A
**1lpy**	A
**1m4j**	A
**1m8a**	A
**1mk0**	A
**1mug**	A
**1nb9**	A
**1ne2**	A
**1nps**	A
**1nrv**	A
**1ny1**	A
**1o1z**	A
**1p90**	A
**1pch**	A
**1pko**	A
**1qf9**	A
**1qjp**	A
**1ql0**	A
**1r26**	A
**1roa**	A
**1rw1**	A
**1rw7**	A

**1ryb**	A
**1smx**	A
**1svy**	A
**1t8k**	A
**1tif**	A
**1tqg**	A
**1tqh**	A
**1tzv**	A
**1vfy**	A
**1vhu**	A
**1vjk**	A
**1vmb**	A
**1vp6**	A
**1w0h**	A
**1whi**	A
**1wjx**	A
**1wkc**	A
**1xdz**	A
**1xff**	A
**1xkr**	A
**2arc**	A
**2cua**	A
**2hs1**	A
**2mhr**	A
**2phy**	A
**2tps**	A
**2vxn**	A
**3bor**	A
**3dqg**	A
**5ptp**	A

Supplementary Table S11B. Transmembrane proteins used to compute the correlated mutation index.

PDB ID	***Chain ID***
**2fma**	A
**1lv7**	A
**1ihr**	A
**1l2p**	A
**1nkz**	R
**1rzh**	H
**2wjn**	H
**2cua**	A
**3s8f**	C
**3cx5**	O
**3cx5**	P
**3cx5**	S
**3cx5**	T
**1kqf**	H
**1nkz**	S
**1v54**	Q
**1v54**	T
**1v54**	V
**1v54**	W
**1v54**	X
**1v54**	Y
**1v54**	Z
**1s3e**	A
**1e2w**	A
**1a64**	A

**1fv1**	A
**1icf**	I
**1xiw**	A
**4tsv**	A
**1mf7**	A
**1xh3**	A
**2hft**	_
**1ijq**	A
**1aly**	_
**1v7p**	C
**1eue**	A
**1i4f**	A
**1b0x**	A
**1f2l**	A
**1b4f**	B
**1bqu**	A
**2ddf**	A
**1n7s**	B
**1bte**	A
**1tjx**	A
**1juf**	A
**1ql3**	A
**1cdy**	_
**1cfb**	_
**1eer**	C
**1mjn**	A
**1j8e**	A
**1hcz**	_
**1dg6**	A
**1d5m**	B

**1iak**	A
**1iak**	B
**1dl2**	A
**1r1h**	A
**1klu**	B
**1dp4**	A
**1dqg**	A
**1dqt**	A
**1e1h**	B
**1gp0**	A
**1e87**	A
**1eax**	A
**1edh**	A
**1epf**	A
**1rew**	D
**1g1t**	A
**1ext**	A
**1f46**	A
**1yro**	D
**2b8l**	A
**1flt**	X
**1x9d**	A
**1fne**	A
**1lk2**	A
**1g1s**	A
**1r2d**	A
**1gv9**	A
**1p9a**	G
**1o7q**	A
**2cl5**	A

**1he7**	A
**1jgd**	A
**1qwn**	A
**1vca**	A
**1iko**	P
**1s55**	A
**1jd0**	A
**1jdp**	A
**1jfu**	A
**1jln**	A
**1yuk**	B
**2bnu**	A
**1m9z**	A
**1lqv**	A
**1lar**	A
**1lcy**	A
**1lr0**	A
**1lzj**	A
**1m6o**	A
**1n3y**	A
**1ncg**	_
**1neu**	_
**1nko**	A
**1nkr**	_
**1npu**	A
**1nrj**	B
**1pfq**	A
**1z8g**	A
**1uze**	A
**1qfo**	A

**1olz**	A
**1wt4**	B
**1yfn**	E
**1ot8**	A
**1p15**	A
**2f8y**	B
**1pt6**	A
**1r55**	A
**1rfs**	_
**1rhf**	A
**1rm8**	A
**1shu**	X
**1t0p**	B
**1uv7**	A
**1v84**	A
**1w15**	A
**1wwc**	A
**1xed**	A
**1xiw**	F
**1xu1**	R
**1ydp**	A
**2o3s**	A
**1z40**	A
**1z5y**	E
**1z9l**	A
**2a6z**	A
**2fy7**	A
**2ajc**	A
**2b7k**	A
**2hc1**	A

**2c6c**	A
**2c7s**	A
**2fcb**	A
**2gu3**	A
**2tnf**	A
**2apc**	A
**2nvg**	A
**2j8c**	H
**2bba**	A
**2ciu**	A
**2d0j**	A
**2gak**	A
**2gsf**	A
**2h1b**	A
**2h26**	A
**2h4v**	A
**2hey**	R
**2hew**	F
**2hjl**	A
**2hlr**	A
**2i1m**	A
**2i6v**	A
**2ims**	A
**2jcq**	A
**2isg**	A
**3arc**	e
**3arc**	f
**3arc**	h
**3arc**	i
**3arc**	j

**3arc**	k
**3arc**	l
**3arc**	m
**3arc**	t
**3arc**	y
**3arc**	x
**2z62**	A
**3llk**	C
**4n7i**	A
**2qqi**	A
**2r2o**	B
**1fv1**	E
**2gh0**	B
**3sov**	A
**2omz**	B
**2haz**	A
**4kmy**	A
**4fyt**	A
**4km6**	A
**1sm3**	P
**1itu**	B
**2o6l**	B
**3s98**	A
**1ly2**	A
**4h2g**	A
**3jxf**	B
**3i84**	B
**3v6o**	B
**2pet**	A
**1ypq**	B

**2fh7**	A
**3teq**	D
**4cnm**	A
**2h62**	D
**1eg4**	P
**4gwm**	B
**3pv7**	A
**3kgr**	C
**4l4v**	C
**3f6k**	A
**4h5s**	B
**3n2n**	F
**3wn4**	A
**2jjw**	A
**4hdq**	C
**3fed**	A
**3h6n**	A
**3pxh**	A
**2y8t**	D
**1ynl**	H
**3nfk**	D
**2bnq**	E
**3pve**	B
**2d3v**	A
**4eby**	A
**2qsq**	B
**1h03**	Q
**4fke**	A
**3cl5**	A
**3qw9**	B

**2dur**	B
**3qup**	A
**3nk4**	D
**2wv3**	A
**2yz1**	B
**4fww**	A
**3t6q**	B
**3bix**	D
**3o71**	B
**2xot**	B
**2ox9**	D
**2ic2**	B
**4lxj**	A
**2a6v**	B
**2qqj**	A
**2v9t**	A
**2vsm**	B
**3r0n**	A
**2ffu**	A
**3n1f**	D
**1po5**	A
**3g5b**	A
**2j0a**	A
**2ooq**	B
**3noi**	B
**4oac**	A
**3ner**	B
**3l4y**	A
**4j2c**	C
**1hq8**	A

**4dnd**	A
**1oll**	A
**3ff9**	B
**1gl2**	C
**3q3j**	A
**4iqh**	C
**3pvh**	A
**4h5s**	A
**1cyo**	A
**2q86**	C
**1oga**	E
**2q86**	D
**2aju**	H
**3vcl**	A
**1uvq**	A
**1qjb**	S
**1lcj**	B
**2hi4**	A
**3rl1**	A
**3vj6**	A
**1j02**	A
**1n45**	B
**4a4y**	A
**3owv**	B
**3lgi**	C
**3azc**	A
**2rl8**	B
**3khq**	A
**2wy3**	D
**2yd1**	A

**4nn5**	B
**2bvp**	A
**3rrc**	B
**2h64**	C
**3m9z**	A
**3b5n**	I
**1xr9**	A
**4jqv**	A
**3ln4**	A
**4jqu**	B
**3fp2**	A
**3zf8**	A
**1tvb**	F
**4g1i**	B
**2ibj**	A
**4hq1**	A
**4nzr**	M
**3q2u**	A
**2wo1**	B
**2zvy**	B
**2jik**	B
**3cyp**	E
**4e5x**	H
**1eaj**	B
**3ig3**	A
**4bhu**	J
**1ci3**	M
**4dgj**	D
**3gmg**	B
**4esq**	A

**3c5j**	B
**1rwi**	B
**1zxk**	B
**3f61**	A
**3ca7**	A
**4alz**	A
**3onj**	A
**1obx**	B
**2oo4**	B
**2wnf**	A
**2ptt**	B
**2guz**	O
**4of8**	D
**3q4u**	D
**1wt6**	D
**3jxg**	D
**2z3q**	D
**4bkj**	B
**1jr8**	B
**3o3u**	N
**1d4t**	B
**3gyl**	B
**3ey6**	A
**2wy3**	C
**2rei**	A
**3q0h**	B
**4ggj**	A
**2oya**	B
**2bpd**	B
**3vpp**	B

**3so5**	B
**3mw4**	C
**3lz6**	D
**2q8o**	B
**4nmu**	D
**2avd**	B
**2frg**	P
**4esk**	D
**4de9**	A
**4gos**	A
**4oag**	B
**2f4w**	B
**2gw5**	A
**2c1i**	A
**1ugn**	A
**4mz2**	A
**2e7v**	A
**4m7h**	B
**3dlq**	R
**2b69**	A
**3p2t**	A
**3wbp**	B
**1s1d**	B
**2pof**	B
**3ff7**	D
**3mxo**	B
**3mvs**	A
**4apx**	B
**3n1m**	C
**2hl7**	A

**3vbc**	A
**3bod**	A
**4e74**	A
**4m92**	B
**1u9k**	B
**2xr6**	A
**1smo**	B
**2yhf**	I
**4ezf**	B
**4ak8**	D
**2c6u**	A
**4lu3**	A
**3wh2**	A
**2q87**	C
**2oq5**	A
**2yfx**	A
**2r32**	A
**3mdm**	A
**4gel**	B
**4lr2**	A
**3bp6**	B
**3rs1**	B
**1t8t**	B
**2icc**	A
**2wh6**	A
**2xz4**	B
**2o9g**	D
**3bs6**	B
**2fwh**	A
**1d06**	A

**2ff7**	A
**1b12**	A
**2cbz**	A
**1d4o**	A
**3llo**	A
**1a3a**	A
**2zpm**	A
**1vls**	_
**1rzh**	L
**2a65**	A
**1dsx**	D
**1rzh**	M
**1m0k**	C
**2ih3**	F
**2wjn**	L
**2wjn**	M
**2jaf**	C
**2bs2**	F
**3s8f**	A
**3cx5**	N
**3qap**	A
**1h2s**	D
**4dx5**	C
**3arc**	a
**3arc**	b
**3arc**	c
**3arc**	d
**1kqf**	I
**3ldc**	D
**2gsm**	A

**2gsm**	B
**4eiy**	A
**3bya**	B
**1v54**	N
**1v54**	O
**1v54**	P
**1ors**	C
**4n6h**	A
**1q16**	C
**2b6o**	A
**1u7g**	C
**1xio**	A
**2h88**	C
**2h88**	D
**2f2b**	D
**2b2h**	C
**3k04**	E
**2a0b**	_
**1gpr**	_
**1djl**	A
**1dqa**	A
**1mqi**	A
**1fx2**	A
**1fx4**	A
**1g4y**	B
**1id0**	A
**1ijy**	A
**1xzz**	A
**1nxc**	A
**1p0z**	A

**1pb7**	A
**1py9**	A
**1q3e**	A
**1s50**	A
**1t0j**	C
**2f34**	A
**1uc7**	A
**1xaw**	A
**1x13**	A
**1xkz**	A
**1ysr**	A
**2hje**	A
**2a5s**	A
**2xov**	A
**4a82**	B
**2j8c**	L
**2j8c**	M
**2bcx**	B
**2bou**	A
**2ixe**	A
**3i8s**	C
**3l9w**	B
**3bp3**	B
**3fwz**	B
**4n7w**	A
**3qbg**	D
**3arc**	z
**3pcv**	C
**3b9w**	C
**3m73**	C

**3d9s**	D
**3ddl**	A
**4al0**	C
**3gd8**	D
**2qts**	C
**3zoj**	D
**4f4s**	T
**2xqu**	P
**3v5u**	A
**3tds**	D
**3ouf**	E
**3rlb**	A
**4ikv**	A
**3gkj**	A
**4ms4**	B
**3a1f**	A
**3rn8**	C
**2vns**	B
**3w19**	C
**2y8t**	E
**3lsw**	A
**3s9e**	B
**1mix**	A
**3om0**	A
**3lwt**	X
**4m5b**	A
**4o6y**	B
**4etv**	B
**3a9f**	B
**2pux**	C

**2voh**	B
**3dt5**	A
**1z0w**	A
**2xme**	L
**3skx**	A
**2r48**	A
**3kkb**	B
**3ehg**	A
**3tyq**	B
**1aol**	A
**2x49**	A
**3iqt**	A
**4o6m**	B
**4pgr**	A
**3gwi**	A
**4hhx**	A
**3ezh**	B
**3pp4**	P
**4dey**	B
**1mvu**	P
**4bgc**	A
**4e29**	B
**3g3t**	A
**3hfe**	C
**2pfi**	B
**2cjz**	A
**3jrr**	B
**2jli**	A
**2r4q**	A
**2nl9**	A

**3k6y**	A
**3rkg**	A
**1txo**	B
**4dex**	B
**3pty**	A
**2xdg**	B
**3zlc**	B
**3kyz**	A
**3qxm**	B
**3hrn**	A
**3nfl**	H
**2vay**	B
**4erv**	A
**4djc**	B
**3ftj**	A
**3swy**	C
**4loj**	B
**3sl2**	A
**1xhk**	B
**3vn0**	D
**2wji**	B
**4gag**	P
**2v3u**	A
**1s6c**	B
**2w5q**	A
**4gt8**	A
**3eu9**	C
**4emt**	B
**3cqb**	B
**4n5q**	B

**1ia9**	B
**1wv3**	A
**2rca**	B
**2rfa**	A
**4ehq**	G
**3t4l**	B
**4aqr**	D
**2rc8**	B
**2xtp**	A
**3dxs**	X
**4pas**	A
**2f37**	B
**2etb**	A
**1qjp**	A
**3m4d**	G
**3prn**	_
**4gcs**	A
**2fgq**	B
**2vdf**	A
**1kmo**	A
**3gp6**	A
**2guf**	A
**1qj8**	A
**2por**	_
**3pgu**	A
**1yc9**	C
**2erv**	B
**1xkw**	A
**2gr8**	D
**2o4v**	C

**2j1n**	C
**2y2x**	A
**3aeh**	A
**2zfg**	C
**3fid**	A
**4afk**	A
**2wjr**	A
**2x55**	A
**4fqe**	A
**3szv**	A
**4e1s**	A
**3qra**	A
**4frx**	A
**1f00**	I

Supplementary Table S12A. Globular proteins used to compute maximal cliques, size-order and spherical coordinates.
16PK A,1A0M A,1A0M B,1A12 A,1A12 B,1A12 C,1A1X A,1A27 A,1A2P A,1A2P B,1A2P C,1A3A A,1A3A B,1A3A C,1A3A D,1A3I A,1A3I B,1A3I C,1A3J A,1A3J B,1A3J C,1A4I A,1A4I B,1A62 A,1A6M A,1A6Q A,1A7S A,1A7T A,1A7T B,1A7Y A,1A7Y B,1A7Y C,1A7Z A,1A7Z B,1A8D A,1A8L A,1A8Q A,1A92 A,1A92 B,1A92 C,1A92 D,1A9X A,1A9X B,1A9X C,1A9X D,1A9X E,1A9X F,1A9X G,1A9X H,1AA5 A,1AA5 B,1AAP A,1AAP B,1ABA A,1ACF A,1AE9 A,1AE9 B,1AF7 A,1AG9 A,1AG9 B,1AGJ A,1AGJ B,1AGQ A,1AGQ B,1AGQ C,1AGQ D,1AH7 A,1AHO A,1AIE A,1AJ2 A,1AJ8 A,1AJ8 B,1AJJ A,1AJK A,1AJK B,1AK0 A,1AKG A,1AKO A,1AL3 A,1AL4 A,1AL4 B,1ALQ A,1ALU A,1ALX A,1ALX B,1ALY A,1ALZ A,1ALZ B,1AMT A,1AMT B,1AMT C,1AMU A,1AMU B,1AMX A,1AOC A,1AOC B,1AOE A,1AOE B,1AOH A,1AOH B,1AOL A,1AOZ A,1AOZ B,1APY A,1APY B,1APY C,1APY D,1AQZ A,1AQZ B,1AT0 A,1ATG A,1ATZ A,1ATZ B,1AUO A,1AUO B,1AV2 A,1AV2 B,1AV2 C,1AV2 D,1AVY A,1AVY B,1AVY C,1AYE A,1AYO A,1AYO B,1AZO A,1B0B A,1B0N A,1B0N B,1B0U A,1B0X A,1B12 A,1B12 B,1B12 C,1B12 D,1B1C A,1B25 A,1B25 B,1B25 C,1B25 D,1B2P A,1B2P B,1B4F A,1B4F B,1B4F C,1B4F D,1B4F E,1B4F F,1B4F G,1B4F H,1B5E A,1B5E B,1B5F A,1B5F B,1B5F C,1B5F D,1B5P A,1B5P B,1B5Q A,1B5Q B,1B5Q C,1B63 A,1B65 A,1B65 B,1B65 C,1B65 D,1B65 E,1B65 F,1B67 A,1B67 B,1B6A A,1B6G A,1B8A A,1B8A B,1B93 A,1B93 B,1B93 C,1B9H A,1B9M A,1B9M B,1B9O A,1B9W A,1BAM A,1BAZ A,1BAZ B,1BAZ C,1BAZ D,1BD3 A,1BD3 B,1BD3 C,1BD3 D,1BD8 A,1BDO A,1BDW A,1BDW B,1BEA A,1BF2 A,1BF6 A,1BF6 B,1BG6

A,1BGC A,1BGF A,1BGP A,1BGV A,1BHD A,1BHD B,1BHE A,1BI5 A,1BIF A,1BJ7 A,1BKB A,1BKP A,1BKP B,1BKR A,1BM8 A,1BM9 A,1BM9 B,1BOL A,1BQB A,1BQC A,1BQU A,1BQU B,1BRT A,1BS0 A,1BSL A,1BSL B,1BTE A,1BTE B,1BTK A,1BTK B,1BTN A,1BU8 A,1BUO A,1BUU A,1BX4 A,1BX7 A,1BXY A,1BXY B,1BY2 A,1BYF A,1BYF B,1BYI A,1BYR A,1BYZ A,1BYZ B,1BYZ C,1BYZ D,1BZ4 A,1C0P A,1C0Q A,1C0Q B,1C0R A,1C0R B,1C1K A,1C3C A,1C3C B,1C3M A,1C3P A,1C44 A,1C4D A,1C4D B,1C4D C,1C4D D,1C4O A,1C4Q A,1C4Q B,1C4Q C,1C4Q D,1C4Q E,1C52 A,1C53 A,1C5E A,1C5E B,1C5E C,1C75 A,1C7K A,1C7N A,1C7N B,1C7N C,1C7N D,1C7N E,1C7N F,1C7N G,1C7N H,1C8U A,1C8U B,1C9O A,1C9O B,1CB0 A,1CB8 A,1CC8 A,1CCW A,1CCW B,1CCW C,1CCW D,1CCZ A,1CDC A,1CDC B,1CEO A,1CEW I,1CFB A,1CG5 A,1CG5 B,1CHD A,1CHM A,1CHM B,1CI4 A,1CI4 B,1CI9 A,1CI9 B,1CJC A,1CJW A,1CKA A,1CKA B,1CKE A,1CMC A,1CMC B,1CNV A,1COJ A,1COZ A,1COZ B,1CPN A,1CPQ A,1CQ3 A,1CQ3 B,1CQ4 A,1CQ4 B,1CQM A,1CQM B,1CQX A,1CQX B,1CQY A,1CRU A,1CRU B,1CS1 A,1CS1 B,1CS1 C,1CS1 D,1CS6 A,1CS8 A,1CSB A,1CSB B,1CSB D,1CSB E,1CSH A,1CT5 A,1CT9 A,1CT9 B,1CT9 C,1CT9 D,1CTF A,1CUK A,1CUN A,1CUN B,1CUN C,1CV8 A,1CVR A,1CXC A,1CXQ A,1CXY A,1CY5 A,1CY9 A,1CY9 B,1CYD A,1CYD B,1CYD C,1CYD D,1CZA N,1CZF A,1CZF B,1CZQ A,1CZQ D,1CZT A,1CZY A,1CZY B,1CZY C,1CZY D,1CZY E,1D0Q A,1D0Q B,1D1Q A,1D1Q B,1D2N A,1D2O A,1D2O B,1D2S A,1D2T A,1D2Z A,1D2Z B,1D2Z C,1D2Z D,1D3B A,1D3B B,1D3B C,1D3B D,1D3B E,1D3B F,1D3B G,1D3B H,1D3B I,1D3B J,1D3B K,1D3B L,1D3Y A,1D3Y B,1D4A A,1D4A B,1D4A C,1D4A D,1D4O A,1D4T A,1D4T B,1D5T A,1D8H A,1D8H B,1D8H C,1D8W A,1D8W B,1D8W C,1D8W D,1D9C A,1D9C B,1DBF A,1DBF B,1DBF C,1DBW A,1DBW B,1DCI A,1DCI B,1DCI C,1DCS A,1DD9 A,1DDJ A,1DDJ B,1DDJ C,1DDJ D,1DDW A,1DEK A,1DEK B,1DEU A,1DEU B,1DF4 A,1DFA A,1DG6 A,1DGF A,1DGF B,1DGF C,1DGF D,1DGW A,1DGW X,1DGW Y,1DHN A,1DI6 A,1DJ0 A,1DJ0 B,1DJ8 A,1DJ8 B,1DJ8 C,1DJ8 D,1DJ8 E,1DJ8 F,1DJL A,1DJL B,1DK8 A,1DKI A,1DKI B,1DKI C,1DKI D,1DL2 A,1DL5 A,1DL5 B,1DLW A,1DLY A,1DM9 A,1DM9 B,1DMG A,1DMH A,1DMH B,1DNL A,1DNU A,1DNU B,1DNU C,1DNU D,1DOI A,1DOS A,1DOS B,1DOW A,1DOW B,1DP4 A,1DP4 C,1DPG A,1DPG B,1DPJ A,1DPJ B,1DQG A,1DQI A,1DQI B,1DQI C,1DQI D,1DQN A,1DQN B,1DQS A,1DQS B,1DS1 A,1DSL A,1DTJ A,1DTJ B,1DTJ C,1DTJ D,1DUN A,1DUS A,1DVO A,1DW0 A,1DW0 B,1DW0 C,1DWK A,1DWK B,1DWK C,1DWK D,1DWK E,1DWK F,1DWK G,1DWK H,1DWK I,1DWK J,1DXE A,1DXE B,1DXG A,1DXG B,1DXY A,1DY5 A,1DY5 B,1DYK A,1DYP A,1DYQ A,1DYS A,1DYS B,1DZ3 A,1DZF A,1DZK A,1DZK B,1E0B A,1E0B B,1E0C A,1E0T A,1E0T B,1E0T C,1E0T D,1E19 A,1E19 B,1E1H A,1E1H B,1E1H C,1E1H D,1E25 A,1E29 A,1E2K A,1E2K B,1E2W A,1E2W B,1E4C P,1E4M M,1E58 A,1E5K A,1E5P A,1E5P B,1E5P C,1E5P D,1E6B A,1E6C A,1E6C B,1E6I A,1E6I P,1E6U A,1E7L A,1E7L B,1E7W A,1E7W B,1E8C A,1E8C B,1E8Y A,1E9G A,1E9G B,1E9W A,1EA5 A,1EAJ A,1EAJ B,1EAQ A,1EAQ B,1EAR A,1EAZ A,1EB6 A,1ECA A,1ECF A,1ECF B,1ECS A,1ECS B,1EDG A,1EDM B,1EDM C,1EDT A,1EE8 A,1EE8 B,1EER A,1EER B,1EER C,1EEX A,1EEX B,1EEX E,1EEX G,1EEX L,1EEX M,1EF8 A,1EF8 B,1EF8 C,1EFD N,1EG2 A,1EG3 A,1EG5 A,1EG5 B,1EGP A,1EGP B,1EH6 A,1EI5 A,1EJ0 A,1EJ2 A,1EJ8 A,1EJB A,1EJB B,1EJB C,1EJB D,1EJB E,1EJD A,1EJD B,1EJG A,1EK6 A,1EK6 B,1EKE A,1EKE B,1EKJ A,1EKJ B,1EKJ C,1EKJ D,1EKJ E,1EKJ F,1EKJ G,1EKJ H,1EKQ

A,1EKQ B,1EL6 A,1EL6 B,1EL6 C,1ELJ A,1ELK A,1ELK B,1ELU A,1ELU B,1ELV A,1ELW A,1ELW B,1ELW C,1ELW D,1EMU A,1EMU B,1EN2 A,1ENF A,1EOK A,1EP0 A,1EPW A,1EQ2 A,1EQ2 B,1EQ2 C,1EQ2 D,1EQ2 E,1EQ2 F,1EQ2 G,1EQ2 H,1EQ2 I,1EQ2 J,1EQ9 A,1EQ9 B,1ES5 A,1ES9 A,1ESW A,1ET1 A,1ET1 B,1ET9 A,1ETL A,1ETM A,1ETN A,1EU1 A,1EU3 A,1EU3 B,1EU8 A,1EUH A,1EUH B,1EUH C,1EUH D,1EUW A,1EVL A,1EVL B,1EVL C,1EVL D,1EVX A,1EVX B,1EVY A,1EW0 A,1EW4 A,1EWF A,1EX2 A,1EX2 B,1EX7 A,1EXR A,1EXT A,1EXT B,1EYE A,1EYH A,1EYQ A,1EYQ B,1EYV A,1EYV B,1EZ3 A,1EZ3 B,1EZ3 C,1EZG A,1EZG B,1EZI A,1EZI B,1EZJ A,1EZW A,1F00 I,1F08 A,1F08 B,1F0I A,1F0K A,1F0K B,1F0L A,1F0L B,1F0X A,1F0X B,1F0Y A,1F0Y B,1F1E A,1F1M A,1F1M B,1F1M C,1F1M D,1F1U A,1F1U B,1F20 A,1F2L A,1F2L B,1F2L C,1F2L D,1F2T A,1F2T B,1F32 A,1F39 A,1F39 B,1F3U A,1F3U B,1F3U C,1F3U D,1F3U E,1F3U F,1F3U G,1F3U H,1F3Z A,1F46 A,1F46 B,1F4P A,1F5M A,1F5M B,1F5N A,1F5V A,1F5V B,1F60 A,1F60 B,1F6B A,1F6B B,1F74 A,1F74 C,1F7D A,1F7D B,1F7L A,1F7S A,1F86 A,1F86 B,1F94 A,1F9P A,1F9V A,1F9Z A,1F9Z B,1FAO A,1FBT A,1FBT B,1FC3 A,1FC3 B,1FC3 C,1FC4 A,1FC4 B,1FC6 A,1FCQ A,1FCY A,1FD3 A,1FD3 B,1FD3 C,1FD3 D,1FEC A,1FEC B,1FG7 A,1FGY A,1FHG A,1FIP A,1FIP B,1FIP C,1FIP D,1FIT A,1FJ2 A,1FJ2 B,1FJH A,1FJH B,1FJJ A,1FK5 A,1FKM A,1FL0 A,1FL2 A,1FM0 D,1FM0 E,1FMB A,1FMC A,1FMC B,1FMJ A,1FMJ B,1FMK A,1FN9 A,1FN9 B,1FNF A,1FNN A,1FNN B,1FO3 A,1FO8 A,1FOB A,1FP1 D,1FP2 A,1FP3 A,1FP3 B,1FPO A,1FPO B,1FPO C,1FPZ A,1FPZ B,1FPZ C,1FPZ D,1FPZ E,1FPZ F,1FQI A,1FR3 A,1FR3 B,1FR3 C,1FR3 D,1FR3 E,1FR3 F,1FR3 G,1FR3 H,1FR3 I,1FR3 J,1FR3 K,1FR3 L,1FS5 A,1FS5 B,1FSG A,1FSG C,1FT5 A,1FTH A,1FTH B,1FTH C,1FTR A,1FTR B,1FTR C,1FTR D,1FUK A,1FVG A,1FVI A,1FVM A,1FVM B,1FVM C,1FVM D,1FVM E,1FVM F,1FVM G,1FVM H,1FVM I,1FVM J,1FVM K,1FVM L,1FX2 A,1FXD A,1FXO A,1FXO B,1FXO C,1FXO D,1FXO E,1FXO F,1FXO G,1FXO H,1FYE A,1FZQ A,1FZV A,1FZV B,1G0C A,1G0S A,1G0S B,1G12 A,1G1J A,1G1J B,1G1T A,1G29 1,1G29 2,1G2B A,1G2N A,1G2O A,1G2O B,1G2O C,1G2Q A,1G2Q B,1G2R A,1G2Y A,1G2Y B,1G2Y C,1G2Y D,1G33 A,1G3K A,1G3K B,1G3K C,1G3P A,1G3Q A,1G4I A,1G55 A,1G57 A,1G57 B,1G5A A,1G5H A,1G5H B,1G5H C,1G5H D,1G5T A,1G60 A,1G60 B,1G61 A,1G61 B,1G66 A,1G6G A,1G6G B,1G6G E,1G6G F,1G6H A,1G6U A,1G6U B,1G6X A,1G7S A,1G87 A,1G87 B,1G8A A,1G8E A,1G8E B,1G8F A,1G8K A,1G8K B,1G8K C,1G8K D,1G8K E,1G8K F,1G8K G,1G8K H,1G8L A,1G8L B,1G8M A,1G8M B,1G8Q A,1G8Q B,1G94 A,1G9W A,1G9W B,1G9W C,1GA6 A,1GA6 I,1GA8 A,1GAI A,1GAK A,1GCI A,1GCY A,1GDE A,1GDE B,1GEE A,1GEE B,1GEE E,1GEE F,1GEF A,1GEF B,1GEF D,1GEF E,1GEQ A,1GEQ B,1GHE A,1GHE B,1GHG A,1GHG B,1GHG C,1GHG D,1GHP A,1GK6 A,1GK6 B,1GK7 A,1GK9 A,1GK9 B,1GKM A,1GKP A,1GKP B,1GKP C,1GKP D,1GKP E,1GKP F,1GL2 A,1GL2 B,1GL2 C,1GL2 D,1GL4 A,1GL4 B,1GMI A,1GMU A,1GMU B,1GMU C,1GMU D,1GMX A,1GNL A,1GNL B,1GNY A,1GO3 E,1GO3 F,1GO3 M,1GO3 N,1GO6 A,1GO6 B,1GO6 C,1GO6 D,1GO6 E,1GO6 F,1GO6 G,1GO6 H,1GO6 I,1GO6 K,1GO6 M,1GO6 O,1GOI A,1GOI B,1GP0 A,1GP1 A,1GP1 B,1GP6 A,1GPJ A,1GPP A,1GPR A,1GQ6 A,1GQ6 B,1GQ6 C,1GQ8 A,1GQA A,1GQA D,1GQE A,1GQV A,1GR0 A,1GR3 A,1GS5 A,1GS9 A,1GSA A,1GTE A,1GTE B,1GTE C,1GTE D,1GTK A,1GTT A,1GTT B,1GTT C,1GTT D,1GTV A,1GTV B,1GTZ A,1GTZ B,1GTZ C,1GTZ D,1GTZ E,1GTZ F,1GTZ G,1GTZ H,1GTZ I,1GTZ J,1GTZ K,1GTZ L,1GU2

A,1GU2 B,1GU7 A,1GU7 B,1GUD A,1GUD B,1GUI A,1GUQ A,1GUQ B,1GUQ C,1GUQ D,1GUT A,1GUT B,1GUT C,1GUT D,1GUT E,1GUT F,1GUX A,1GUX B,1GUX E,1GV2 A,1GV3 A,1GV3 B,1GV8 A,1GV9 A,1GVD A,1GVE A,1GVE B,1GVF A,1GVF B,1GVJ A,1GVJ B,1GVN A,1GVN B,1GVN C,1GVN D,1GVP A,1GVZ A,1GWE A,1GWM A,1GWU A,1GXJ A,1GXJ B,1GXM A,1GXM B,1GXQ A,1GXR A,1GXR B,1GXU A,1GXY A,1GXY B,1GY6 A,1GY6 B,1GY7 A,1GY7 B,1GY7 C,1GY7 D,1GY8 A,1GY8 B,1GY8 C,1GY8 D,1GYH A,1GYH B,1GYH C,1GYH D,1GYH E,1GYH F,1GYO A,1GYO B,1GYU A,1GYX A,1GYX B,1GZ2 A,1H03 P,1H03 Q,1H0B A,1H0B B,1H0H A,1H0H B,1H0H K,1H0H L,1H12 A,1H16 A,1H1N A,1H1N B,1H2B A,1H2B B,1H2E A,1H32 A,1H32 B,1H3F A,1H3F B,1H41 A,1H41 B,1H4A X,1H4X A,1H4X B,1H5Q A,1H5Q B,1H5Q C,1H5Q D,1H5Q E,1H5Q F,1H5Q G,1H5Q H,1H5Q I,1H5Q J,1H5Q K,1H5Q L,1H6H A,1H6K A,1H6K B,1H6K C,1H6K X,1H6K Y,1H6K Z,1H6T A,1H6U A,1H6W A,1H6W B,1H70 A,1H72 C,1H75 A,1H7C A,1H7E A,1H7E B,1H7S A,1H7S B,1H80 A,1H80 B,1H8P A,1H8P B,1H8U A,1H8U B,1H97 A,1H97 B,1H98 A,1H99 A,1H9M A,1H9M B,1HBK A,1HBN A,1HBN B,1HBN C,1HBN D,1HBN E,1HBN F,1HDH A,1HDH B,1HDK A,1HDO A,1HE7 A,1HFE L,1HFE M,1HFE S,1HFE T,1HFO A,1HFO B,1HFO C,1HFO D,1HFO E,1HFO F,1HG8 A,1HGX A,1HGX B,1HH3 A,1HH3 B,1HH3 C,1HH3 D,1HH8 A,1HHA A,1HHA B,1HHA C,1HHA D,1HHC A,1HHC B,1HHC C,1HHC D,1HHF A,1HHF B,1HHF C,1HHF D,1HHS A,1HHS B,1HHS C,1HHU A,1HHU B,1HHU C,1HHU D,1HHY A,1HHY B,1HHZ A,1HHZ B,1HHZ C,1HHZ D,1HHZ E,1HHZ F,1HJE A,1HLE A,1HLE B,1HLQ A,1HLQ B,1HLQ C,1HM9 A,1HM9 B,1HML A,1HN0 A,1HNJ A,1HO1 A,1HO1 B,1HO1 C,1HO1 D,1HP1 A,1HPC A,1HPC B,1HPI A,1HQ0 A,1HQJ A,1HQJ B,1HQJ C,1HQJ D,1HQJ E,1HQJ F,1HQJ G,1HQJ H,1HQJ I,1HQJ J,1HQJ K,1HQJ L,1HQS A,1HQS B,1HRU A,1HRU B,1HT6 A,1HTW A,1HTW B,1HTW C,1HUF A,1HUW A,1HW1 A,1HW1 B,1HX6 A,1HX6 B,1HX6 C,1HXH A,1HXH B,1HXH C,1HXH D,1HXI A,1HXN A,1HXR A,1HXR B,1HYE A,1HYO A,1HYO B,1HYP A,1HZ4 A,1HZ6 A,1HZ6 B,1HZ6 C,1HZT A,1I07 A,1I07 B,1I0R A,1I0R B,1I0V A,1I12 A,1I12 B,1I12 C,1I12 D,1I1J A,1I1J B,1I1N A,1I1W A,1I24 A,1I27 A,1I2H A,1I2K A,1I2T A,1I36 A,1I36 B,1I39 A,1I3C A,1I3C B,1I4J A,1I4J B,1I4U A,1I4U B,1I52 A,1I58 A,1I58 B,1I60 A,1I71 A,1I7K A,1I7K B,1I7N A,1I7N B,1I7Q A,1I7Q B,1I7Q C,1I7Q D,1I8A A,1I8D A,1I8D B,1I8D C,1I8F A,1I8F B,1I8F C,1I8F D,1I8F E,1I8F F,1I8F G,1I8O A,1I9G A,1I9S A,1I9Z A,1IA6 A,1IA9 A,1IA9 B,1IAE A,1IAP A,1IBY A,1IBY B,1IBY C,1IBY D,1ID0 A,1IDP A,1IDP B,1IDP C,1IFC A,1IFR A,1IG0 A,1IG0 B,1IGQ A,1IGQ B,1IGQ C,1IGQ D,1IHB A,1IHB B,1IHG A,1IHJ A,1IHJ B,1IHJ C,1IHJ D,1IHR A,1IHR B,1II2 A,1II2 B,1II5 A,1IIB A,1IIB B,1IJB A,1IJQ A,1IJQ B,1IJT A,1IJX A,1IJX B,1IJX C,1IJX D,1IJX E,1IJX F,1IJY A,1IJY B,1IK6 A,1IKO P,1IKP A,1IKT A,1ILK A,1ILT A,1ILT B,1IM5 A,1IN4 A,1INL A,1INL B,1INL C,1INL D,1IO0 A,1IO1 A,1IO2 A,1IO7 A,1IO7 B,1IOM A,1IOO A,1IOO B,1IPC A,1IQ4 A,1IQ4 B,1IQ6 A,1IQ6 B,1IQQ A,1IQZ A,1IRQ A,1IRQ B,1IS3 A,1ISP A,1ISU A,1ISU B,1IT2 A,1IT2 B,1ITT A,1ITT B,1ITT C,1ITU A,1ITU B,1ITV A,1ITV B,1ITX A,1IU1 A,1IU1 B,1IU8 A,1IU8 B,1IUJ A,1IUJ B,1IUK A,1IUQ A,1IV2 A,1IV2 B,1IV2 C,1IV2 D,1IV2 E,1IV2 F,1IX9 A,1IX9 B,1IXH A,1IXK A,1IXL A,1IY8 A,1IY8 B,1IY8 C,1IY8 D,1IY8 E,1IY8 F,1IY8 G,1IY8 H,1IYB A,1IYB B,1IYE A,1IYE B,1IYE C,1IYN A,1IZC A,1IZM A,1J09 A,1J0H A,1J0H B,1J0P A,1J1B A,1J1B B,1J1I A,1J1T A,1J24 A,1J27 A,1J2R A,1J2R B,1J2R C,1J2R D,1J30 A,1J30 B,1J34 A,1J34 B,1J34 C,1J3A A,1J3B A,1J3B B,1J3M A,1J3M B,1J3W A,1J3W B,1J3W C,1J3W D,1J48 A,1J48 B,1J4A

A,1J4A B,1J4A C,1J4A D,1J5P A,1J5U A,1J5W A,1J5W B,1J5X A,1J6O A,1J77 A,1J7X A,1J83 A,1J83 B,1J8B A,1J8E A,1J8M F,1J8R A,1J8U A,1J97 A,1J97 B,1J98 A,1J99 A,1J9B A,1J9L A,1J9L B,1JAK A,1JAY A,1JAY B,1JB3 A,1JBE A,1JBW A,1JC4 A,1JC4 B,1JC4 C,1JC4 D,1JCD A,1JCD B,1JCD C,1JCL A,1JCL B,1JD1 A,1JD1 B,1JD1 C,1JD1 D,1JD1 E,1JD1 F,1JD5 A,1JD5 B,1JDC A,1JDP A,1JDP B,1JDP H,1JDW A,1JE0 A,1JE0 B,1JE0 C,1JE5 A,1JE5 B,1JEK A,1JEK B,1JEO A,1JER A,1JET A,1JET B,1JF8 A,1JFB A,1JFL A,1JFL B,1JFU A,1JFU B,1JFX A,1JG1 A,1JGT A,1JGT B,1JH6 A,1JH6 B,1JHC A,1JHF A,1JHF B,1JHG A,1JHJ A,1JHS A,1JI0 A,1JI1 A,1JI1 B,1JI7 A,1JI7 B,1JI7 C,1JIG A,1JIG B,1JIG C,1JIG D,1JIX A,1JJF A,1JJV A,1JKE A,1JKE B,1JKE C,1JKE D,1JKG A,1JKG B,1JKM A,1JKM B,1JKX A,1JKX B,1JKX C,1JKX D,1JL0 A,1JL0 B,1JLJ A,1JLJ B,1JLJ C,1JM0 A,1JM0 B,1JM0 C,1JM0 D,1JM0 E,1JM0 F,1JM1 A,1JMK C,1JMK O,1JMV A,1JMV B,1JMV C,1JMV D,1JMW A,1JND A,1JNI A,1JNR A,1JNR B,1JNR C,1JNR D,1JO0 A,1JO0 B,1JO8 A,1JOH A,1JOH B,1JOS A,1JOV A,1JP4 A,1JPE A,1JQ0 A,1JQ5 A,1JQE A,1JQE B,1JR2 A,1JR2 B,1JR7 A,1JR8 A,1JR8 B,1JRL A,1JS1 X,1JS1 Y,1JS1 Z,1JTV A,1JU2 A,1JU2 B,1JUH A,1JUH B,1JUH C,1JUH D,1JUV A,1JV1 A,1JV1 B,1JVB A,1JVW A,1JWQ A,1JX6 A,1JY1 A,1JY2 N,1JY2 O,1JY2 P,1JY2 Q,1JY2 R,1JY2 S,1JYA A,1JYA B,1JYE A,1JYH A,1JYK A,1JYO A,1JYO B,1JYO C,1JYO D,1JYO E,1JYO F,1JZ8 A,1JZ8 B,1JZ8 C,1JZ8 D,1JZT A,1JZT B,1K04 A,1K07 A,1K07 B,1K0E A,1K0E B,1K0R A,1K0R B,1K12 A,1K1B A,1K1E A,1K1E B,1K1E C,1K1E D,1K1E E,1K1E F,1K1E G,1K1E H,1K1E I,1K1E J,1K1E K,1K1E L,1K20 A,1K20 B,1K2E A,1K2E B,1K2X A,1K2X B,1K2X C,1K2X D,1K32 A,1K32 B,1K32 C,1K32 D,1K32 E,1K32 F,1K38 A,1K38 B,1K3I A,1K3S A,1K3S B,1K3Y A,1K3Y B,1K4I A,1K4M A,1K4M B,1K4M C,1K4N A,1K5C A,1K5N A,1K5N B,1K5N C,1K66 A,1K66 B,1K6D A,1K6D B,1K6K A,1K75 A,1K75 B,1K77 A,1K7C A,1K7H A,1K7H B,1K7J A,1K7K A,1K8K A,1K8K B,1K8K C,1K8K D,1K8K E,1K8K F,1K8K G,1K92 A,1K94 A,1K94 B,1K9U A,1K9U B,1KA1 A,1KAF A,1KAF B,1KAF C,1KAF D,1KAF E,1KAF F,1KB0 A,1KBL A,1KBV A,1KBV B,1KBV C,1KBV D,1KBV E,1KBV F,1KCM A,1KCQ A,1KDG A,1KDG B,1KEA A,1KEW A,1KEW B,1KEX A,1KFW A,1KGD A,1KGS A,1KHC A,1KHD A,1KHD B,1KHD C,1KHD D,1KHI A,1KHX A,1KHY A,1KHY B,1KHY C,1KHY D,1KI0 A,1KJQ A,1KJQ B,1KKO A,1KKO B,1KL9 A,1KLL A,1KLX A,1KM8 A,1KMJ A,1KMO A,1KMT A,1KMT B,1KMV A,1KNC A,1KNC B,1KNC C,1KNG A,1KNM A,1KNQ A,1KNQ B,1KO7 A,1KO7 B,1KOE A,1KOL A,1KOL B,1KOP A,1KOP B,1KP6 A,1KP8 A,1KP8 B,1KP8 C,1KP8 D,1KP8 E,1KP8 F,1KP8 G,1KP8 H,1KP8 I,1KP8 J,1KP8 K,1KP8 L,1KP8 M,1KP8 N,1KPG A,1KPG B,1KPG C,1KPG D,1KPT A,1KPT B,1KQ1 A,1KQ1 B,1KQ1 H,1KQ1 I,1KQ1 K,1KQ1 M,1KQ1 N,1KQ1 R,1KQ1 S,1KQ1 T,1KQ1 W,1KQ1 Y,1KQ3 A,1KQ6 A,1KQF A,1KQF B,1KQF C,1KQP A,1KQP B,1KQR A,1KR4 A,1KRH A,1KRH B,1KS8 A,1KS9 A,1KT6 A,1KTG A,1KTG B,1KU1 A,1KU1 B,1KU3 A,1KUX A,1KV9 A,1KVE A,1KVE B,1KVE C,1KVE D,1KW3 B,1KW4 A,1KWA A,1KWA B,1KWF A,1KWG A,1KWM A,1KWM B,1KWW A,1KWW B,1KWW C,1KXG A,1KXG B,1KXG C,1KXG D,1KXG E,1KXG F,1KXO A,1KYC A,1KYF A,1KYF P,1KZF A,1KZQ A,1KZQ B,1L1L A,1L1L B,1L1L C,1L1L D,1L1Q A,1L2H A,1L2P A,1L3I A,1L3I B,1L3I C,1L3I D,1L3I E,1L3I F,1L3K A,1L3P A,1L4X A,1L4X B,1L4X C,1L4X D,1L4X E,1L4X F,1L4X G,1L4X H,1L5W A,1L5W B,1L5X A,1L5X B,1L6P A,1L6R A,1L6R B,1L6S A,1L6S B,1L6W A,1L6W B,1L6W C,1L6W D,1L6W E,1L6W F,1L6W G,1L6W H,1L6W I,1L6W J,1L6X

A,1L6X B,1L7A A,1L7A B,1L7D A,1L7D B,1L7D C,1L7D D,1L8N A,1L8R A,1L8R B,1L9L A,1L9X A,1L9X B,1L9X C,1L9X D,1LAM A,1LB6 A,1LB6 B,1LBU A,1LBV A,1LBV B,1LC0 A,1LC5 A,1LCF A,1LCY A,1LDD A,1LDD B,1LDD C,1LDD D,1LE6 A,1LE6 B,1LE6 C,1LED A,1LF7 A,1LFK A,1LFP A,1LFW A,1LG7 A,1LGP A,1LH0 A,1LH0 B,1LJ5 A,1LJ8 A,1LJ9 A,1LJ9 B,1LJO A,1LKI A,1LKK A,1LKK B,1LKP A,1LL2 A,1LLD A,1LLD B,1LLF A,1LLF B,1LLN A,1LM5 A,1LM5 B,1LMI A,1LML A,1LNI A,1LNI B,1LO6 A,1LO7 A,1LQ9 A,1LQ9 B,1LQA A,1LQA B,1LQT A,1LQT B,1LQV A,1LQV B,1LQV C,1LQV D,1LR0 A,1LR5 A,1LR5 B,1LR5 C,1LR5 D,1LR7 A,1LS1 A,1LSH A,1LSH B,1LSL A,1LST A,1LU0 A,1LU0 B,1LU4 A,1LUA A,1LUA B,1LUA C,1LUR A,1LUR B,1LUZ A,1LUZ B,1LV7 A,1LWB A,1LXI A,1LXJ A,1LY1 A,1LY2 A,1LYQ A,1LYQ B,1LYV A,1LZL A,1M0D A,1M0D B,1M0D C,1M0D D,1M0K A,1M0U A,1M0U B,1M0W A,1M0W B,1M15 A,1M1F A,1M1F B,1M1H A,1M1Q A,1M1S A,1M22 A,1M22 B,1M24 A,1M24 B,1M2D A,1M2D B,1M2K A,1M2X A,1M2X B,1M2X C,1M2X D,1M3S A,1M3S B,1M3U A,1M3U B,1M3U C,1M3U D,1M3U E,1M3U F,1M3U G,1M3U H,1M3U I,1M3U J,1M3Y A,1M3Y B,1M3Y C,1M3Y D,1M40 A,1M45 A,1M45 B,1M48 A,1M48 B,1M4I A,1M4I B,1M4L A,1M55 A,1M55 B,1M5I A,1M5Q 1,1M5Q 2,1M5Q A,1M5Q B,1M5Q C,1M5Q D,1M5Q E,1M5Q F,1M5Q G,1M5Q H,1M5Q I,1M5Q J,1M5Q K,1M5Q L,1M5Q M,1M5Q N,1M5Q O,1M5Q P,1M5Q Q,1M5Q R,1M5Q S,1M5Q T,1M5Q U,1M5Q V,1M5Q W,1M5Q X,1M5Q Y,1M5Q Z,1M65 A,1M6D A,1M6D B,1M6S A,1M6S B,1M6S C,1M6S D,1M6Y A,1M6Y B,1M70 A,1M70 B,1M70 C,1M70 D,1M8A A,1M8A B,1M93 A,1M93 B,1M93 C,1M9Z A,1MAI A,1MB3 A,1MBA A,1MBM A,1MBM B,1MBM C,1MBM D,1MBY A,1MBY B,1MC2 A,1MDC A,1MDO A,1ME8 A,1MF7 A,1MFG A,1MFG B,1MG4 A,1MG7 A,1MG7 B,1MGP A,1MGQ A,1MGQ B,1MGQ C,1MGQ D,1MGQ E,1MGQ F,1MGQ G,1MGT A,1MH1 A,1MHN A,1MHW A,1MHW B,1MHW C,1MHW D,1MHW E,1MHW F,1MHW G,1MHW H,1MI3 A,1MI3 B,1MI3 C,1MI3 D,1MIX A,1MJ4 A,1MJ5 A,1MJF A,1MJF B,1MJH A,1MJH B,1MJN A,1MK0 A,1MK4 A,1MK4 B,1MKI A,1MKI B,1MKK A,1MKK B,1MKY A,1MKZ A,1MKZ B,1ML4 A,1ML9 A,1MML A,1MN8 A,1MN8 B,1MN8 C,1MN8 D,1MO0 A,1MO0 B,1MO9 A,1MO9 B,1MOF A,1MOQ A,1MP9 A,1MP9 B,1MPG A,1MPG B,1MPP A,1MPX A,1MPX B,1MPX C,1MPX D,1MQE A,1MQV A,1MQV B,1MRZ A,1MRZ B,1MSC A,1MTP A,1MTP B,1MTY B,1MTY C,1MTY D,1MTY E,1MTY G,1MTY H,1MUG A,1MUN A,1MUW A,1MV8 A,1MV8 B,1MV8 C,1MV8 D,1MVL A,1MVO A,1MW7 A,1MW9 X,1MWQ A,1MWQ B,1MX3 A,1MXG A,1MXR A,1MXR B,1MY7 A,1MY7 B,1MZ4 A,1MZ9 A,1MZ9 B,1MZ9 C,1MZ9 D,1MZ9 E,1MZB A,1MZH A,1MZH B,1MZU A,1MZU B,1MZU C,1N08 A,1N08 B,1N0Q A,1N0Q B,1N0R A,1N12 A,1N12 B,1N12 C,1N12 D,1N13 A,1N13 B,1N13 C,1N13 D,1N13 E,1N13 F,1N13 G,1N13 H,1N13 I,1N13 J,1N13 K,1N13 L,1N1B A,1N1B B,1N1F A,1N2M A,1N2M B,1N2M C,1N2M D,1N2M E,1N2M F,1N2S A,1N2Z A,1N2Z B,1N3L A,1N40 A,1N45 A,1N45 B,1N57 A,1N5U A,1N62 A,1N62 B,1N62 C,1N62 D,1N62 E,1N62 F,1N67 A,1N71 A,1N71 B,1N71 C,1N71 D,1N7E A,1N7H A,1N7H B,1N7K A,1N7K B,1N7O A,1N7S A,1N7S B,1N7S C,1N7S D,1N7Z A,1N7Z B,1N7Z C,1N7Z D,1N8V A,1N8V B,1N93 X,1N97 A,1N97 B,1N9L A,1N9P A,1NA0 A,1NA0 B,1NA3 A,1NA3 B,1NAR A,1NB9 A,1NBA A,1NBA B,1NBA C,1NBA D,1NC5 A,1NC7 A,1NC7 B,1NC7 C,1NC7 D,1NE2 A,1NE2 B,1NE7 A,1NE7 B,1NE7 C,1NE7 D,1NE7 E,1NE7 F,1NEP A,1NEU A,1NF9 A,1NFF A,1NFF B,1NFN A,1NFP A,1NFV A,1NFV B,1NFV C,1NFV D,1NFV E,1NFV F,1NFV G,1NFV H,1NFV I,1NFV

J,1NFV K,1NFV L,1NFV M,1NFV N,1NFV O,1NFV P,1NG2 A,1NG6 A,1NH8 A,1NHS A,1NIG A,1NIJ A,1NJH A,1NJK A,1NJK B,1NJK C,1NJK D,1NJR A,1NKD A,1NKI A,1NKI B,1NKO A,1NKR A,1NKZ A,1NKZ B,1NKZ C,1NKZ D,1NKZ E,1NKZ F,1NL1 A,1NLF A,1NLF B,1NLF C,1NLQ A,1NLQ B,1NLQ C,1NLQ D,1NLQ E,1NM2 A,1NM8 A,1NN5 A,1NNF A,1NNH A,1NNL A,1NNL B,1NNW A,1NNW B,1NNX A,1NO5 A,1NO5 B,1NOA A,1NOG A,1NOT A,1NOX A,1NP6 A,1NP6 B,1NP7 A,1NP7 B,1NP8 A,1NP8 B,1NPI A,1NPS A,1NPY A,1NPY B,1NPY C,1NPY D,1NQ7 A,1NQ7 B,1NQJ A,1NQJ B,1NQU A,1NQU B,1NQU C,1NQU D,1NQU E,1NQZ A,1NR0 A,1NR4 A,1NR4 B,1NR4 C,1NR4 D,1NR4 E,1NR4 F,1NR4 G,1NR4 H,1NRG A,1NRJ A,1NRJ B,1NRV A,1NRV B,1NRW A,1NRZ A,1NRZ B,1NRZ C,1NRZ D,1NS5 A,1NS5 B,1NSC A,1NSC B,1NSJ A,1NSZ A,1NSZ B,1NTH A,1NTV A,1NTV B,1NTY A,1NU0 A,1NU0 B,1NU4 A,1NU4 B,1NU5 A,1NUL A,1NUL B,1NUU A,1NUU B,1NUY A,1NWA A,1NWW A,1NWW B,1NWZ A,1NXC A,1NXJ A,1NXJ B,1NXJ C,1NXM A,1NXM B,1NXU A,1NXU B,1NYC A,1NYC B,1NYK A,1NYK B,1NZ0 A,1NZ0 B,1NZ0 C,1NZ0 D,1NZA A,1NZI A,1NZI B,1NZJ A,1NZN A,1O04 A,1O04 B,1O04 C,1O04 D,1O04 E,1O04 F,1O04 G,1O04 H,1O06 A,1O0S A,1O0S B,1O0W A,1O0W B,1O0X A,1O13 A,1O1Y A,1O1Z A,1O20 A,1O22 A,1O2D A,1O2D B,1O3U A,1O4S A,1O4S B,1O4T A,1O4T B,1O4W A,1O4Y A,1O50 A,1O54 A,1O5K A,1O5K B,1O5U A,1O5U B,1O5X A,1O5X B,1O63 A,1O63 B,1O69 A,1O69 B,1O6A A,1O6A B,1O6D A,1O6V A,1O6V B,1O75 A,1O75 B,1O7I A,1O7I B,1O7J A,1O7J B,1O7J C,1O7J D,1O7Q A,1O7Q B,1O7Z A,1O7Z B,1O8V A,1O8X A,1O91 A,1O91 B,1O91 C,1O98 A,1O9G A,1O9I A,1O9I B,1O9I C,1O9I D,1O9I E,1O9I F,1OA8 A,1OA8 B,1OA8 C,1OA8 D,1OAA A,1OAC A,1OAC B,1OAI A,1OAI B,1OAO A,1OAO B,1OAO C,1OAO D,1OB4 A,1OB6 A,1OB6 B,1OB7 A,1OB8 A,1OB8 B,1OBB A,1OBB B,1OBO A,1OBO B,1OC2 A,1OC2 B,1OC7 A,1OCK A,1OCK B,1OCY A,1OD3 A,1OD6 A,1ODK A,1ODK B,1ODK C,1ODK D,1ODK E,1ODK F,1ODM A,1ODO A,1ODV A,1ODV B,1ODZ A,1ODZ B,1OE8 A,1OE8 B,1OEJ A,1OEW A,1OEY A,1OEY B,1OEY C,1OEY D,1OEY J,1OEY K,1OEY L,1OEY M,1OF8 A,1OF8 B,1OFC X,1OFD A,1OFD B,1OFL A,1OFW A,1OFW B,1OFZ A,1OFZ B,1OGD A,1OGD B,1OGD C,1OGD D,1OGD E,1OGO X,1OGQ A,1OH0 A,1OH0 B,1OH4 A,1OHL A,1OHP A,1OHP B,1OHP C,1OHP D,1OHT A,1OI0 A,1OI0 B,1OI0 C,1OI0 D,1OI2 A,1OI2 B,1OI6 A,1OI6 B,1OI7 A,1OIH A,1OIH B,1OIH C,1OIH D,1OIS A,1OJH A,1OJH B,1OJH C,1OJH D,1OJH E,1OJH F,1OJH G,1OJH H,1OJH I,1OJH J,1OJH K,1OJH L,1OJJ A,1OJJ B,1OJQ A,1OK0 A,1OKI A,1OKI B,1OKS A,1OLL A,1OLR A,1OLZ A,1OLZ B,1ON2 A,1ON2 B,1ON3 A,1ON3 B,1ON3 C,1ON3 D,1ON3 E,1ON3 F,1ONW A,1ONW B,1OO0 A,1OO0 B,1OOE A,1OOE B,1OOH A,1OOH B,1OOT A,1OPC A,1OPK A,1OQ1 A,1OQ1 B,1OQ1 C,1OQ1 D,1OQJ A,1OQJ B,1OQV A,1OQV B,1OQV C,1OQW A,1OQW B,1OR0 A,1OR0 B,1OR0 C,1OR0 D,1OR7 A,1OR7 B,1OR7 C,1OR7 F,1ORR A,1ORR B,1ORR C,1ORR D,1ORU A,1ORU B,1OSD A,1OSD B,1OSY A,1OSY B,1OTH A,1OTK A,1OTK B,1OU8 A,1OU8 B,1OU8 C,1OU8 D,1OU9 A,1OU9 B,1OU9 C,1OUV A,1OUW A,1OUW B,1OUW C,1OUW D,1OV3 A,1OV3 B,1OV3 C,1OV3 D,1OW1 A,1OW4 A,1OW4 B,1OWL A,1OX0 A,1OX3 A,1OXJ A,1OXX K,1OYG A,1OYW A,1OZ2 A,1OZ9 A,1OZH A,1OZH B,1OZH C,1OZH D,1OZN A,1P0H A,1P0K A,1P0K B,1P0Z A,1P0Z B,1P0Z C,1P0Z D,1P0Z E,1P0Z F,1P0Z G,1P0Z H,1P0Z I,1P0Z J,1P15 A,1P15 B,1P1J A,1P1J B,1P1L A,1P1M A,1P2F A,1P3C A,1P3D A,1P3D B,1P4C A,1P4O A,1P4O B,1P4P A,1P57 A,1P57 B,1P5D X,1P5Z

B,1P6O A,1P6O B,1P6X A,1P6X B,1P77 A,1P90 A,1P99 A,1P9A G,1P9B A,1P9G A,1P9H A,1P9I A,1PB7 A,1PBE A,1PBJ A,1PBW A,1PBW B,1PBY A,1PBY B,1PBY C,1PCF A,1PCF B,1PCF C,1PCF D,1PCF E,1PCF F,1PCF G,1PCF H,1PCH A,1PDO A,1PE9 A,1PE9 B,1PEF A,1PEN A,1PFB A,1PFB B,1PFZ A,1PFZ B,1PFZ C,1PFZ D,1PG4 A,1PG4 B,1PG6 A,1PGV A,1PGX A,1PII A,1PJ5 A,1PK3 A,1PK3 B,1PK3 C,1PKH A,1PKH B,1PKO A,1PL3 A,1PL3 B,1PM1 X,1PM4 A,1PM4 B,1PM4 C,1PMH X,1PMI A,1PMM A,1PMM B,1PMM C,1PMM D,1PMM E,1PMM F,1PN0 A,1PN0 B,1PN0 C,1PN0 D,1PN2 A,1PN2 B,1PN2 C,1PN2 D,1POC A,1POT A,1PP0 A,1PP0 B,1PP0 C,1PP0 D,1PPR M,1PPR N,1PPR O,1PPY A,1PPY B,1PQ4 A,1PQ4 B,1PQ7 A,1PQH A,1PQH B,1PRZ A,1PSR A,1PSR B,1PSW A,1PT6 A,1PT6 B,1PTF A,1PTQ A,1PU6 A,1PU6 B,1PUC A,1PUI A,1PUI B,1PUJ A,1PUO A,1PUO B,1PV5 A,1PVG A,1PVG B,1PVM A,1PVM B,1PVN A,1PVN B,1PVN C,1PVN D,1PWA A,1PWB A,1PWB B,1PWB C,1PX5 A,1PX5 B,1PXZ A,1PXZ B,1PY9 A,1PYF A,1PYO A,1PYO B,1PYO C,1PYO D,1PYO E,1PYO F,1PYZ A,1PYZ B,1PZ4 A,1PZ7 A,1PZ7 B,1PZS A,1PZW A,1PZX A,1PZX B,1Q08 A,1Q08 B,1Q0P A,1Q0Q A,1Q0Q B,1Q0R A,1Q0U A,1Q0U B,1Q1A A,1Q1A B,1Q1F A,1Q1R A,1Q1R B,1Q1U A,1Q25 A,1Q2H A,1Q2H B,1Q2H C,1Q2Y A,1Q33 A,1Q35 A,1Q3L A,1Q3L P,1Q3O A,1Q3O B,1Q42 A,1Q4U A,1Q4U B,1Q5D A,1Q5Y A,1Q5Y B,1Q5Y C,1Q5Y D,1Q5Z A,1Q6H A,1Q6H B,1Q6O A,1Q6O B,1Q6Z A,1Q74 A,1Q74 B,1Q74 C,1Q74 D,1Q7E A,1Q7F A,1Q7F B,1Q7L A,1Q7L B,1Q7L C,1Q7L D,1Q8B A,1Q8C A,1Q8D A,1Q8F A,1Q8F B,1Q8F C,1Q8F D,1Q8H A,1Q8I A,1Q9U A,1Q9U B,1QAD A,1QAH A,1QAH B,1QAU A,1QAZ A,1QB0 A,1QB7 A,1QBA A,1QBZ A,1QBZ B,1QBZ C,1QCS A,1QCX A,1QD1 A,1QD1 B,1QD8 A,1QD8 B,1QDD A,1QE3 A,1QF8 A,1QF8 B,1QF9 A,1QFI A,1QFI B,1QFI C,1QFO A,1QFO B,1QFO C,1QFT A,1QFT B,1QGE D,1QGE E,1QGI A,1QGQ A,1QGV A,1QH4 A,1QH4 B,1QH4 C,1QH4 D,1QH5 A,1QH5 B,1QHD A,1QHO A,1QHQ A,1QHV A,1QJ8 A,1QJC A,1QJC B,1QJP A,1QKK A,1QKR A,1QKR B,1QKS A,1QKS B,1QL3 A,1QL3 B,1QL3 C,1QL3 D,1QLW A,1QLW B,1QNR A,1QNX A,1QO2 A,1QO2 B,1QOW A,1QOW B,1QOW C,1QOW D,1QOW E,1QOW F,1QQ5 A,1QQ5 B,1QQF A,1QQP 1,1QQP 2,1QQP 3,1QQP 4,1QR0 A,1QRE A,1QS1 A,1QS1 B,1QS1 C,1QS1 D,1QSA A,1QTN A,1QTN B,1QTN C,1QTW A,1QU1 A,1QU1 B,1QU1 C,1QU1 D,1QU1 E,1QU1 F,1QU9 A,1QU9 B,1QU9 C,1QUP A,1QUP B,1QUS A,1QV1 A,1QVE A,1QVE B,1QW2 A,1QW9 A,1QW9 B,1QWD A,1QWD B,1QWG A,1QWI A,1QWI B,1QWI C,1QWI D,1QWK A,1QWO A,1QWR A,1QWR B,1QWY A,1QWZ A,1QX2 A,1QX2 B,1QXO A,1QXO B,1QXO C,1QXO D,1QXY A,1QYA A,1QYA B,1QZ1 A,1QZ9 A,1QZM A,1R0D A,1R0D B,1R0D D,1R0D E,1R0D F,1R0D G,1R0D H,1R0D I,1R0M A,1R0M B,1R0M C,1R0M D,1R0U A,1R0V A,1R0V B,1R0V C,1R0V D,1R17 A,1R17 B,1R17 C,1R17 D,1R1H A,1R1M A,1R1T A,1R1T B,1R26 A,1R29 A,1R2Q A,1R3D A,1R3F A,1R3S A,1R45 A,1R45 B,1R45 C,1R45 D,1R4V A,1R4X A,1R55 A,1R5L A,1R5M A,1R5Q A,1R5T A,1R5T B,1R5T C,1R5T D,1R62 A,1R69 A,1R6D A,1R6J A,1R6V A,1R6W A,1R6X A,1R75 A,1R77 A,1R77 B,1R7A A,1R7A B,1R7J A,1R7L A,1R7L B,1R85 A,1R88 A,1R88 B,1R89 A,1R8H A,1R8H B,1R8H C,1R8H D,1R8H E,1R8H F,1R8N A,1R8O A,1R8O B,1R9D A,1R9D B,1R9H A,1R9L A,1R9W A,1RA0 A,1RC9 A,1RCQ A,1REG X,1REG Y,1RF6 A,1RF6 B,1RF6 C,1RF6 D,1RFE A,1RFS A,1RFX A,1RFX B,1RFX C,1RFY A,1RFY B,1RG8 A,1RG8 B,1RGX A,1RGX B,1RGX C,1RH9 A,1RHC A,1RHF A,1RHF B,1RHS A,1RI6 A,1RIE A,1RIF A,1RIF B,1RJD A,1RJD B,1RJD C,1RJU V,1RK6 A,1RKI

A,1RKI B,1RKQ A,1RKQ B,1RKT A,1RKT B,1RKU A,1RKU B,1RL0 A,1RL6
A,1RLH A,1RLI A,1RLI B,1RLI C,1RLI D,1RLJ A,1RM6 A,1RM6 B,1RM6
C,1RM6 D,1RM6 E,1RM6 F,1RMG A,1RO2 A,1ROC A,1ROW A,1ROW
B,1RP0 A,1RP0 B,1RQB A,1RQP A,1RQP B,1RQP C,1RRK A,1RRM A,1RRM
B,1RSG A,1RSG B,1RSS A,1RT8 A,1RTQ A,1RTT A,1RU4 A,1RUT X,1RUW
A,1RW1 A,1RWH A,1RWJ A,1RWR A,1RWZ A,1RX0 A,1RX0 B,1RX0 C,1RX0
D,1RXD A,1RXD B,1RXD C,1RXQ A,1RXQ B,1RXQ C,1RXQ D,1RY6 A,1RY9
A,1RY9 B,1RY9 C,1RY9 D,1RYB A,1RYI A,1RYI B,1RYI C,1RYI D,1RYL
A,1RYL B,1RYO A,1RYP 1,1RYP 2,1RYP A,1RYP B,1RYP C,1RYP D,1RYP
E,1RYP F,1RYP G,1RYP H,1RYP I,1RYP J,1RYP K,1RYP L,1RYP M,1RYP
N,1RYP O,1RYP P,1RYP Q,1RYP R,1RYP S,1RYP T,1RYP U,1RYP V,1RYP
W,1RYP X,1RYP Y,1RYP Z,1RYQ A,1RZ2 A,1RZ3 A,1S0P A,1S0P B,1S12
A,1S12 B,1S12 C,1S12 D,1S1D A,1S1D B,1S1F A,1S21 A,1S29 A,1S2O
A,1S2W A,1S2X A,1S3Z A,1S3Z B,1S4K A,1S4K B,1S55 A,1S55 B,1S55
C,1S5A A,1S5A B,1S5A C,1S5A D,1S5P A,1S5P B,1S5U A,1S5U B,1S5U
C,1S5U D,1S5U E,1S5U F,1S5U G,1S5U H,1S67 L,1S67 U,1S68 A,1S6C
A,1S6C B,1S7I A,1S7K A,1S7Z A,1S8N A,1S96 A,1S96 B,1S99 A,1S99
B,1S9R A,1S9R B,1S9U A,1SAU A,1SBP A,1SBX A,1SBY A,1SBY B,1SBZ
A,1SBZ B,1SBZ C,1SBZ D,1SD4 A,1SD4 B,1SDI A,1SDO A,1SE0 A,1SE0
B,1SE8 A,1SEI A,1SEI B,1SEN A,1SF9 A,1SFF A,1SFF B,1SFF C,1SFF
D,1SFL A,1SFL B,1SFP A,1SFS A,1SFX A,1SFX B,1SG4 A,1SG4 B,1SG4
C,1SGJ A,1SGJ B,1SGJ C,1SGM A,1SGM B,1SGV A,1SGV B,1SGW A,1SH8
A,1SH8 B,1SHO A,1SHO B,1SHU X,1SJ1 A,1SJ1 B,1SJD A,1SJD B,1SJD
C,1SJD D,1SJN A,1SJN B,1SJN C,1SJW A,1SJY A,1SK4 A,1SK7 A,1SKZ
A,1SLM A,1SMO A,1SMO B,1SMX A,1SMX B,1SN9 A,1SN9 B,1SN9 C,1SN9
D,1SNG A,1SNN A,1SNN B,1SNY A,1SO7 A,1SOX A,1SOX B,1SPV A,1SQ9
A,1SQG A,1SQH A,1SQS A,1SQS B,1SQW A,1SR4 A,1SR4 B,1SR4 C,1SR8
A,1SRA A,1SRR A,1SRR B,1SRR C,1SRV A,1SS4 A,1SS4 B,1STM A,1STM
B,1STM C,1STM D,1STM E,1SU8 A,1SUM B,1SUR A,1SUU A,1SVB A,1SVF
A,1SVF B,1SVF C,1SVF D,1SVI A,1SVM A,1SVM B,1SVM C,1SVM D,1SVM
E,1SVM F,1SVY A,1SW5 A,1SW5 B,1SW5 C,1SW5 D,1SX7 A,1SXR A,1SXR
B,1SXV A,1SY7 A,1SY7 B,1SYY A,1SZ7 A,1SZH A,1SZH B,1SZN A,1SZO
A,1SZO B,1SZO C,1SZO D,1SZO E,1SZO F,1SZO G,1SZO H,1SZO I,1SZO
J,1SZO K,1SZO L,1SZW A,1SZW B,1T07 A,1T0B A,1T0B B,1T0B C,1T0B
D,1T0B E,1T0B F,1T0B G,1T0B H,1T0F A,1T0F B,1T0F C,1T0F D,1T0H
A,1T0H B,1T0I A,1T0I B,1T0T V,1T0T W,1T0T X,1T0T Y,1T0T Z,1T1E A,1T1G
A,1T1J A,1T1J B,1T1U A,1T1V A,1T1V B,1T2A A,1T2A B,1T2A C,1T2A
D,1T2W A,1T2W B,1T2W C,1T3Q A,1T3Q B,1T3Q C,1T3Q D,1T3Q E,1T3Q
F,1T3Y A,1T4A A,1T4A B,1T5I A,1T5O A,1T5O B,1T5O C,1T5O D,1T6C
A,1T6E X,1T6F A,1T6F B,1T6L A,1T6N A,1T6N B,1T6O A,1T6O B,1T6O
L,1T6S A,1T6S B,1T6T 1,1T6T 2,1T6U A,1T6U B,1T6U C,1T6U D,1T6U
E,1T6U F,1T6U G,1T6U H,1T6U I,1T6U J,1T6U K,1T6U L,1T7H A,1T7H
B,1T7L A,1T7L B,1T7V A,1T82 A,1T82 B,1T82 C,1T82 D,1T8H A,1T8K A,1T8T
A,1T8T B,1T92 A,1T92 B,1T9F A,1T9H A,1TA9 A,1TA9 B,1TAF A,1TAF
B,1TAZ A,1TBF A,1TC1 A,1TC1 B,1TC5 A,1TC5 B,1TC5 C,1TC5 D,1TCA
A,1TCV A,1TCV B,1TCV C,1TD4 A,1TE2 A,1TE2 B,1TE5 A,1TE5 B,1TEN
A,1TFE A,1TFZ A,1TG0 A,1TGR A,1TGR B,1TGX A,1TGX B,1TGX C,1TH7
A,1TH7 B,1TH7 C,1TH7 D,1TH7 E,1TH7 F,1TH7 G,1TH7 H,1TH7 I,1TH7
J,1TH7 K,1TH7 L,1TH7 M,1TH7 N,1THG A,1THM A,1THX A,1TIB A,1TIF
A,1TIG A,1TIQ A,1TIQ B,1TJB A,1TJB B,1TJL A,1TJL B,1TJL C,1TJL D,1TJL

E,1TJL F,1TJL G,1TJL H,1TJL I,1TJL J,1TJO A,1TJO B,1TJO C,1TJO D,1TJV A,1TJV B,1TJV C,1TJV D,1TK1 A,1TKE A,1TKI A,1TKI B,1TKJ A,1TKS A,1TKS B,1TL2 A,1TMX A,1TMX B,1TN3 A,1TOA A,1TOA B,1TOL A,1TOV A,1TP5 A,1TP5 B,1TP6 A,1TP9 A,1TP9 B,1TP9 C,1TP9 D,1TQ5 A,1TQG A,1TQH A,1TQJ A,1TQJ B,1TQJ C,1TQJ D,1TQJ E,1TQJ F,1TQY A,1TQY B,1TQY C,1TQY D,1TQY E,1TQY F,1TQY G,1TQY H,1TR0 A,1TR0 B,1TR0 C,1TR0 D,1TR0 E,1TR0 F,1TR0 G,1TR0 H,1TR0 I,1TR0 J,1TR0 K,1TR0 L,1TR0 M,1TR0 N,1TR0 O,1TR0 P,1TR0 R,1TR0 S,1TR0 T,1TR0 U,1TR0 V,1TR0 W,1TR0 X,1TR0 Y,1TS9 A,1TT8 A,1TU1 A,1TU1 B,1TU7 A,1TU7 B,1TU9 A,1TUA A,1TUH A,1TUK A,1TUO A,1TUV A,1TUW A,1TVD A,1TVD B,1TVF A,1TVF B,1TVG A,1TVN A,1TVN B,1TVX A,1TVX B,1TVX C,1TVX D,1TW9 A,1TW9 B,1TW9 C,1TW9 D,1TW9 E,1TW9 F,1TW9 G,1TW9 H,1TWD A,1TWD B,1TWI A,1TWI B,1TWI C,1TWI D,1TWU A,1TWY A,1TWY B,1TWY C,1TWY D,1TWY E,1TWY F,1TWY G,1TWY H,1TXG A,1TXG B,1TXJ A,1TXL A,1TXN A,1TXN B,1TXO A,1TXO B,1TY0 A,1TY0 B,1TY0 C,1TYJ A,1TZ0 A,1TZ0 B,1TZ0 C,1TZJ A,1TZJ B,1TZJ C,1TZJ D,1TZP A,1TZP B,1TZV A,1U00 A,1U00 P,1U02 A,1U07 A,1U07 B,1U0K A,1U0K B,1U14 A,1U2H A,1U2K A,1U2P A,1U2W A,1U2W B,1U2W C,1U2W D,1U53 A,1U5D A,1U5D B,1U5D C,1U5D D,1U5F A,1U5H A,1U5K A,1U5K B,1U5P A,1U5U A,1U5U B,1U60 A,1U60 B,1U60 C,1U60 D,1U69 A,1U69 B,1U69 C,1U69 D,1U6E A,1U6E B,1U6K A,1U6K B,1U6K C,1U6T A,1U6Z A,1U6Z B,1U79 A,1U79 B,1U79 C,1U79 D,1U79 E,1U7B A,1U7B B,1U7G A,1U7I A,1U7I B,1U7K A,1U7K B,1U7K C,1U7K D,1U7K E,1U7K F,1U7L A,1U7P A,1U7P B,1U7P C,1U7P D,1U84 A,1U8V A,1U8V B,1U8V C,1U8V D,1U94 A,1U9C A,1U9D A,1U9D B,1U9L A,1U9L B,1U9L C,1U9P A,1UA4 A,1UAI A,1UAN A,1UAN B,1UAR A,1UAS A,1UCD A,1UCR A,1UCR B,1UCS A,1UD9 A,1UD9 B,1UD9 C,1UD9 D,1UEB A,1UEB B,1UEK A,1UF5 A,1UF5 B,1UFB A,1UFB B,1UFB C,1UFB D,1UFI A,1UFI B,1UFI C,1UFI D,1UFO A,1UFO B,1UFO C,1UFO D,1UFO E,1UFO F,1UFY A,1UG6 A,1UGI A,1UGI B,1UGI C,1UGI D,1UGI E,1UGI F,1UGI G,1UGI H,1UHA A,1UHE A,1UHE B,1UI0 A,1UII A,1UII B,1UIR A,1UIR B,1UIX A,1UIX B,1UJ0 A,1UJ0 B,1UJ2 A,1UJ2 B,1UJ6 A,1UJ8 A,1UJC A,1UJN A,1UJN B,1UJP A,1UK8 A,1UKF A,1UKK A,1UKK B,1UKU A,1ULK A,1ULK B,1ULR A,1UM0 A,1UM0 B,1UM0 C,1UM0 D,1UMD A,1UMD B,1UMD C,1UMD D,1UMZ A,1UMZ B,1UNO A,1UNO B,1UNQ A,1UOH A,1UOW A,1UOY A,1UOZ A,1UP9 A,1UPI A,1UPK A,1UPK B,1UPQ A,1UPS A,1UPS B,1UQT A,1UQT B,1UR1 A,1URR A,1URS A,1URS B,1US0 A,1US3 A,1US5 A,1USC A,1USC B,1USE A,1USG A,1USM A,1USR A,1USR B,1UT1 A,1UT1 B,1UT1 C,1UT1 D,1UT1 E,1UT1 F,1UT7 A,1UT7 B,1UTE A,1UTG A,1UTI A,1UTI D,1UUF A,1UUJ A,1UUJ B,1UUJ C,1UUJ D,1UUQ A,1UUY A,1UV7 A,1UV7 B,1UW4 A,1UW4 B,1UW4 C,1UW4 D,1UWC A,1UWC B,1UWK A,1UWK B,1UWM A,1UWV A,1UWW A,1UWW B,1UX6 A,1UXO A,1UXX X,1UXY A,1UY4 A,1UYP A,1UYP B,1UYP C,1UYP D,1UYP E,1UYP F,1UZ3 A,1UZ3 B,1UZK A,1UZM A,1UZM B,1V05 A,1V0A A,1V0L A,1V1H A,1V1H B,1V1H C,1V1H D,1V1H E,1V1H F,1V2D A,1V2X A,1V2Z A,1V30 A,1V33 A,1V37 A,1V37 B,1V3W A,1V3Y A,1V3Y B,1V4A A,1V4F A,1V4F B,1V4F C,1V4P A,1V4P B,1V4P C,1V4V A,1V4V B,1V58 A,1V58 B,1V5D A,1V5D B,1V5V A,1V5V B,1V5X A,1V5X B,1V6P A,1V6P B,1V6Q A,1V6Q B,1V6Q C,1V6T A,1V6Z A,1V6Z B,1V70 A,1V74 A,1V74 B,1V76 A,1V76 B,1V77 A,1V7H A,1V7H B,1V7H C,1V7L A,1V7L B,1V7R A,1V7W A,1V7Z A,1V7Z B,1V7Z C,1V7Z D,1V7Z E,1V7Z F,1V84 A,1V84 B,1V8C A,1V8C B,1V8C

C,1V8C D,1V8H A,1V8H B,1V8Y A,1V96 A,1V96 B,1V98 A,1V98 B,1V9F A,1V9K A,1V9K B,1V9M A,1V9Y A,1V9Y B,1VA0 A,1VA0 B,1VAJ A,1VAV A,1VAV B,1VBI A,1VBK A,1VBK B,1VBW A,1VC1 A,1VC1 B,1VC3 A,1VC3 B,1VC4 A,1VC4 B,1VCA A,1VCA B,1VCC A,1VCD A,1VCD B,1VCH A,1VCH B,1VCH C,1VCH D,1VCH E,1VCL A,1VCL B,1VCT A,1VCV A,1VCV B,1VD6 A,1VDW A,1VDW B,1VE0 A,1VE1 A,1VE2 A,1VE2 B,1VE4 A,1VEF A,1VEF B,1VEM A,1VF8 A,1VFJ A,1VFJ B,1VFJ C,1VFR A,1VFR B,1VFS A,1VFS B,1VFY A,1VG8 A,1VG8 B,1VG8 C,1VG8 D,1VGJ A,1VH4 A,1VH4 B,1VH5 A,1VH5 B,1VHC A,1VHC B,1VHC C,1VHC D,1VHC E,1VHC F,1VHE A,1VHF A,1VHN A,1VHO A,1VHQ A,1VHQ B,1VHS A,1VHS B,1VHT A,1VHT B,1VHT C,1VHU A,1VHV A,1VHV B,1VHX A,1VHX B,1VHY A,1VHY B,1VI0 A,1VI0 B,1VI4 A,1VI6 A,1VI6 B,1VI6 C,1VI6 D,1VIA A,1VIA B,1VIM A,1VIM B,1VIM C,1VIM D,1VIO A,1VIO B,1VJ0 A,1VJ0 B,1VJ0 C,1VJ0 D,1VJ2 A,1VJ2 B,1VJE A,1VJE B,1VJK A,1VJL A,1VJL B,1VJN A,1VJN B,1VJO A,1VJU A,1VJU B,1VJV A,1VK1 A,1VK2 A,1VK4 A,1VK8 A,1VK8 B,1VK8 C,1VK8 D,1VKA A,1VKA B,1VKC A,1VKC B,1VKE A,1VKE B,1VKE C,1VKE D,1VKE E,1VKE F,1VKF A,1VKF B,1VKF C,1VKF D,1VKH A,1VKH B,1VKI A,1VKI B,1VKK A,1VKM A,1VKM B,1VKM C,1VKM D,1VKM E,1VKM F,1VKN A,1VKN B,1VKN C,1VKN D,1VKU A,1VKW A,1VKY A,1VKY B,1VL1 A,1VL2 A,1VL2 B,1VL2 C,1VL2 D,1VL4 A,1VL4 B,1VL5 A,1VL5 B,1VL5 C,1VL5 D,1VL7 A,1VL7 B,1VLA A,1VLA B,1VLA C,1VLA D,1VLG A,1VLG B,1VLG C,1VLG D,1VLG E,1VLG F,1VLG G,1VLG H,1VLJ A,1VLJ B,1VLO A,1VLP A,1VLP B,1VLP C,1VLP D,1VLR A,1VLR B,1VLY A,1VMA A,1VMA B,1VMB A,1VME A,1VME B,1VMG A,1VMH A,1VMJ A,1VP2 A,1VP2 B,1VP4 A,1VP4 B,1VP8 A,1VPB A,1VPD A,1VPH A,1VPH B,1VPH C,1VPH D,1VPH E,1VPH F,1VPK A,1VPM A,1VPM B,1VPM C,1VPR A,1VPT A,1VQ3 A,1VQ3 B,1VQ3 C,1VQ3 D,1VQQ A,1VQQ B,1VQS A,1VQS B,1VQS C,1VQS D,1VQS E,1VQT A,1VQU A,1VQU B,1VQZ A,1VR6 A,1VR6 B,1VR6 C,1VR6 D,1VR7 A,1VR7 B,1VR8 A,1VR9 A,1VR9 B,1VRA A,1VRA B,1VRM A,1VRZ A,1VSR A,1VYB A,1VYB B,1VYF A,1VYI A,1VYK A,1VYR A,1VZI A,1VZI B,1VZM A,1VZM B,1VZM C,1VZY A,1VZY B,1W07 A,1W07 B,1W0D A,1W0D B,1W0D C,1W0D D,1W0H A,1W0N A,1W0P A,1W15 A,1W1H A,1W1H B,1W1H C,1W1H D,1W1O A,1W23 A,1W23 B,1W27 A,1W27 B,1W2F A,1W2F B,1W2I A,1W2I B,1W2L A,1W2W A,1W2W B,1W2W E,1W2W F,1W2W I,1W2W J,1W2W M,1W2W N,1W2Y A,1W2Y B,1W32 A,1W32 B,1W3I A,1W3I B,1W3I C,1W3I D,1W3M A,1W3M B,1W3M C,1W3M D,1W3M E,1W3M F,1W3M G,1W3M H,1W3M I,1W3M J,1W3M K,1W3M L,1W41 A,1W4R A,1W4R B,1W4R C,1W4R D,1W4R E,1W4R F,1W4R G,1W4R H,1W4S A,1W4T A,1W4V A,1W4V B,1W4V C,1W4V D,1W4V E,1W4V F,1W53 A,1W5F A,1W5F B,1W5Q A,1W5Q B,1W5R A,1W5R B,1W5U A,1W5U B,1W5U C,1W5U D,1W66 A,1W6S A,1W6S B,1W6S C,1W6S D,1W6U A,1W6U B,1W6U C,1W6U D,1W70 A,1W70 B,1W70 C,1W70 D,1W78 A,1W79 A,1W79 B,1W79 C,1W79 D,1W7C A,1W7J A,1W7J B,1W7Q A,1W7Q B,1W7Q C,1W7Q D,1W7Q E,1W7Q F,1W7R A,1W7R B,1W7R C,1W7R D,1W7R E,1W7R F,1W7R G,1W7R H,1W8K A,1W8O A,1W8S A,1W8S B,1W8S C,1W8S D,1W8S E,1W8S F,1W8S G,1W8S H,1W8S I,1W8S J,1W94 A,1W94 B,1W96 A,1W96 B,1W96 C,1W99 A,1W9A A,1W9A B,1W9E A,1W9E B,1W9E R,1W9E S,1W9E T,1W9H A,1W9I A,1W9S A,1W9S B,1WA3 A,1WA3 B,1WA3 C,1WA3 D,1WA3 E,1WA3 F,1WAK A,1WB0 A,1WB0 B,1WB4 A,1WB4 B,1WBA A,1WC2 A,1WC3 A,1WC3 B,1WCG A,1WCG B,1WCH A,1WCK A,1WCU A,1WCV 1,1WCW A,1WD3 A,1WD5 A,1WDC A,1WDC

B,1WDC C,1WDD A,1WDD E,1WDD S,1WDD W,1WDE A,1WDJ A,1WDJ B,1WDJ C,1WDN A,1WDV A,1WDV B,1WDY A,1WEH A,1WEH B,1WER A,1WF3 A,1WFB A,1WFB B,1WG8 A,1WG8 B,1WHI A,1WHS A,1WHS B,1WHZ A,1WIW A,1WIW B,1WJ9 A,1WJX A,1WKA A,1WKC A,1WKO A,1WKO B,1WKQ A,1WKQ B,1WKR A,1WKR I,1WKU A,1WKU B,1WKV A,1WKV B,1WKY A,1WL8 A,1WLE A,1WLE B,1WLG A,1WLG B,1WLJ A,1WLT A,1WLT B,1WLU A,1WLY A,1WLZ A,1WLZ B,1WLZ C,1WLZ D,1WM3 A,1WMA A,1WMD A,1WMH A,1WMH B,1WMS A,1WMS B,1WMW A,1WMW B,1WMW C,1WMW D,1WMX A,1WMX B,1WN2 A,1WNA A,1WNY A,1WNY B,1WOC A,1WOC B,1WOC C,1WOC D,1WOH A,1WOH B,1WOH C,1WOH D,1WOH E,1WOH F,1WOL A,1WOQ A,1WOQ B,1WOS A,1WOU A,1WOV A,1WOV B,1WOZ A,1WP5 A,1WPA A,1WPB A,1WPB B,1WPB C,1WPB D,1WPB E,1WPB F,1WPB G,1WPB H,1WPB I,1WPB J,1WPB K,1WPB L,1WPB M,1WPB N,1WPB O,1WPB P,1WPN A,1WPN B,1WPO A,1WPO B,1WPV A,1WPV B,1WPV C,1WQ6 A,1WQ6 B,1WQ8 A,1WQA A,1WQA B,1WQA C,1WQA D,1WQJ B,1WQJ I,1WR2 A,1WR8 A,1WR8 B,1WRA A,1WRA B,1WRJ A,1WS0 A,1WS8 A,1WS8 B,1WS8 C,1WS8 D,1WSR A,1WSR B,1WT6 A,1WT6 B,1WT6 D,1WTJ A,1WTJ B,1WU4 A,1WU9 A,1WU9 B,1WUB A,1WUR A,1WUR B,1WUR C,1WUR D,1WUR E,1WV3 A,1WV9 A,1WV9 B,1WVF A,1WVG A,1WVG B,1WVH A,1WVQ A,1WVQ B,1WVQ C,1WVV A,1WVV B,1WWC A,1WWI A,1WWJ A,1WWJ B,1WWJ C,1WWJ D,1WWK A,1WWK B,1WWR A,1WWR B,1WWR C,1WWR D,1WWZ A,1WWZ B,1WX1 A,1WX1 B,1WXX A,1WXX B,1WXX C,1WXX D,1WY1 A,1WY1 B,1WY1 C,1WY2 A,1WY2 B,1WY3 A,1WYX A,1WYX B,1WZ3 A,1WZ3 B,1WZA A,1WZD A,1WZD B,1WZL A,1WZL B,1WZN A,1WZN B,1WZN C,1WZO A,1WZO B,1WZO C,1WZO D,1WZZ A,1X0L A,1X0L B,1X0P A,1X0P B,1X0P C,1X0P D,1X0P E,1X0P F,1X0P G,1X0P H,1X0P I,1X0P J,1X0T A,1X13 A,1X13 B,1X1I A,1X1N A,1X1O A,1X1O B,1X1O C,1X2I A,1X2I B,1X3K A,1X3S A,1X3X A,1X3X B,1X46 A,1X54 A,1X6I A,1X6I B,1X6O A,1X6V A,1X6V B,1X6Z A,1X7D A,1X7D B,1X7V A,1X7V B,1X7V C,1X8B A,1X8D A,1X8D B,1X8D C,1X8D D,1X8Q A,1X91 A,1X9D A,1X9I A,1X9I B,1X9U A,1X9U B,1XA1 A,1XA1 B,1XA1 C,1XA1 D,1XAK A,1XAU A,1XAW A,1XBI A,1XCR A,1XCR B,1XDN A,1XDW A,1XDZ A,1XE0 A,1XE0 B,1XE0 C,1XE0 D,1XE0 E,1XE0 F,1XE0 G,1XE0 H,1XE0 I,1XE0 J,1XE1 A,1XE7 A,1XE7 B,1XE7 C,1XED A,1XED B,1XED C,1XED D,1XED E,1XED F,1XEO A,1XER A,1XFC A,1XFC B,1XFF A,1XFF B,1XFJ A,1XFK A,1XFS A,1XFS B,1XG0 A,1XG0 B,1XG0 C,1XG0 D,1XG4 A,1XG4 B,1XG4 C,1XG4 D,1XG5 A,1XG5 B,1XG5 C,1XG5 D,1XG7 A,1XG7 B,1XGK A,1XGS A,1XGS B,1XGW A,1XHD A,1XHK A,1XHK B,1XHN A,1XHN B,1XHN C,1XHN D,1XI3 A,1XI3 B,1XIO A,1XIY A,1XIY B,1XJT A,1XJU A,1XJU B,1XK7 A,1XK7 B,1XK7 C,1XKG A,1XKI A,1XKL A,1XKL B,1XKL C,1XKL D,1XKN A,1XKP A,1XKP B,1XKP C,1XKR A,1XKW A,1XKY A,1XKY B,1XKY C,1XKY D,1XLQ A,1XLQ B,1XLQ C,1XLY A,1XLY B,1XM3 A,1XM3 B,1XM3 C,1XM3 D,1XM8 A,1XM8 B,1XMC A,1XMC B,1XMK A,1XMT A,1XNF A,1XNF B,1XNG A,1XNG B,1XO5 A,1XO5 B,1XOC A,1XOC B,1XOD A,1XOD B,1XOV A,1XPH A,1XPP A,1XPP B,1XPP C,1XPP D,1XQA A,1XQA B,1XQO A,1XRK A,1XRK B,1XRO A,1XRT A,1XRT B,1XRU A,1XRU B,1XS1 A,1XS1 B,1XS1 C,1XS1 D,1XS1 E,1XS1 F,1XS5 A,1XSV A,1XSV B,1XSZ A,1XSZ B,1XT5 A,1XT8 A,1XT8 B,1XTE A,1XTI A,1XTM A,1XTM B,1XTP A,1XTT A,1XTT B,1XTT C,1XTT D,1XU1 A,1XU1 B,1XU1 D,1XU1 R,1XU1 S,1XU1 T,1XU9 A,1XU9 B,1XU9 C,1XU9

D,1XUB A,1XV2 A,1XV2 B,1XV2 C,1XV2 D,1XV5 A,1XVW A,1XVW B,1XVX A,1XW3 A,1XW8 A,1XWL A,1XWV A,1XWV B,1XWY A,1XX6 A,1XX6 B,1XY1 A,1XY1 B,1XY2 A,1XYZ A,1XYZ B,1XZZ A,1Y02 A,1Y07 A,1Y07 B,1Y07 C,1Y07 D,1Y08 A,1Y0B A,1Y0B B,1Y0B C,1Y0B D,1Y0H A,1Y0H B,1Y0K A,1Y0M A,1Y0N A,1Y0P A,1Y0U A,1Y0U B,1Y0Y A,1Y0Y B,1Y12 A,1Y12 B,1Y12 C,1Y1P A,1Y1P B,1Y1X A,1Y1X B,1Y2K A,1Y2K B,1Y2M A,1Y2M B,1Y2M C,1Y2M D,1Y43 A,1Y43 B,1Y4C A,1Y4J A,1Y4J B,1Y4M A,1Y4M B,1Y4M C,1Y4W A,1Y55 X,1Y55 Y,1Y5E A,1Y5E B,1Y5E C,1Y5H A,1Y5H B,1Y60 A,1Y60 B,1Y60 C,1Y60 D,1Y60 E,1Y63 A,1Y66 A,1Y66 B,1Y66 C,1Y66 D,1Y6I A,1Y6X A,1Y6Z A,1Y6Z B,1Y71 A,1Y71 B,1Y79 1,1Y7P A,1Y7P B,1Y7P C,1Y7R A,1Y7R B,1Y7W A,1Y7W B,1Y7Y A,1Y7Y B,1Y80 A,1Y81 A,1Y88 A,1Y89 A,1Y89 B,1Y8A A,1Y93 A,1Y96 A,1Y96 B,1Y96 C,1Y96 D,1Y9I A,1Y9I B,1Y9I C,1Y9I D,1Y9L A,1Y9Q A,1Y9U A,1Y9W A,1Y9W B,1Y9Z A,1Y9Z B,1YAC A,1YAC B,1YAR A,1YAR B,1YAR C,1YAR D,1YAR E,1YAR F,1YAR G,1YAR H,1YAR I,1YAR J,1YAR K,1YAR L,1YAR M,1YAR N,1YAR O,1YAR P,1YAR Q,1YAR R,1YAR S,1YAR T,1YAR U,1YB0 A,1YB0 B,1YB0 C,1YB1 A,1YB1 B,1YB3 A,1YB5 A,1YB5 B,1YBK A,1YBK B,1YBK C,1YBK D,1YBX A,1YBX B,1YBY A,1YBY B,1YBZ A,1YC5 A,1YC5 B,1YC9 A,1YCD A,1YCD B,1YCK A,1YD0 A,1YD6 A,1YD6 B,1YD6 C,1YD6 D,1YDG A,1YDG B,1YDG C,1YDG D,1YDG E,1YDG F,1YDG G,1YDG H,1YDY A,1YDY B,1YE8 A,1YF9 A,1YF9 B,1YF9 C,1YFQ A,1YG9 A,1YGA A,1YGA B,1YGT A,1YH2 A,1YHF A,1YHT A,1YI9 A,1YIB A,1YIX A,1YIX B,1YJ7 A,1YJ7 B,1YJ7 C,1YJ7 D,1YJO A,1YJP A,1YKD A,1YKD B,1YKI A,1YKI B,1YKI C,1YKI D,1YKW A,1YKW B,1YLE A,1YLL A,1YLL B,1YLL C,1YLL D,1YLM A,1YLM B,1YLX A,1YLX B,1YM3 A,1YMT A,1YMT B,1YN3 A,1YN3 B,1YN4 A,1YN8 A,1YN8 B,1YN8 C,1YN8 D,1YN8 E,1YN8 F,1YN9 A,1YN9 B,1YN9 C,1YNB A,1YNB B,1YNB C,1YNF A,1YNF B,1YNF C,1YNF D,1YNF E,1YNF F,1YNP A,1YNP B,1YO3 A,1YO3 B,1YO3 C,1YOA A,1YOC A,1YOC B,1YOD A,1YOZ A,1YPF A,1YPH A,1YPQ A,1YPY A,1YQ2 A,1YQ5 A,1YQB A,1YQD A,1YQE A,1YQG A,1YQH A,1YQS A,1YQT A,1YR2 A,1YRB A,1YRK A,1YRR A,1YS1 X,1YS7 A,1YSP A,1YSQ A,1YSR A,1YT3 A,1YT8 A,1YTL A,1YU0 A,1YU5 X,1YUK A,1YUM A,1YUZ A,1YVR A,1YW4 A,1YW5 A,1YW9 A,1YWF A,1YWM A,1YX1 A,1YXM A,1YYA A,1YYH A,1YZ1 A,1YZF A,1YZM A,1YZV A,1Z02 A,1Z05 A,1Z06 A,1Z08 A,1Z0N A,1Z0P A,1Z0S A,1Z0W A,1Z1S A,1Z1Y A,1Z21 A,1Z2A A,1Z2N X,1Z2U A,1Z2W A,1Z3E A,1Z3X A,1Z41 A,1Z45 A,1Z47 A,1Z4E A,1Z4R A,1Z5R A,1Z5Z A,1Z67 A,1Z6M A,1Z6N A,1Z6O A,1Z70 X,1Z72 A,1Z7A A,1Z82 A,1Z8G A,1Z8O A,1Z96 A,1Z9L A,1Z9T A,1ZA0 A,1ZA4 A,1ZAI A,1ZAR A,1ZAV A,1ZB1 A,1ZBF A,1ZCE A,1ZCH A,1ZCJ A,1ZCZ A,1ZD0 A,1ZD8 A,1ZD9 A,1ZDN A,1ZDY A,1ZEI A,1ZEJ A,1ZEL A,1ZEM A,1ZFJ A,1ZGD A,1ZGK A,1ZGX A,1ZGZ A,1ZH2 A,1ZHS A,1ZHV A,1ZHX A,1ZI8 A,1ZJC A,1ZJJ A,1ZJR A,1ZK4 A,1ZK5 A,1ZKC A,1ZKE A,1ZKI A,1ZKL A,1ZKP A,1ZL0 A,1ZLD A,1ZLJ A,1ZLM A,1ZM8 A,1ZMA A,1ZMI A,1ZMM A,1ZMO A,1ZMT A,1ZN6 A,1ZN8 A,1ZOD A,1ZOW A,1ZPS A,1ZPV A,1ZPW X,1ZQ9 A,1ZR3 A,1ZR6 A,1ZRN A,1ZRU A,1ZS9 A,1ZSQ A,1ZSW A,1ZSX A,1ZSY A,1ZT3 A,1ZTD A,1ZTH A,1ZU4 A,1ZUD 1,1ZUH A,1ZUO A,1ZUU A,1ZUY A,1ZV1 A,1ZV8 A,1ZVA A,1ZVB A,1ZVT A,1ZWY A,1ZX8 A,1ZXK A,1ZXT A,1ZXX A,1ZY4 A,1ZY7 A,1ZZ1 A,1ZZG A,1ZZK A,1ZZM A,1ZZW A,2A0B A,2A14 A,2A15 A,2A1H A,2A1I A,2A1K A,2A26 A,2A28 A,2A2C A,2A2M A,2A2N A,2A2R A,2A35 A,2A38 A,2A3M A,2A3N A,2A4A A,2A4V A,2A50 A,2A5L A,2A61 A,2A65 A,2A67

A,2A6C A,2A6S A,2A6V A,2A6Y A,2A6Z A,2A72 A,2A7L A,2A8J A,2A8N A,2A97 A,2A9D A,2A9I A,2A9S A,2AAL A,2AAN A,2AAO A,2AB0 A,2ABK A,2ABS A,2ABW A,2ACF A,2ACV A,2AE0 X,2AE2 A,2AEB A,2AEE A,2AEF A,2AEN A,2AEU A,2AEX A,2AG4 A,2AG5 A,2AGK A,2AH2 A,2AH5 A,2AH6 A,2AHF A,2AHN A,2AHU A,2AIB A,2AIO A,2AJ6 A,2AJ7 A,2AJG A,2AKF A,2AKP A,2AKZ A,2AMH A,2AML A,2AN1 A,2ANX A,2AO9 A,2AP1 A,2AP3 A,2APJ A,2APO A,2APR A,2AQ5 A,2AQJ A,2AR1 A,2AR5 A,2ARC A,2ARP A,2ARR A,2ARZ A,2AS0 A,2ASF A,2ASH A,2ASK A,2ASU A,2ATV A,2ATZ A,2AU3 A,2AUW A,2AVD A,2AVT A,2AXC A,2AXI A,2AXO A,2AXQ A,2AXW A,2AYD A,2AYH A,2AZ4 A,2AZW A,2B06 A,2B0A A,2B0C A,2B0P A,2B0T A,2B0V A,2B18 A,2B1E A,2B1K A,2B1L A,2B1M A,2B1X A,2B1Y A,2B2H A,2B3F A,2B3H A,2B3Y A,2B4H A,2B4L A,2B4V A,2B4W A,2B5A A,2B5W A,2B61 A,2B69 A,2B78 A,2B7K A,2B7U A,2B82 A,2B8I A,2B8M A,2B8T A,2B94 A,2B97 A,2B9D A,2B9E A,2B9L A,2B9W A,2BA2 A,2BAY A,2BB6 A,2BBA A,2BBE A,2BBH A,2BBR A,2BC0 A,2BC3 A,2BCE A,2BCM B,2BD0 A,2BDR A,2BEM A,2BEP A,2BEQ A,2BEZ C,2BF6 A,2BF9 A,2BFD A,2BFI A,2BFW A,2BGK A,2BGS A,2BH4 X,2BH8 A,2BHG A,2BHU A,2BI0 A,2BI7 A,2BIB A,2BII A,2BJ0 A,2BJD A,2BJF A,2BJI A,2BJK A,2BJN A,2BJQ A,2BJR A,2BJU A,2BK8 A,2BK9 A,2BKA A,2BKF A,2BKL A,2BKM A,2BKX A,2BKY A,2BL0 A,2BL8 A,2BL9 A,2BLF A,2BLN A,2BM3 A,2BM5 A,2BMO A,2BNL A,2BNM A,2BO4 A,2BOG X,2BON A,2BOU A,2BOY A,2BPD A,2BPT A,2BQ4 A,2BRF A,2BRY A,2BS2 A,2BSJ A,2BSY A,2BSZ A,2BT6 A,2BT9 A,2BTI A,2BU3 A,2BUE A,2BV2 A,2BVF A,2BW0 A,2BW3 A,2BW4 A,2BW8 A,2BWF A,2BWQ A,2BWR A,2BYC A,2BZ1 A,2BZ7 A,2BZL A,2BZV A,2C07 A,2C0A A,2C0C A,2C0G A,2C0H A,2C0N A,2C0Z A,2C1I A,2C1L A,2C1X A,2C21 A,2C29 D,2C2A A,2C2I A,2C2N A,2C2P A,2C2U A,2C2X A,2C31 A,2C3F A,2C3G A,2C3N A,2C41 A,2C42 A,2C43 A,2C46 A,2C4B A,2C4E A,2C4I A,2C4J A,2C4M A,2C4N A,2C4W A,2C4X A,2C53 A,2C5A A,2C5Q A,2C60 A,2C61 A,2C6Q A,2C6U A,2C71 A,2C78 A,2C81 A,2C8M A,2C8S A,2C92 A,2C95 A,2C9J A,2CAL A,2CAR A,2CAY A,2CB1 A,2CB5 A,2CB8 A,2CB9 A,2CBP A,2CBZ A,2CC0 A,2CC6 A,2CCM A,2CCV A,2CCY A,2CDC A,2CDN A,2CDO A,2CDU A,2CE0 A,2CE2 X,2CF7 A,2CFC A,2CFM A,2CFU A,2CG7 A,2CGQ A,2CH5 A,2CHC A,2CHO A,2CI1 A,2CI2 I,2CIA A,2CIS A,2CIU A,2CIW A,2CJ4 A,2CJG A,2CJJ A,2CJL A,2CJS A,2CJT A,2CJZ A,2CKK A,2CKL A,2CKS A,2CKX A,2CL3 A,2CM5 A,2CMG A,2CMP A,2CNQ A,2CO3 A,2COV D,2CPG A,2CS7 A,2CU3 A,2CU5 A,2CUA A,2CUK A,2CUL A,2CVB A,2CVC A,2CVD A,2CVE A,2CVI A,2CVZ A,2CW2 A,2CW5 A,2CW9 A,2CWD A,2CWQ A,2CWR A,2CWS A,2CWY A,2CWZ A,2CX1 A,2CX7 A,2CXA A,2CXC A,2CXH A,2CXI A,2CXK A,2CXX A,2CXY A,2CY2 A,2CY3 A,2CY5 A,2CYE A,2CYJ A,2CYY A,2CZ2 A,2CZ4 A,2CZD A,2CZL A,2CZQ A,2CZS A,2D0I A,2D0O A,2D1C A,2D1G A,2D1L A,2D1S A,2D1Y A,2D1Z A,2D28 C,2D29 A,2D2E A,2D2R A,2D37 A,2D39 A,2D3D A,2D3Y A,2D48 A,2D4P A,2D4U A,2D4X A,2D58 A,2D59 A,2D5B A,2D5C A,2D5F A,2D5M A,2D5W A,2D68 A,2D69 A,2D7V A,2D81 A,2D8D A,2DB7 A,2DBB A,2DBN A,2DBQ A,2DBY A,2DC0 A,2DC1 A,2DC3 A,2DC4 A,2DDF A,2DDR A,2DDX A,2DE3 A,2DE6 A,2DEB A,2DEJ A,2DFA A,2DG1 A,2DGA A,2DHO A,2DJI A,2DKA A,2DKH A,2DKJ A,2DKN A,2DKO A,2DKV A,2DLB A,2DM6 A,2DM9 A,2DOK A,2DP9 A,2DPF A,2DPL A,2DPM A,2DPQ A,2DPR A,2DQL A,2DQW A,2DR1 A,2DR3 A,2DRE A,2DRI A,2DS2 A,2DS5 A,2DSC A,2DSJ A,2DSK A,2DST A,2DSX A,2DSY A,2DT4 A,2DT8 A,2DTC A,2DTJ A,2DTR A,2DTX A,2DUL

A,2DUR A,2DUY A,2DVK A,2DVM A,2DWC A,2DWK A,2DWU A,2DXA
A,2DXQ A,2DXU A,2DY0 A,2DY1 A,2DYI A,2DYJ A,2DYK A,2DYO A,2DYU
A,2E01 A,2E0N A,2E0T A,2E11 A,2E12 A,2E1F A,2E1N A,2E1V A,2E1Z
A,2E26 A,2E2O A,2E2R A,2E3D A,2E3H A,2E3I A,2E3N A,2E3V A,2E4T
A,2E56 A,2E5F A,2E5Y A,2E6F A,2E6M A,2E6X A,2E7V A,2E7Y A,2E7Z
A,2E85 A,2E8B A,2E8E A,2E8G A,2E9L A,2E9Y A,2EA7 A,2EAB A,2EAQ
A,2EAY A,2EB1 A,2EB4 A,2EBB A,2EBF X,2EBJ A,2EBN A,2EBO A,2ECE
A,2ECU A,2ED6 A,2EEN A,2EF0 A,2EF5 A,2EF8 A,2EFJ A,2EFR A,2EFV
A,2EGD A,2EGJ A,2EGO A,2EGV A,2EGZ A,2EH3 A,2EH6 A,2EHG A,2EHH
A,2EHP A,2EHZ A,2EI5 A,2EI9 A,2EIG A,2EIX A,2EIY A,2EJ5 A,2EJ8 A,2EJ9
A,2EJA A,2EJN A,2EJW A,2EK0 A,2EK1 A,2EK8 A,2EKC A,2EKG A,2EKL
A,2EKP A,2ELA A,2ELC A,2END A,2ENG A,2EO4 A,2EO5 A,2EPI A,2EPL
X,2EQ6 A,2ERF A,2ERL A,2ERV A,2ERW A,2ES9 A,2ESS A,2ET1 A,2ETB
A,2ETJ A,2ETV A,2EU9 A,2EV1 A,2EVA A,2EVB A,2EVE A,2EW0 A,2EW2
A,2EWF A,2EWH A,2EWT A,2EX2 A,2EX4 A,2EYU A,2EZ1 A,2F01 A,2F02
A,2F0C A,2F1F A,2F1K A,2F1N A,2F22 A,2F23 A,2F2B A,2F2E A,2F2H
A,2F46 A,2F4M A,2F4P A,2F4Q A,2F4W A,2F51 A,2F5G A,2F5T X,2F5X
A,2F60 K,2F62 A,2F68 X,2F69 A,2F6E A,2F6R A,2F6U A,2F71 A,2F7B A,2F7V
A,2F8A A,2F8Y A,2F9F A,2F9H A,2F9I A,2F9L A,2F9N A,2F9W A,2FA1
A,2FA5 A,2FA8 A,2FAO A,2FAZ A,2FB5 A,2FB6 A,2FBA A,2FBD A,2FBH
A,2FBN A,2FBO J,2FBQ A,2FCF A,2FCJ A,2FCK A,2FCL A,2FCO A,2FCR
A,2FCT A,2FCW A,2FD4 A,2FD5 A,2FDN A,2FDR A,2FDV A,2FE3 A,2FE5
A,2FE7 A,2FEA A,2FEF A,2FEX A,2FF4 A,2FFU A,2FFY A,2FG1 A,2FGQ
X,2FH1 A,2FH7 A,2FHF A,2FHP A,2FHQ A,2FHT A,2FHZ A,2FI1 A,2FI9
A,2FIP A,2FIU A,2FJ8 A,2FJR A,2FK5 A,2FK8 A,2FK9 A,2FKB A,2FKK A,2FL4
A,2FLH A,2FLI A,2FM9 A,2FMA A,2FMD A,2FN0 A,2FN9 A,2FNA A,2FNO
A,2FNU A,2FO3 A,2FOM A,2FOZ A,2FP1 A,2FP7 A,2FPE A,2FPH X,2FPQ
A,2FPR A,2FQ3 A,2FQ4 A,2FQP A,2FQX A,2FR1 A,2FR2 A,2FR5 A,2FRE
A,2FRG P,2FS2 A,2FSH A,2FSJ A,2FSQ A,2FSR A,2FSU A,2FSX A,2FT0
A,2FTR A,2FTX A,2FU0 A,2FU4 A,2FUE A,2FUJ A,2FUK A,2FUL A,2FUP
A,2FUR A,2FVH A,2FVV A,2FVY A,2FW5 A,2FWH A,2FWM X,2FWT A,2FWV
A,2FX5 A,2FXQ A,2FY6 A,2FY7 A,2FYF A,2FYG A,2FYX A,2FZP A,2FZS
A,2FZV A,2G0C A,2G0I A,2G0W A,2G1U A,2G29 A,2G2C A,2G2D A,2G2N
A,2G2S A,2G30 A,2G3A A,2G3B A,2G3F A,2G3R A,2G3W A,2G40 A,2G50
A,2G5C A,2G5D A,2G5G X,2G5X A,2G62 A,2G64 A,2G6F X,2G6Y A,2G76
A,2G7B A,2G7C A,2G7O A,2G7S A,2G84 A,2G8S A,2G9W A,2G9Z A,2GA1
A,2GA8 A,2GAG A,2GAI A,2GAK A,2GAS A,2GAU A,2GAX A,2GB4 A,2GBW
A,2GCU A,2GDM A,2GDQ A,2GDZ A,2GE7 A,2GEC A,2GEN A,2GEY A,2GF0
A,2GF3 A,2GF6 A,2GFF A,2GFH A,2GFN A,2GFO A,2GFQ A,2GG6 A,2GGC
A,2GGO A,2GGS A,2GH9 A,2GHA A,2GHS A,2GHT A,2GI3 A,2GIA A,2GIB
A,2GIY A,2GJ3 A,2GJ4 A,2GJ8 A,2GJL A,2GK4 A,2GKE A,2GKG A,2GKM
A,2GKP A,2GL5 A,2GL9 A,2GLZ A,2GMQ A,2GMW A,2GMY A,2GN0 A,2GN4
A,2GNC A,2GNO A,2GNP A,2GOM A,2GOP A,2GPE A,2GPI A,2GPY A,2GQ0
A,2GQ1 A,2GQR A,2GQT A,2GQW A,2GR8 A,2GRC A,2GS4 A,2GS5 A,2GS8
A,2GSO A,2GSV A,2GT1 A,2GTR A,2GU1 A,2GU3 A,2GU9 A,2GUD A,2GUF
A,2GUH A,2GUI A,2GUK A,2GUX A,2GUZ A,2GVI A,2GVK A,2GW4 A,2GWG
A,2GWM A,2GWN A,2GXG A,2GXQ A,2GYQ A,2GYZ A,2GZ4 A,2GZ6 A,2GZB
A,2GZQ A,2GZS A,2GZV A,2H00 A,2H0U A,2H1C A,2H1R A,2H1T A,2H1V
A,2H29 A,2H2B A,2H2T B,2H30 A,2H3G X,2H3H A,2H3L A,2H4P A,2H4V
A,2H57 A,2H58 A,2H5C A,2H6D A,2H6E A,2H6F A,2H6L A,2H6U A,2H7C

A,2H7O A,2H7W A,2H88 A,2H8E A,2H8G A,2H8L A,2H8O A,2H98 A,2H9A A,2H9U A,2HA8 A,2HAL A,2HAZ A,2HBA A,2HBG A,2HBO A,2HBV A,2HBW A,2HC1 A,2HC8 A,2HC9 A,2HCF A,2HCM A,2HD9 A,2HDO A,2HDV A,2HDW A,2HDZ A,2HEK A,2HES X,2HEU A,2HEW F,2HF1 A,2HF2 A,2HF9 A,2HFK A,2HFN A,2HFS A,2HHC A,2HHG A,2HHP A,2HHZ A,2HI0 A,2HI4 A,2HIN A,2HIQ A,2HIY A,2HJE A,2HJH A,2HJN A,2HJP A,2HJV A,2HKE A,2HKJ A,2HKU A,2HKV A,2HL7 A,2HLC A,2HLJ A,2HLR A,2HLS A,2HLV A,2HLY A,2HM7 A,2HMH A,2HMZ A,2HNF A,2HNG A,2HNL A,2HNU A,2HO1 A,2HO2 A,2HOQ A,2HOX A,2HPG A,2HPJ A,2HPS A,2HPV A,2HPW A,2HQ4 A,2HQ7 A,2HQ9 A,2HQH A,2HQL A,2HQQ A,2HQS A,2HQT A,2HQV A,2HQX A,2HQY A,2HRA A,2HRV A,2HRZ A,2HS1 A,2HSA B,2HSB A,2HSI A,2HSJ A,2HSZ A,2HT9 A,2HTA A,2HTD A,2HTS A,2HU9 A,2HUF A,2HUH A,2HUJ A,2HUO A,2HVW A,2HW2 A,2HW4 A,2HWV A,2HX0 A,2HX5 A,2HXI A,2HXP A,2HXS A,2HXV A,2HY1 A,2HY5 A,2HY7 A,2HYK A,2HYT A,2HYX A,2HZC A,2HZF A,2HZL A,2HZQ A,2I02 A,2I0K A,2I0O A,2I0Z A,2I24 N,2I2C A,2I2O A,2I2W A,2I33 A,2I3D A,2I3H A,2I49 A,2I4L A,2I4S A,2I51 A,2I53 A,2I5F A,2I5H A,2I5I A,2I5U A,2I5V O,2I61 A,2I6D A,2I6H A,2I6J A,2I6V A,2I71 A,2I74 A,2I7A A,2I7C A,2I7F A,2I7G A,2I87 A,2I8B A,2I8D A,2I8T A,2I9A A,2I9C A,2I9F A,2I9I A,2I9W A,2I9X A,2IA1 A,2IA7 A,2IAB A,2IAI A,2IAY A,2IB0 A,2IB8 A,2IBD A,2IBL A,2IBN A,2IBP A,2IC2 A,2IC6 A,2IC9 A,2ICG A,2ICH A,2ICI A,2ICT A,2ICU A,2ICY A,2ID3 A,2ID4 A,2ID6 A,2IDL A,2IDR A,2IEA A,2IEL A,2IEQ A,2IEW A,2IF5 A,2IF6 A,2IFC A,2IFR A,2IFX A,2IG6 A,2IG8 A,2IGI A,2IGP A,2IGT A,2IGX A,2IHD A,2IHT A,2IHY A,2II1 A,2II2 A,2IIA A,2IID A,2IJ2 A,2IJA A,2IJQ A,2IKB A,2IKK A,2IKS A,2ILR A,2IM8 A,2IM9 A,2IMF A,2IMH A,2IMI A,2IMJ A,2IML A,2IMQ X,2IMR A,2IMS A,2IMZ A,2IN0 A,2IN3 A,2INU A,2INW A,2ION A,2IP1 A,2IP2 A,2IP6 A,2IPI A,2IQJ A,2IQY A,2IRU A,2IS8 A,2IS9 A,2ISB A,2ISM A,2ISN A,2ISW A,2ISY A,2IT2 A,2IT9 A,2IU1 A,2IU4 A,2IU5 A,2IUH A,2IUM A,2IUW A,2IVF A,2IVN A,2IVX A,2IVY A,2IW0 A,2IW1 A,2IW2 A,2IWA A,2IWB A,2IWK A,2IWN A,2IWQ A,2IWR A,2IXC A,2IXD A,2IXE A,2IXS A,2IXT A,2IY2 A,2IY9 A,2IYA A,2IYJ A,2IYV A,2IZ6 A,2IZQ A,2IZR A,2IZX A,2IZZ A,2J05 A,2J07 A,2J0A A,2J0V A,2J13 A,2J1A A,2J1P A,2J1V A,2J23 A,2J2J A,2J32 A,2J3X A,2J43 A,2J4D A,2J4X A,2J5Y A,2J66 A,2J6A A,2J6B A,2J6F A,2J6L A,2J6V A,2J6Y A,2J73 A,2J7J A,2J7T A,2J82 A,2J89 A,2J8B A,2J8G A,2J8H A,2J8K A,2J8M A,2J8W A,2J91 A,2J97 A,2J9O A,2J9U A,2J9W A,2JA2 A,2JAE A,2JAF A,2JAH A,2JAM A,2JAY A,2JBM A,2JBV A,2JC4 A,2JC5 A,2JC9 A,2JCB A,2JD4 A,2JDA A,2JDC A,2JDI A,2JDJ A,2JE3 A,2JE6 A,2JE8 A,2JEK A,2JEN A,2JEP A,2JER A,2JFN A,2JFR A,2JG0 A,2JG6 A,2JGB A,2JGN A,2JGP A,2JGS A,2JH1 A,2JH3 A,2JHF A,2JHN A,2JIF A,2JIG A,2JII A,2JIK A,2JIL A,2JIS A,2JJN A,2JJQ A,2JJU A,2JKB A,2JKG A,2JKS A,2JKU A,2JL1 A,2JLI A,2JLJ A,2JLP A,2JLQ A,2KIN A,2LHB A,2LIG A,2LIS A,2LTN A,2MCM A,2MHR A,2MLT A,2MSB A,2NAC A,2NLR A,2NLS A,2NLV A,2NML A,2NN5 A,2NNU A,2NO4 A,2NOG A,2NP5 A,2NPN A,2NPT A,2NQ3 A,2NQ5 A,2NQL A,2NQT A,2NQW A,2NR4 A,2NR5 A,2NR7 A,2NRK A,2NRR A,2NRT A,2NS9 A,2NSA A,2NSF A,2NSZ A,2NT0 A,2NT8 A,2NTE A,2NTP A,2NUH A,2NUJ A,2NV0 A,2NVA A,2NVO A,2NW2 A,2NW8 A,2NWF A,2NWH A,2NWV A,2NX2 A,2NX4 A,2NX8 A,2NX9 A,2NXC A,2NXF A,2NXV A,2NXW A,2NYC A,2NYI A,2NYN A,2NYU A,2NZ7 A,2NZC A,2NZL A,2NZX A,2O04 A,2O07 A,2O08 A,2O0A A,2O0B A,2O0J A,2O0M A,2O0Q A,2O0R A,2O0Y A,2O16 A,2O1A A,2O1B A,2O1C A,2O1K A,2O1Q A,2O20 A,2O23 A,2O28 A,2O2G A,2O2K A,2O2P A,2O2X A,2O30 A,2O34 A,2O36

A,2O38 A,2O3F A,2O4D A,2O4J A,2O4T A,2O4V A,2O4X A,2O57 A,2O5F A,2O5H A,2O5U A,2O5V A,2O62 A,2O66 A,2O6F A,2O6L A,2O6N A,2O6P A,2O6X A,2O6Y A,2O70 A,2O71 A,2O7A A,2O7I A,2O7M A,2O7R A,2O7S A,2O8L A,2O8N A,2O8P A,2O8Q A,2O90 A,2O99 A,2O9G A,2O9S A,2O9U X,2OA2 A,2OA9 A,2OAF A,2OAI A,2OB0 A,2OB3 A,2OB5 A,2OBI A,2OBL A,2OBP A,2OC3 A,2OC6 A,2OCG A,2OCH A,2OCT A,2OCZ A,2OD0 A,2OD4 A,2OD5 A,2OD6 A,2ODA A,2ODF A,2ODH A,2ODK A,2ODL A,2ODP A,2OE3 A,2OEB A,2OEE A,2OEM A,2OER A,2OEZ A,2OF3 A,2OFC A,2OFK A,2OFY A,2OFZ A,2OG2 A,2OG4 A,2OG5 A,2OG9 A,2OGF A,2OGI A,2OGT A,2OH1 A,2OH3 A,2OHH A,2OHW A,2OIB A,2OIF A,2OIK A,2OIT A,2OIW A,2OIX A,2OIZ D,2OJ6 A,2OJH A,2OKF A,2OKG A,2OKM A,2OKQ A,2OKT A,2OKU A,2OKV A,2OKX A,2OKZ A,2OL9 A,2OLG A,2OLM A,2OLN A,2OLR A,2OLT A,2OLW A,2OLX A,2OMD A,2OMK A,2OML A,2OMM A,2OMO A,2OMP A,2OMQ A,2ON9 A,2ONF A,2ONV A,2ONW X,2ONX A,2OO2 A,2OO3 A,2OOA A,2OOC A,2OOJ A,2OOK A,2OOQ A,2OPC A,2OPG A,2OPJ A,2OPO A,2OPW A,2OQ0 A,2OQ1 A,2OQ5 A,2OQB A,2OQG A,2OQK A,2OQM A,2OQQ A,2OQZ A,2OR7 A,2ORD A,2ORW A,2OS0 A,2OS5 A,2OSA A,2OSO A,2OSV A,2OSX A,2OT9 A,2OTM A,2OU1 A,2OU3 A,2OU5 A,2OU6 A,2OUS A,2OUW A,2OV0 A,2OV9 A,2OVG A,2OVJ A,2OVS A,2OWA A,2OWN A,2OWP A,2OX6 A,2OX7 A,2OX9 A,2OXC A,2OXG Z,2OXL A,2OXO A,2OY7 A,2OY9 A,2OYA A,2OYC A,2OYO A,2OYR A,2OYZ A,2OZE A,2OZG A,2OZH A,2OZJ A,2OZN A,2OZT A,2OZV A,2P02 A,2P08 A,2P09 A,2P0A A,2P0B A,2P0D A,2P0K A,2P0N A,2P0S A,2P0W A,2P12 A,2P13 A,2P14 A,2P17 A,2P18 A,2P1M A,2P1T A,2P23 A,2P25 A,2P26 A,2P2O A,2P2S A,2P2V A,2P2W A,2P35 A,2P38 A,2P39 A,2P3E A,2P3H A,2P3P A,2P3W A,2P3Y A,2P4E P,2P4F A,2P4H X,2P4O A,2P4P A,2P51 A,2P57 A,2P58 A,2P5K A,2P5M A,2P5Q A,2P5V A,2P5X A,2P5Y A,2P65 A,2P67 A,2P6H A,2P6V A,2P6W A,2P6X A,2P6Y A,2P6Z A,2P7I A,2P7O A,2P84 A,2P8B A,2P8E A,2P8G A,2P8I A,2P8J A,2P8T A,2P92 A,2P97 A,2P9B A,2P9H A,2P9W A,2P9X A,2PA1 A,2PA4 A,2PA7 A,2PA8 D,2PAG A,2PB7 A,2PBC A,2PBF A,2PBI A,2PBK A,2PBL A,2PBP A,2PBR A,2PC1 A,2PCJ A,2PCN A,2PD1 A,2PD6 A,2PDR A,2PE4 A,2PE8 A,2PEB A,2PEF A,2PEQ A,2PET A,2PF5 A,2PFB A,2PFI A,2PFW A,2PFX A,2PFY A,2PFZ A,2PG0 A,2PGE A,2PGF A,2PGO A,2PGW A,2PH0 A,2PHN A,2PI2 A,2PIA A,2PIE A,2PIJ A,2PJS A,2PJZ A,2PK3 A,2PK8 A,2PKE A,2PKF A,2PKH A,2PL1 A,2PLC A,2PLI A,2PLJ A,2PLN A,2PLR A,2PLW A,2PMA A,2PMK A,2PMU A,2PN0 A,2PN1 A,2PN2 A,2PN6 A,2PN8 A,2PND A,2PNE A,2PNQ A,2PNW A,2PO4 A,2POC A,2POF A,2POI A,2POK A,2POR A,2POS A,2PPN A,2PPQ A,2PPT A,2PPV A,2PPX A,2PQ3 A,2PQ7 A,2PQ8 A,2PQQ A,2PQV A,2PQX A,2PR5 A,2PR7 A,2PRD A,2PRV A,2PRX A,2PS1 A,2PS2 A,2PSD A,2PSP A,2PST X,2PT6 A,2PTT A,2PU3 A,2PU9 A,2PUL A,2PUY A,2PUZ A,2PV2 A,2PV4 A,2PV7 A,2PVB A,2PVQ A,2PVU A,2PW0 A,2PWA A,2PWQ A,2PWW A,2PWY A,2PXX A,2PY2 A,2PYQ A,2PYT A,2PYW A,2PYX A,2PZ0 A,2PZE A,2PZH A,2PZM A,2Q03 A,2Q09 A,2Q0D A,2Q0L A,2Q0O A,2Q0S A,2Q0T A,2Q0Y A,2Q0Z X,2Q12 A,2Q1S A,2Q24 A,2Q2F A,2Q2G A,2Q2H A,2Q30 A,2Q35 A,2Q3E A,2Q3G A,2Q3M A,2Q3P A,2Q3T A,2Q3V A,2Q3W A,2Q3X A,2Q3Z A,2Q40 A,2Q43 A,2Q46 A,2Q48 A,2Q4F A,2Q4H A,2Q4M A,2Q4O A,2Q4V A,2Q4W A,2Q4Z A,2Q52 A,2Q5C A,2Q5W E,2Q5X A,2Q62 A,2Q6F A,2Q6K A,2Q6Q A,2Q73 A,2Q79 A,2Q7B A,2Q7D A,2Q7S A,2Q7V A,2Q7W A,2Q7X A,2Q82 A,2Q87 A,2Q88 A,2Q8K A,2Q8N A,2Q8P A,2Q8R E,2Q8X A,2Q99

A,2Q9K A,2Q9O A,2Q9R A,2Q9U A,2Q9V A,2QA1 A,2QAP A,2QB7 A,2QBW
A,2QCK A,2QCP X,2QCU A,2QCV A,2QDE A,2QDJ A,2QE6 A,2QE8 A,2QE9
A,2QEB A,2QEC A,2QED A,2QEE A,2QEU A,2QF4 A,2QF7 A,2QF9 A,2QFA
A,2QFE A,2QFF A,2QFL A,2QG1 A,2QG3 A,2QG8 A,2QGM A,2QGQ A,2QGS
A,2QGU A,2QGY A,2QH9 A,2QHF A,2QHK A,2QHL A,2QHP A,2QHQ A,2QHS
A,2QHT A,2QIB A,2QIF A,2QIH A,2QIK A,2QIP A,2QIW A,2QIY A,2QJ2
A,2QJ8 A,2QJL A,2QJV A,2QJW A,2QJX A,2QJZ A,2QK1 A,2QKD A,2QKH
B,2QKP A,2QKV A,2QL8 A,2QLT A,2QLW A,2QLX A,2QM0 A,2QM6 A,2QM8
A,2QMA A,2QML A,2QMM A,2QMQ A,2QN0 A,2QN4 A,2QND A,2QNG A,2QNI
A,2QNK A,2QNL A,2QNT A,2QO4 A,2QOL A,2QOR A,2QP2 A,2QPN A,2QPQ
A,2QPW A,2QPX A,2QPZ A,2QQ4 A,2QQ5 A,2QQ8 A,2QQI A,2QQM A,2QQY
A,2QQZ A,2QR3 A,2QR6 A,2QR7 A,2QRR A,2QRT A,2QRU A,2QRW A,2QS9
A,2QSA A,2QSB A,2QSI A,2QSK A,2QSQ A,2QSW A,2QSX A,2QT1 A,2QTD
A,2QTF A,2QTQ A,2QTS A,2QTZ A,2QUD A,2QUL A,2QUO A,2QUP A,2QUY
A,2QV5 A,2QV6 A,2QVE A,2QVG A,2QVK A,2QW5 A,2QWU A,2QX2 A,2QXI
A,2QXX A,2QXY A,2QY1 A,2QY6 A,2QYC A,2QYW A,2QZC A,2QZQ A,2QZT
A,2QZU A,2R01 A,2R09 A,2R0B A,2R0C A,2R0H A,2R0X A,2R0Y A,2R16
A,2R1I A,2R25 A,2R2A A,2R2C A,2R2D A,2R2I A,2R2N A,2R2O A,2R2Y
A,2R2Z A,2R31 A,2R32 A,2R37 A,2R3A A,2R3B A,2R44 A,2R47 A,2R4F
A,2R4G A,2R4I A,2R4Q A,2R58 A,2R5O A,2R5T A,2R5U A,2R60 A,2R6J
A,2R6O A,2R6Q A,2R6U A,2R6V A,2R6Z A,2R75 1,2R78 A,2R7D A,2R7G
A,2R7H A,2R85 A,2R8E A,2R8O A,2R8Q A,2R8U A,2R8W A,2R91 A,2R9F
A,2RA4 A,2RA6 A,2RA8 A,2RA9 A,2RAF A,2RAG A,2RAS A,2RAU A,2RB7
A,2RB8 A,2RB9 A,2RBB A,2RBC A,2RBD A,2RBG A,2RBK A,2RC3 A,2RC8
A,2RCC A,2RCI A,2RCZ A,2RDC A,2RDE A,2RDG A,2RDI A,2RDM A,2RDQ
A,2RDX A,2RDZ A,2RE2 A,2REE A,2REK A,2REM A,2REU A,2REY A,2RF0
A,2RFA A,2RFF A,2RFM A,2RFQ A,2RFR A,2RFV A,2RG4 A,2RG8 A,2RGQ
A,2RH0 A,2RH2 A,2RH3 A,2RH7 A,2RHF A,2RHK A,2RHM A,2RHW A,2RI0
A,2RI7 A,2RI9 A,2RIE A,2RIJ A,2RIK A,2RIL A,2RIN A,2RIQ A,2RJ2 A,2RJI
A,2RK3 A,2RK5 A,2RK9 A,2RKH A,2RKL A,2RKN A,2RKQ A,2RKV A,2RKY
A,2RL8 A,2RLD A,2RSP A,2SAK A,2SCP A,2SHP A,2SLI A,2SN3 A,2SPC
A,2SQC A,2TGI A,2TNF A,2TPS A,2UU8 A,2UUQ A,2UUR A,2UUU A,2UV4
A,2UVK A,2UVO A,2UWA A,2UWI A,2UX9 A,2UXT A,2UXW A,2UY2 A,2UYO
A,2UYT A,2UZ0 A,2V03 A,2V05 A,2V09 A,2V0C A,2V0H A,2V0P A,2V0U
A,2V1M A,2V1O A,2V1Q A,2V1X A,2V25 A,2V27 A,2V2F A,2V2G A,2V2K
A,2V33 A,2V3G A,2V3I A,2V3S A,2V3U A,2V3V A,2V3Z A,2V40 A,2V4B
A,2V4N A,2V4V A,2V4X A,2V57 A,2V5M A,2V5T A,2V62 A,2V6K A,2V6U
A,2V6V A,2V6X A,2V75 A,2V76 A,2V78 A,2V79 A,2V7F A,2V7K A,2V7S
A,2V84 A,2V89 A,2V8F A,2V8H A,2V8I A,2V8T A,2V94 A,2V9K A,2V9L
A,2V9R A,2V9T A,2V9V A,2VAC A,2VAP A,2VB1 A,2VBF A,2VBK A,2VBU
A,2VC8 A,2VCH A,2VCL A,2VCN A,2VD8 A,2VDF A,2VDJ A,2VE8 A,2VEC
A,2VEF A,2VEZ A,2VFK A,2VFO A,2VFR A,2VFX A,2VG0 A,2VG9 A,2VGA
A,2VH7 A,2VHA A,2VHJ A,2VHK A,2VIF A,2VIM A,2VJW A,2VK2 A,2VK8
A,2VKJ A,2VKL A,2VKP A,2VLG A,2VLI A,2VLQ A,2VM5 A,2VM9 A,2VMH
A,2VN4 A,2VN6 A,2VNF A,2VNG A,2VNL A,2VO8 A,2VO9 A,2VOF A,2VOK
A,2VOS A,2VOV A,2VOZ A,2VPA A,2VPB A,2VPH A,2VPJ A,2VPK A,2VPN
A,2VPT A,2VQ2 A,2VQ3 A,2VQ9 A,2VQG A,2VQM A,2VQP A,2VQR A,2VQX
A,2VRI A,2VRQ A,2VRS A,2VS0 A,2VSD A,2VSV A,2VT1 A,2VT3 A,2VTC
A,2VTF A,2VTW A,2VU9 A,2VUN A,2VUV A,2VVE A,2VVG A,2VVK A,2VVM
A,2VVP A,2VVW A,2VW8 A,2VWR A,2VWS A,2VXG A,2VXN A,2VXR A,2VXZ

A,2VY8 A,2VYO A,2VYW A,2VZ5 A,2VZC A,2VZP A,2VZS A,2VZY A,2W0B A,2W0G A,2W0I A,2W0M A,2W0P A,2W15 A,2W18 A,2W1J A,2W1N A,2W1R A,2W1S A,2W1V A,2W20 A,2W2A A,2W2J A,2W2R A,2W31 A,2W39 A,2W3G A,2W3J A,2W3P A,2W3Q A,2W3X A,2W3Y A,2W3Z A,2W40 A,2W43 A,2W47 A,2W4E A,2W4F A,2W50 A,2W53 A,2W59 A,2W5A A,2W5E A,2W5F A,2W5Q A,2W5W A,2W5Y A,2W61 A,2W6A A,2W6K A,2W7A A,2W7Q A,2W7Z A,2W86 A,2W87 A,2W8N A,2W8T A,2W8X A,2W91 A,2W9H A,2W9X A,2W9Y A,2WA7 A,2WAA A,2WAG A,2WAN A,2WAO A,2WAW A,2WB0 X,2WB3 A,2WB6 A,2WB9 A,2WBF X,2WBM A,2WBN A,2WBQ A,2WBX A,2WCE A,2WCI A,2WCJ A,2WCO A,2WCR A,2WCU A,2WCW A,2WDC A,2WDS A,2WE5 A,2WEI A,2WEU A,2WF7 A,2WFB A,2WFH A,2WFI A,2WFO A,2WFP A,2WFV A,2WFW A,2WG7 A,2WGK A,2WGP A,2WH7 A,2WHL A,2WI8 A,2WIY A,2WJ5 A,2WJ9 A,2WJE A,2WJN C,2WJR A,2WK1 A,2WKJ A,2WKK A,2WKQ A,2WKX A,2WL1 A,2WLC A,2WLR A,2WLU A,2WM3 A,2WM5 A,2WM8 A,2WMF A,2WN3 A,2WN4 A,2WN9 A,2WNF A,2WNH A,2WNK A,2WNP F,2WNS A,2WNW A,2WNX A,2WNY A,2WO1 A,2WOE A,2WOJ A,2WOL A,2WOY A,2WP7 A,2WPG A,2WPH E,2WPV A,2WQ4 A,2WQF A,2WQI A,2WQK A,2WQR A,2WR8 A,2WRA A,2WRY A,2WSB A,2WSD A,2WSH A,2WT0 A,2WTA A,2WTE A,2WTG A,2WTM A,2WTP A,2WU9 A,2WUG A,2WUJ A,2WUQ A,2WUR A,2WUX A,2WV3 A,2WVF A,2WVI A,2WVQ A,2WW4 A,2WW5 A,2WW6 A,2WW8 A,2WWE A,2WXF A,2WXU A,2WY3 A,2WY4 A,2WYA A,2WYH A,2WYQ A,2WZ1 A,2WZ8 A,2WZ9 A,2WZB A,2WZM A,2WZO A,2WZV A,2X02 A,2X0K A,2X0Q A,2X1B A,2X1D A,2X26 A,2X2S A,2X2U A,2X32 A,2X36 A,2X3C A,2X3E A,2X3G A,2X3H A,2X3J A,2X3L A,2X3M A,2X3N A,2X46 A,2X49 A,2X4D A,2X4J A,2X4K A,2X4L A,2X55 A,2X5C A,2X5F A,2X5H A,2X5N A,2X5O A,2X5P A,2X5R A,2X5X A,2X5Y A,2X61 A,2X6U A,2X6W A,2X78 A,2X7K A,2X7M A,2X7Q A,2X7R A,2X8H A,2X8S A,2X8X X,2X98 A,2X9G A,2X9O A,2X9X A,2X9Z A,2XAU A,2XB0 X,2XB4 A,2XBG A,2XBK A,2XBL A,2XBT A,2XBU A,2XC1 A,2XCI A,2XCJ A,2XCZ A,2XD3 A,2XDC A,2XDG A,2XDH A,2XDJ A,2XDP A,2XDW A,2XE4 A,2XED A,2XEP A,2XET A,2XEU A,2XEV A,2XEX A,2XF7 A,2XFD A,2XFN A,2XFR A,2XFV A,2XG5 A,2XGR A,2XGT A,2XGU A,2XGV A,2XHA A,2XHF A,2XHG A,2XHN A,2XI8 A,2XI9 A,2XIG A,2XIJ A,2XIO A,2XJ4 A,2XJH A,2XJI A,2XJP A,2XKI A,2XKR A,2XLG A,2XM5 A,2XME A,2XMI A,2XMJ A,2XMO A,2XMX A,2XMZ A,2XN2 A,2XN6 A,2XNQ A,2XOC A,2XOD A,2XOL A,2XOM A,2XOT A,2XOV A,2XPP A,2XPW A,2XQ0 A,2XQH A,2XQO A,2XQQ A,2XQU A,2XR6 A,2XRH A,2XRW A,2XRY A,2XSA A,2XSE A,2XSF A,2XSG A,2XSK A,2XSQ A,2XST A,2XSU A,2XSW A,2XT2 A,2XTL A,2XTP A,2XTS A,2XTY A,2XU3 A,2XU8 A,2XU9 A,2XUA A,2XUS A,2XUV A,2XVE A,2XVM A,2XVS A,2XVY A,2XW6 A,2XW7 A,2XW9 A,2XWL A,2XWP A,2XWS A,2XWV A,2XWX A,2XXL A,2XXN A,2XXP A,2XXZ A,2XY1 A,2XY2 A,2XYI A,2XZ2 A,2XZ4 A,2XZ8 A,2XZ9 A,2XZE A,2XZG A,2XZI A,2Y08 A,2Y0C A,2Y0O A,2Y0T A,2Y1B A,2Y1E A,2Y1N A,2Y24 A,2Y27 A,2Y28 A,2Y2A A,2Y2M A,2Y2Z A,2Y32 A,2Y3C A,2Y3J A,2Y3K A,2Y3V A,2Y3W A,2Y43 A,2Y44 A,2Y4R A,2Y4Y A,2Y53 A,2Y5C A,2Y5M A,2Y5P A,2Y5S A,2Y6N A,2Y6U A,2Y6X A.

Supplementary Table S12B. Transmembrane proteins used to compute maximal cliques, size-order and spherical coordinates.

1A0S P,1A0S Q,1A0S R,1A0T P,1A0T Q,1A0T R,2A06 C,2A06 D,2A06 E,2A06 G,2A06 P,2A06 Q,2A06 R,2A06 T,2A06 W,2A0D A,3A0B A,3A0B B,3A0B C,3A0B D,3A0B E,3A0B F,3A0B H,3A0B I,3A0B J,3A0B K,3A0B L,3A0B M,3A0B T,3A0B X,3A0B Y,3A0B N,3A0B Z,3A0B A,3A0B B,3A0B C,3A0B D,3A0B E,3A0B F,3A0B H,3A0B I,3A0B J,3A0B K,3A0B L,3A0B M,3A0B T,3A0B X,3A0B Y,3A0B N,3A0B Z,3A0H A,3A0H B,3A0H C,3A0H D,3A0H E,3A0H F,3A0H H,3A0H I,3A0H J,3A0H K,3A0H L,3A0H M,3A0H T,3A0H X,3A0H Y,3A0H N,3A0H Z,3A0H A,3A0H B,3A0H C,3A0H D,3A0H E,3A0H F,3A0H H,3A0H I,3A0H J,3A0H K,3A0H L,3A0H M,3A0H T,3A0H X,3A0H Y,3A0H N,3A0H Z,4A01 A,4A01 B,1A11 A,3A2S X,3A2S A,3A2S B,4A2N B,5A2N A,5A2N B,5A2O A,5A2O B,3A3Y A,3A3Y B,3A3Y G,4A4M A,4A4M C,5A40 A,5A40 B,5A41 A,5A41 B,5A43 A,5A43 B,5A44 A,5A44 B,5A44 C,5A45 A,5A45 B,5A45 C,2A65 A,2A65 B,5A63 A,5A63 B,5A63 C,5A63 D,5A6E A,5A6E B,5A6G A,5A6G B,5A6U A,5A6U B,5A6U G,3A7K A,3A7K B,3A7K D,4A79 A,4A7A A,4A82 A,4A82 B,5A8E A,1A91 A,2A95 B,2A9H A,2A9H B,2A9H C,2A9H D,4A97 A,4A97 B,4A97 C,4A97 D,4A97 E,4A98 A,4A98 B,4A98 C,4A98 D,4A98 E,2ABM A,2ABM B,2ABM C,2ABM D,3ABK A,3ABK B,3ABK C,3ABK D,3ABK G,3ABK I,3ABK J,3ABK K,3ABK L,3ABK M,3ABK N,3ABK O,3ABK P,3ABK Q,3ABK T,3ABK V,3ABK W,3ABK X,3ABK Y,3ABK Z,3ABL A,3ABL B,3ABL C,3ABL D,3ABL G,3ABL I,3ABL J,3ABL K,3ABL L,3ABL M,3ABL N,3ABL O,3ABL P,3ABL Q,3ABL T,3ABL V,3ABL W,3ABL X,3ABL Y,3ABL Z,3ABM A,3ABM B,3ABM C,3ABM D,3ABM G,3ABM I,3ABM J,3ABM K,3ABM L,3ABM M,3ABM N,3ABM O,3ABM P,3ABM Q,3ABM T,3ABM V,3ABM W,3ABM X,3ABM Y,3ABM Z,3ABV C,3ABV D,3ABW A,3ABW B,3ABW D,5ABB A,5ABB B,5ABB Z,2AC6 A,2ACZ C,2ACZ D,2ACZ G,2ACZ H,2ACZ K,2ACZ L,4AC5 H,4AC5 L,4AC5 M,2AD0 C,2AD0 D,3AE1 C,3AE1 D,3AE2 C,3AE2 D,3AE3 C,3AE3 D,3AE4 C,3AE4 D,3AE5 C,3AE5 D,3AE6 C,3AE6 D,3AE7 C,3AE7 D,3AE8 C,3AE8 D,3AE9 C,3AE9 D,3AEA C,3AEA D,3AEB C,3AEB D,3AEC C,3AEC D,3AED C,3AED D,3AEE C,3AEE D,3AEF C,3AEF D,3AEG C,3AEG D,3AEH A,3AEH B,5AEX A,5AEX B,5AEX C,5AEZ A,5AEZ B,5AEZ C,1AF6 A,1AF6 B,1AF6 C,1AFO A,1AFO B,2AFL A,2AFL B,2AFL C,2AFL D,4AFK A,5AF1 A,5AF1 C,5AF1 D,2AGV A,3AG1 A,3AG1 B,3AG1 C,3AG1 D,3AG1 G,3AG1 I,3AG1 J,3AG1 K,3AG1 L,3AG1 M,3AG1 N,3AG1 O,3AG1 P,3AG1 Q,3AG1 T,3AG1 V,3AG1 W,3AG1 X,3AG1 Y,3AG1 Z,3AG2 A,3AG2 B,3AG2 C,3AG2 D,3AG2 G,3AG2 I,3AG2 J,3AG2 K,3AG2 L,3AG2 M,3AG2 N,3AG2 O,3AG2 P,3AG2 Q,3AG2 T,3AG2 V,3AG2 W,3AG2 X,3AG2 Y,3AG2 Z,3AG3 A,3AG3 B,3AG3 C,3AG3 D,3AG3 G,3AG3 I,3AG3 J,3AG3 K,3AG3 L,3AG3 M,3AG3 N,3AG3 O,3AG3 P,3AG3 Q,3AG3 T,3AG3 V,3AG3 W,3AG3 X,3AG3 Y,3AG3 Z,3AG4 A,3AG4 B,3AG4 C,3AG4 D,3AG4 G,3AG4 I,3AG4 J,3AG4 K,3AG4 L,3AG4 M,3AG4 N,3AG4 O,3AG4 P,3AG4 Q,3AG4 T,3AG4 V,3AG4 W,3AG4 X,3AG4 Y,3AG4 Z,4AGE A,4AGE B,4AGE C,4AGE D,4AGE E,4AGE F,4AGE G,4AGF A,4AGF B,4AGF C,4AGF D,4AGF E,4AGF F,4AGF G,2AH3 A,2AHY A,2AHY B,2AHY C,2AHY D,2AHZ A,2AHZ B,2AHZ C,2AHZ D,5AHY A,5AHY B,5AHY C,5AHZ A,5AHZ B,5AHZ C,7AHL A,7AHL B,7AHL C,7AHL D,7AHL E,7AHL F,7AHL G,1AIG L,1AIG M,1AIG H,1AIJ L,1AIJ M,1AIJ H,4AIN A,4AIN B,4AIN C,4AIP A,4AIP B,4AIP C,4AIQ A,5AJI A,5AJI B,5AJI C,5AJI D,5AJI E,5AJI F,5AJI G,2AKH X,2AKH Y,2AKH Z,2AKH A,2AKH B,2AKH C,2AKI X,2AKI Y,2AKI Z,2AKI A,2AKI B,2AKI C,4AL0 A,4AL0 B,4AL0 C,4AL1 A,4AL1 B,4AL1 C,2AMK A,3AM6 A,3AM6 B,3AM6 C,3AM6 D,4AMI A,4AMJ A,3ANZ A,3ANZ B,3ANZ C,3ANZ D,3ANZ E,3ANZ

F,3ANZ G,5AN8 A,5AN8 B,5AN8 C,5AN8 D,3AOA A,3AOA B,3AOA C,3AOB A,3AOB B,3AOB C,3AOC A,3AOC B,3AOC C,3AOD A,3AOD B,3AOD C,3AOU A,3AOU B,3AOU C,3AOU D,3AOU E,3AOU F,3AOU G,3AOU H,3AOU I,3AOU J,1AP9 A,1AP9 B,1AP9 C,4APS A,3AQP A,4AQ5 A,4AQ5 B,4AQ5 C,4AQ5 D,4AQ5 E,4AQ9 A,4AQ9 B,4AQ9 C,4AQ9 D,4AQ9 E,1AR1 A,1AR1 B,3AR2 A,3AR3 A,3AR4 A,3AR5 A,3AR6 A,3AR7 A,3AR8 A,3AR9 A,5ARA J,5ARA K,5ARA L,5ARA M,5ARA N,5ARA O,5ARA P,5ARA Q,5ARA T,5ARA W,5ARE J,5ARE K,5ARE L,5ARE M,5ARE N,5ARE O,5ARE P,5ARE Q,5ARE T,5ARE W,5ARH J,5ARH K,5ARH L,5ARH M,5ARH N,5ARH O,5ARH P,5ARH Q,5ARH T,5ARH W,5ARI J,5ARI K,5ARI L,5ARI M,5ARI N,5ARI O,5ARI P,5ARI Q,5ARI T,5ARI W,2ASG A,2ASG B,2ASG C,2ASG D,2ASG E,3ASN A,3ASN B,3ASN C,3ASN D,3ASN G,3ASN I,3ASN J,3ASN K,3ASN L,3ASN M,3ASN N,3ASN O,3ASN P,3ASN Q,3ASN T,3ASN V,3ASN W,3ASN X,3ASN Y,3ASN Z,3ASO A,3ASO B,3ASO C,3ASO D,3ASO G,3ASO I,3ASO J,3ASO K,3ASO L,3ASO M,3ASO N,3ASO O,3ASO P,3ASO Q,3ASO T,3ASO V,3ASO W,3ASO X,3ASO Y,3ASO Z,1AT9 A,1AT9 B,1AT9 C,1ATY A,2AT9 A,2AT9 B,2AT9 C,2ATK C,2ATK D,2ATK E,2ATK F,4ATV A,4ATV B,2AUI A,2AUL A,4AU5 A,4AU5 B,2AVL A,4AV3 A,4AV3 B,4AV6 A,4AV6 B,5AVQ A,5AVQ B,5AVQ G,5AVR A,5AVR B,5AVR G,5AVS A,5AVS B,5AVS G,5AVT A,5AVT B,5AVT G,5AVU A,5AVU B,5AVU G,5AVV A,5AVV B,5AVV G,5AVW A,5AVW B,5AVW G,5AVX A,5AVX B,5AVX G,5AVY A,5AVY B,5AVY G,5AVZ A,5AVZ B,5AVZ G,4AW6 B,5AW0 A,5AW0 B,5AW0 G,5AW1 A,5AW1 B,5AW1 G,5AW2 A,5AW2 B,5AW2 G,5AW3 A,5AW3 B,5AW3 G,5AW4 A,5AW4 B,5AW4 G,5AW5 A,5AW5 B,5AW5 G,5AW6 A,5AW6 B,5AW6 G,5AW7 A,5AW7 B,5AW7 G,5AW8 A,5AW8 B,5AW8 G,5AW9 A,5AW9 B,5AW9 G,5AWW Y,5AWW E,5AWW G,5AWZ A,2AXT A,2AXT B,2AXT C,2AXT D,2AXT E,2AXT F,2AXT H,2AXT I,2AXT J,2AXT K,2AXT L,2AXT M,2AXT T,2AXT X,2AXT Z,2AXT A,2AXT B,2AXT C,2AXT D,2AXT E,2AXT F,2AXT H,2AXT I,2AXT J,2AXT K,2AXT L,2AXT M,2AXT T,2AXT X,2AXT Z,5AX0 A,5AX1 A,3AYF A,3AYG A,3AYM A,3AYN A,4AYT A,4AYT B,4AYW A,4AYW B,4AYX A,4AYX B,5AYM A,5AYN A,5AYO A,4AZL A,5AZB A,5AZC A,2B0X A,2B2F A,2B2F B,2B2F C,2B2H A,2B2H B,2B2H C,2B2I A,2B2I B,2B2I C,2B2J A,2B2J B,2B2J C,3B29 A,3B29 B,3B29 C,4B2Q J,4B2Q K,4B2Q L,4B2Q M,4B2Q N,4B2Q O,4B2Q P,4B2Q Q,4B2Q R,4B2Q S,3B44 A,3B45 A,3B4R A,4B4A A,2B5F A,2B5F B,2B5F C,2B5F D,3B5D A,3B5D B,3B5W A,3B5W B,3B5X A,3B5X B,3B5Y A,3B5Y B,3B5Z A,3B5Z B,2B6O A,2B6O I,2B6O J,2B6O K,2B6P A,2B6P B,2B6P C,2B6P D,2B6Q A,2B6R A,2B6S A,2B6U A,2B6V A,3B60 A,3B60 B,3B61 A,4B61 A,2B76 C,2B76 D,2B76 O,2B76 P,4B7O A,3B8C A,3B8E C,3B8E D,3B8E H,1B9U A,3B9B A,3B9R A,3B9W A,3B9W B,3B9W C,3B9Y A,3B9Y B,3B9Y C,3B9Z A,3B9Z B,3B9Z C,1BA4 A,1BAC A,1BAD A,3BA6 A,2BBJ A,2BBJ B,2BBJ D,2BBJ E,2BBJ F,4BBJ A,1BCC C,1BCC D,1BCC E,1BCC G,1BCC J,1BCC M,1BCC N,1BCC P,1BCC R,1BCC U,1BCT A,2BCC C,2BCC D,2BCC E,2BCC G,2BCC J,3BCC C,3BCC D,3BCC E,3BCC G,3BCC J,3BCC M,3BCC N,3BCC P,3BCC R,3BCC U,1BE3 C,1BE3 D,1BE3 E,1BE3 G,1BE3 J,1BE3 K,3BEH A,3BEH B,3BEH C,3BEH D,4BEM A,4BEM B,4BEM C,4BEM D,4BEM E,4BEM F,4BEM G,4BEM H,4BEM I,4BEM J,4BEV A,4BEW A,4BEY A,4BEZ A,4BEZ B,1BGY C,1BGY D,1BGY E,1BGY G,1BGY J,1BGY K,1BGY O,1BGY P,1BGY Q,1BGY S,1BGY V,1BGY W,2BG9 A,2BG9 B,2BG9 C,2BG9 D,2BG9 E,4BGN A,4BGN C,4BGN D,4BGN E,1BH3 A,1BH3 B,1BH3 C,1BHA A,1BHB A,2BHW A,2BHW B,2BHW C,3BHS A,3BHS B,3BHS C,2BK3

A,1BL1 A,1BL8 A,1BL8 B,1BL8 C,1BL8 D,2BL2 A,2BL2 B,2BL2 C,2BL2 D,2BL2 E,2BL2 F,2BL2 G,2BL2 H,2BL2 I,2BL2 J,1BM1 A,1BM1 B,1BM1 C,2BMN A,1BNX A,2BNP A,2BNP B,2BNP C,2BNS A,2BNS B,2BNS C,1BOJ A,1BOK A,2BOB C,2BOB D,2BOB E,2BOB F,2BOC C,2BOC D,2BOC E,2BOC F,2BOZ H,2BOZ L,2BOZ M,4BOG A,4BOG B,4BOG C,4BOG D,4BOG E,4BOI A,4BOI B,4BOI C,4BOI D,4BOI E,4BON A,4BON B,4BON C,4BON D,4BON E,4BOO A,4BOO B,4BOO C,4BOO D,4BOO E,4BOR A,4BOR D,4BOR B,4BOR C,4BOR E,4BOT A,4BOT D,4BOT B,4BOT C,4BOT E,4BPD A,4BPD B,4BPD C,4BPM A,4BPM B,4BPM C,4BPQ A,4BPQ B,5BPS A,5BPS B,5BPS C,5BPS D,5BPS E,5BPS F,5BPS G,5BPS H,5BPS I,5BPS J,5BQ6 A,5BQ6 B,5BQ6 C,5BQ6 D,5BQ6 E,5BQ6 F,5BQ6 G,5BQ6 H,5BQ6 I,5BQ6 J,5BQA A,5BQA B,5BQA C,5BQA D,5BQA E,5BQA F,5BQA G,5BQA H,5BQA I,5BQA J,5BQJ A,5BQJ B,5BQJ C,5BQJ D,5BQJ E,5BQJ F,5BQJ G,5BQJ H,5BQJ I,5BQJ J,1BRD A,1BRD B,1BRD C,1BRR A,1BRR B,1BRR C,1BRX A,1BRX B,1BRX C,2BRD A,2BRD B,2BRD C,3BRY A,3BRZ A,4BRB A,4BRB B,4BRB C,4BRR A,4BRR B,4BRR C,2BS2 C,2BS2 F,2BS3 C,2BS3 F,2BS4 C,2BS4 F,3BS0 A,1BT9 A,1BT9 B,1BT9 C,1BTR A,1BTT A,4BUM X,4BUO A,5BUT I,5BUT J,3BVD A,3BVD B,3BVD C,4BV0 A,4BVN A,4BW5 A,4BW5 B,4BWB A,4BWZ A,5BW8 C,5BW8 D,1BXW A,1BY3 A,1BY5 A,2BY4 A,4BYG A,1BZK A,5BZ2 A,5BZ2 B,5BZ3 A,5BZ3 B,1C0V A,3C02 A,3C02 B,3C02 C,3C02 D,4C00 A,1C17 A,1C17 B,1C17 C,1C17 D,1C17 E,1C17 F,1C17 G,1C17 H,1C17 I,1C17 J,1C17 K,1C17 L,1C17 M,3C1G A,3C1G B,3C1G C,3C1H A,3C1H B,3C1H C,3C1I A,3C1I B,3C1I C,3C1J A,3C1J B,3C1J C,5C1M A,5C2T C,5C2T D,1C3W A,1C3W B,1C3W C,2C32 A,2C32 B,2C32 C,2C32 D,2C3E A,5C3L A,5C3L B,5C3L C,4C48 A,4C48 C,4C48 D,4C48 F,4C48 G,4C48 I,4C4V A,4C4V B,1C51 A,1C51 B,1C51 F,1C51 K,1C51 L,1C51 G,1C51 H,1C51 N,1C51 P,1C51 Q,1C51 R,1C51 S,1C51 W,1C51 Y,1C51 X,5C5X A,5C5X B,5C5X C,5C5X D,2C64 A,2C65 A,2C66 A,2C67 A,4C69 X,5C65 A,5C65 B,5C6N A,5C6O A,5C6P A,2C70 A,2C72 A,2C73 A,2C75 A,2C76 A,4C7R A,4C7R B,4C7R C,5C73 C,5C73 K,5C76 A,5C76 D,5C78 A,5C78 D,1C8R A,1C8R B,1C8R C,1C8S A,1C8S B,1C8S C,2C88 A,2C8K A,2C8L A,5C8J I,1C99 A,2C9M A,3C9L A,3C9M A,4C9G A,4C9H A,4C9J A,4C9Q A,4C9Q B,3CAP A,3CAP B,4CAD C,4CAS B,4CAS C,4CAS D,4CBC A,4CBC B,4CBC G,4CBC H,4CBJ A,4CBJ B,4CBJ C,4CBJ D,4CBJ E,4CBJ F,4CBJ G,4CBJ H,4CBJ I,4CBJ J,4CBJ K,4CBJ L,4CBJ M,4CBK A,4CBK B,4CBK C,4CBK D,4CBK E,4CBK F,4CBK G,4CBK H,4CBK I,4CBK J,4CBK K,4CBK L,4CBK M,4CDI A,4CDI C,4CDI B,4CDI D,4CDI E,4CDI F,2CFP A,2CFQ A,4CFG A,5CFB A,5CFB B,5CFB C,5CFB D,5CFB E,4CG5 A,4CG5 B,4CG5 C,4CG6 A,4CG6 B,4CG6 C,4CG6 D,4CG7 A,4CG7 B,4CG7 C,5CGC A,5CGD A,3CHX A,3CHX B,3CHX C,3CHX M,3CHX E,3CHX F,3CHX G,3CHX N,3CHX I,3CHX J,3CHX K,3CHX O,4CHV A,4CHV B,4CHV C,4CHV D,4CHW A,4CHW B,4CHW C,4CHW D,5CH4 Y,5CH4 E,5CH4 G,3CIR C,3CIR D,3CIR O,3CIR P,4CJZ A,4CJZ B,4CJZ C,4CK0 A,4CK0 B,4CK0 C,1CLT L,1CLT M,3CLL A,3CLL B,3CLL C,3CLL D,3CN5 A,3CN5 B,3CN5 C,3CN5 D,3CN6 A,3CN6 C,3CN6 D,3CN6 E,3COC A,3COC D,3COC C,3COD A,3COD D,3COD C,4COF A,4COF B,4COF C,4COF D,4COF E,2CPB A,2CPS A,4CRT A,3CSL A,3CSN A,4CSK A,4CSK B,4CSK C,4CSK D,4CTD A,5CTG A,5CTG B,5CTG C,5CTH A,5CTH B,5CTH C,4CU4 A,1CWQ A,1CWQ C,1CWQ E,3CWB C,3CWB D,3CWB E,3CWB G,3CWB J,3CWB P,3CWB Q,3CWB R,3CWB T,3CWB W,3CX5 C,3CX5 D,3CX5 E,3CX5 H,3CX5 I,3CX5 N,3CX5 O,3CX5

P,3CX5 S,3CX5 T,3CXH C,3CXH D,3CXH E,3CXH H,3CXH I,3CXH N,3CXH O,3CXH P,3CXH S,3CXH T,2CYD A,2CYD B,2CYD C,2CYD D,2CYD E,2CYD F,2CYD G,2CYD H,2CYD I,2CYD J,4CY4 A,4CY4 B,4CY4 C,4CY4 D,4CY4 E,4CZ8 A,4CZ8 B,4CZ9 A,4CZ9 B,4CZA A,4CZA B,4CZB A,4CZB B,4D0A B,4D0A A,4D1A A,4D1B A,4D1C A,4D1D A,2D2C A,2D2C B,2D2C C,2D2C D,2D2C E,2D2C F,2D2C G,2D2C H,2D2C N,2D2C O,2D2C P,2D2C Q,2D2C R,2D2C S,2D2C T,2D2C U,4D2B A,4D2C A,4D2D A,4D2E A,4D2E B,4D2E C,3D31 C,3D31 D,3D38 H,3D38 L,3D38 M,3D4S A,2D57 A,2D57 B,2D57 C,2D57 D,4D51 A,4D5B A,4D5D A,4D5U A,4D5U B,4D5U C,5D56 A,5D56 B,5D56 C,5D57 A,5D57 B,5D57 C,5D58 A,5D59 A,5D5A A,5D5B A,5D5D A,4D6T C,4D6T D,4D6T E,4D6T G,4D6T J,4D6T P,4D6T Q,4D6T R,4D6T T,4D6T W,4D6U C,4D6U D,4D6U E,4D6U G,4D6U J,4D6U P,4D6U Q,4D6U R,4D6U T,4D6U W,3D9B A,3D9B B,3D9B C,3D9S A,3D9S B,3D9S C,3D9S D,5D91 A,5D92 D,5D92 A,4DAJ A,5DA0 A,2DB4 A,2DB4 B,2DB4 C,2DB4 D,2DB4 E,2DB4 F,2DB4 G,2DB4 H,2DB4 I,2DB4 J,4DBL A,4DBL B,4DCB A,1DDD 1,1DDD 2,1DDD 3,1DDD 4,1DDD 5,1DDD 6,1DDD 7,3DDL A,3DDR B,3DET A,3DET B,2DF9 A,1DHL A,2DHH A,2DHH B,2DHH C,2DHL A,3DH4 A,3DH4 B,3DHL A,3DHW A,3DHW B,5DHG A,5DHH A,3DIN C,3DIN D,3DIN E,4DJH A,4DJH B,4DJI A,4DJK A,5DJQ A,5DJQ B,5DJQ C,5DJQ N,4DKL A,4DKL B,3DL8 G,3DL8 C,3DL8 E,5DL5 A,5DL6 A,5DL7 A,5DL8 B,4DNR A,4DNR F,4DNR I,4DNT A,4DNT F,4DNT I,5DN6 J,5DN6 K,5DN6 L,5DN6 M,5DN6 N,5DN6 O,5DN6 P,5DN6 Q,5DN6 R,5DN6 S,5DN6 T,5DN6 U,5DN6 X,5DN6 Y,1DOP A,1DOP D,4DOJ A,4DOJ B,4DOJ C,4DOP A,4DOP F,4DOP I,2DQS A,3DQB A,3DQB C,2DR6 A,2DR6 B,2DR6 C,2DRD A,2DRD B,2DRD C,1DS8 L,1DS8 M,1DS8 H,3DSY L,3DSY M,3DSY H,3DTA L,3DTA M,3DTA H,3DTR L,3DTR M,3DTR H,3DTS L,3DTS M,3DTS H,3DTU A,3DTU B,3DU2 L,3DU2 M,3DU2 H,3DU3 L,3DU3 M,3DU3 H,3DUQ L,3DUQ M,3DUQ H,5DUO A,5DUO B,1DV3 L,1DV3 M,1DV3 H,1DV6 L,1DV6 M,1DV6 H,4DVE A,2DW3 A,2DWD C,2DWD D,2DWD E,2DWD F,2DWE C,2DWE D,2DWE E,2DWE F,3DWN A,3DWO X,3DWW A,3DWW B,3DWW C,4DW0 A,4DW0 B,4DW0 C,4DW1 A,4DW1 B,4DW1 C,1DX7 A,1DXR H,1DXR L,1DXR M,1DXZ A,4DX5 A,4DX5 B,4DX5 C,4DX6 A,4DX6 B,4DX6 C,4DX7 A,4DX7 B,4DX7 C,4DXW A,4DXW B,4DXW C,4DXW D,2DYR A,2DYR B,2DYR C,2DYR D,2DYR G,2DYR I,2DYR J,2DYR K,2DYR L,2DYR M,2DYR N,2DYR O,2DYR P,2DYR Q,2DYR T,2DYR V,2DYR W,2DYR X,2DYR Y,2DYR Z,2DYS A,2DYS B,2DYS C,2DYS D,2DYS G,2DYS I,2DYS J,2DYS K,2DYS L,2DYS M,2DYS N,2DYS O,2DYS P,2DYS Q,2DYS T,2DYS V,2DYS W,2DYS X,2DYS Y,2DYS Z,5DYE A,5DYE B,5DYE C,5DYE D,1DZE A,1DZE B,1DZE C,3DZM A,1E0P A,1E0P B,1E0P C,1E12 A,1E12 C,1E12 D,1E14 L,1E14 M,1E14 H,4E1S A,4E1T A,5E1J A,5E1J B,1E54 A,1E6D L,1E6D M,1E6D H,1E7P C,1E7P F,2E74 A,2E74 B,2E74 C,2E74 D,2E74 E,2E74 F,2E74 G,2E74 H,2E74 I,2E74 J,2E74 K,2E74 L,2E74 M,2E74 N,2E74 P,2E74 Q,2E75 A,2E75 B,2E75 C,2E75 D,2E75 E,2E75 F,2E75 G,2E75 H,2E75 I,2E75 J,2E75 K,2E75 L,2E75 M,2E75 N,2E75 P,2E75 Q,2E76 A,2E76 B,2E76 C,2E76 D,2E76 E,2E76 F,2E76 G,2E76 H,2E76 I,2E76 J,2E76 K,2E76 L,2E76 M,2E76 N,2E76 P,2E76 Q,3E83 A,3E83 C,3E83 D,3E83 E,3E86 A,3E86 C,3E86 D,3E86 E,3E89 A,3E89 C,3E89 D,3E89 E,3E8B A,3E8B C,3E8B D,3E8B E,3E8F A,3E8F C,3E8F D,3E8F E,3E8G A,3E8G C,3E8G D,3E8G E,3E8H A,3E8H C,3E8H D,3E8H E,3E9J C,2EAR A,2EAT A,2EAU A,3EAM A,3EAM B,3EAM C,3EAM D,3EAM E,4EA3 A,4EEB A,4EEB B,4EEB C,4EEB D,4EEB E,4EED A,4EED B,4EED

C,4EED D,4EED E,3EFF K,3EFF L,3EFF M,3EFF N,3EFM A,3EGW C,3EGW F,1EHK A,1EHK B,1EHK C,3EH3 A,3EH3 B,3EH3 C,3EH4 A,3EH4 B,3EH4 C,3EH5 A,3EH5 B,3EH5 C,3EHB A,3EHB B,3EHZ A,3EHZ B,3EHZ C,3EHZ D,3EHZ E,5EH4 A,5EH4 B,5EH6 A,2EI4 A,2EI4 B,2EI4 C,2EIJ A,2EIJ B,2EIJ C,2EIJ D,2EIJ G,2EIJ I,2EIJ J,2EIJ K,2EIJ L,2EIJ M,2EIJ N,2EIJ O,2EIJ P,2EIJ Q,2EIJ T,2EIJ V,2EIJ W,2EIJ X,2EIJ Y,2EIJ Z,2EIK A,2EIK B,2EIK C,2EIK D,2EIK G,2EIK I,2EIK J,2EIK K,2EIK L,2EIK M,2EIK N,2EIK O,2EIK P,2EIK Q,2EIK T,2EIK V,2EIK W,2EIK X,2EIK Y,2EIK Z,2EIL A,2EIL B,2EIL C,2EIL D,2EIL G,2EIL I,2EIL J,2EIL K,2EIL L,2EIL M,2EIL N,2EIL O,2EIL P,2EIL Q,2EIL T,2EIL V,2EIL W,2EIL X,2EIL Y,2EIL Z,2EIM A,2EIM B,2EIM C,2EIM D,2EIM G,2EIM I,2EIM J,2EIM K,2EIM L,2EIM M,2EIM N,2EIM O,2EIM P,2EIM Q,2EIM T,2EIM V,2EIM W,2EIM X,2EIM Y,2EIM Z,2EIN A,2EIN B,2EIN C,2EIN D,2EIN G,2EIN I,2EIN J,2EIN K,2EIN L,2EIN M,2EIN N,2EIN O,2EIN P,2EIN Q,2EIN T,2EIN V,2EIN W,2EIN X,2EIN Y,2EIN Z,3EI0 A,3EI0 B,3EI0 C,3EI0 D,3EI0 E,4EIY A,3EJY A,3EJY B,3EJZ A,3EJZ B,4EJ4 A,1EK9 A,1EK9 B,1EK9 C,4EKW A,4EKW B,4EKW E,4EKW F,5EK0 A,5EK0 B,5EK0 C,5EK0 D,5EKE A,5EKE B,5EKE C,5EKE D,5EKP C,5EKP A,5EKP B,5EKP D,5EKQ A,3EML A,3EMN X,3EMO C,3EMO A,3EMO B,4ENE A,4ENE B,4EPA A,1EQ8 A,2ERV A,2ERV B,5ER7 B,5ER7 A,5ER7 C,5ER7 D,5ER7 E,5ER7 F,5ERA B,5ERA A,5ERA C,5ERA D,5ERA E,5ERA F,1ET2 S,1ET3 S,2EVU A,2EVU B,2EVU C,2EVU D,4EV6 A,4EV6 B,4EV6 C,4EV6 D,4EV6 E,2EXW A,2EXW B,2EXY A,2EXY B,1EYS L,1EYS M,1EYS H,1EZV C,1EZV D,1EZV E,1EZV G,1EZV I,1EZV L,1EZV M,1EZV N,1EZV R,1EZV S,2EZ0 A,2EZ0 B,4EZC A,4EZC B,4EZC C,4EZD A,4EZD B,4EZD C,2F1C X,2F1T A,2F1U A,2F1U B,2F1U C,2F1U D,2F1U E,2F1U F,2F1U G,2F1V A,2F1V B,2F2B A,2F2B B,2F2B C,2F2B D,3F3A A,3F3A B,3F3C A,3F3C B,3F3D A,3F3D B,3F3E A,3F3E B,4F35 B,4F35 A,1F4Z A,1F4Z B,1F4Z C,3F48 A,3F48 B,3F4I A,3F4I B,3F4J A,3F4J B,4F4C A,4F4L A,4F4L B,4F4L C,4F4L D,4F4S K,4F4S L,4F4S M,4F4S N,4F4S O,4F4S P,4F4S Q,4F4S R,4F4S S,4F4S T,1F50 A,1F50 B,1F50 C,3F5W C,3F5W D,3F5W E,3F5W F,1F6G A,1F6G B,1F6G C,1F6G D,1F6N L,1F6N M,1F6N H,2F75 A,3F7V C,3F7V D,3F7V E,3F7V F,3F7Y C,3F7Y D,3F7Y E,3F7Y F,1F88 A,1F88 C,4F8H A,4F8H B,4F8H C,4F8H D,4F8H E,5F8U B,5F8U A,2F93 A,2F93 B,2F93 C,2F93 D,2F95 A,2F95 B,2F95 C,2F95 D,4FA7 A,4FA7 B,4FA7 C,4FAA A,4FAA B,4FAA C,1FBB A,1FBB B,1FBB C,1FBK A,1FBK B,1FBK C,2FBW C,2FBW D,3FB5 C,3FB5 D,3FB5 E,3FB5 F,3FB6 C,3FB6 D,3FB6 E,3FB6 F,3FB7 C,3FB7 D,3FB7 E,3FB7 F,3FB8 C,3FB8 E,3FB8 F,3FB8 G,4FBY A,4FBY B,4FBY C,4FBY D,4FBY E,4FBY F,4FBY H,4FBY I,4FBY J,4FBY K,4FBY L,4FBY M,4FBY T,4FBY Y,4FBY X,4FBY Y,4FBY Z,4FBY G,4FBY N,4FBY P,4FBY Q,4FBY R,4FBY S,4FBY W,4FBY A,4FBY B,4FBY C,4FBY D,4FBY E,4FBY G,4FBY M,4FBY J,4FBY K,4FBY L,4FBZ A,4FBZ B,4FBZ C,1FCP A,2FCP A,4FC4 A,4FC4 B,4FC4 C,4FC4 D,4FC4 E,1FDF A,1FDM A,1FE1 A,1FE1 B,1FE1 C,1FE1 D,1FE1 E,1FE1 F,1FE1 G,1FE1 J,1FE1 K,1FE1 L,1FE1 M,1FE1 N,1FE1 O,1FE1 P,1FEP A,2FEC A,2FEC B,2FED A,2FED B,2FEE A,2FEE B,4FE1 A,4FE1 B,4FE1 F,4FE1 I,4FE1 J,4FE1 K,4FE1 L,4FE1 M,4FE1 X,1FFT A,1FFT B,1FFT C,1FFT D,2FF9 A,2FGQ X,2FGQ A,2FGQ B,2FGR A,2FGR C,2FGR E,3FGO A,4FG6 A,4FG6 B,5FGZ A,3FH6 F,3FH6 G,3FHH A,1FI1 A,3FI1 A,3FI1 B,3FID A,3FIP A,4FI3 A,4FI3 B,5FIJ J,5FIJ K,5FIJ L,5FIJ M,5FIJ N,5FIJ O,5FIJ P,5FIJ Q,5FIJ T,5FIJ W,5FIK J,5FIK K,5FIK L,5FIK M,5FIK N,5FIK O,5FIK P,5FIK Q,5FIK T,5FIK W,5FIL J,5FIL K,5FIL L,5FIL M,5FIL N,5FIL

O,5FIL P,5FIL Q,5FIL T,5FIL W,1FJK A,1FJP A,2FKW A,2FKW B,2FKW C,2FKW D,2FKW E,2FKW F,2FKW G,2FKW H,2FKW I,2FKW J,2FKW K,2FKW L,2FKW M,2FKW N,2FKW O,2FKW P,2FKW R,2FKW S,4FMS B,1FNP L,1FNP M,1FNP H,1FNQ L,1FNQ M,1FNQ H,5FN2 B,5FN2 D,5FN2 A,5FN2 C,5FN5 B,5FN5 D,5FN5 A,5FN5 C,4FOZ A,3FPB A,3FPS A,4FPD A,4FPD B,4FPD C,1FQU A,1FQY A,1FQY B,1FQY C,1FQY D,4FQE A,4FRT A,4FRX A,4FSO A,4FSP A,4FT6 A,4FTP A,4FTP B,4FUV A,4FUV B,1FW2 A,1FW3 A,1FW3 B,3FWL A,1FX8 A,1FX8 B,1FX8 C,1FX8 D,4FXZ A,4FXZ B,2FYN A,2FYN B,2FYN C,2FYN D,2FYN E,2FYN F,2FYU C,2FYU D,2FYU E,2FYU G,2FYU J,2FYU K,3FYE A,3FYE B,3FYI A,3FYI B,3FYX A,3FYX B,3FYX C,4FY0 A,4FY0 B,4FZ0 A,4FZ0 B,4FZ0 C,4FZ1 A,4FZ1 B,4FZ1 E,2G1X A,4G1U A,4G1U B,2G2A A,3G5U A,3G60 A,3G61 A,3G6B A,3G6B B,3G7F H,3G7F L,3G7F M,4G70 A,4G70 B,4G70 C,4G71 A,4G71 B,4G71 C,4G72 A,4G72 B,4G72 C,4G7Q A,4G7Q B,4G7Q C,4G7R A,4G7R B,4G7R C,4G7S A,4G7S B,4G7S C,4G7V S,4G7Y S,2G87 A,4G80 S,4G80 T,1G90 A,3GB7 C,3GB7 D,3GB7 E,3GB7 F,4GBR A,4GBY A,4GBZ A,4GC0 A,4GCP A,4GCQ A,4GCS A,3GD8 A,3GD8 B,3GD8 C,3GD8 D,4GD3 S,4GD3 T,4GD3 Q,4GD3 R,4GD3 A,4GD3 B,2GE4 A,4GEY A,1GFM A,1GFM B,1GFM C,1GFN A,1GFN B,1GFN C,1GFO A,1GFO B,1GFO C,1GFP A,1GFP B,1GFP C,1GFQ A,1GFQ B,1GFQ C,2GFP A,2GFZ A,4GF4 A,2GIF A,2GIF B,2GIF C,3GI8 C,3GI9 C,3GIA A,3GJC A,3GJC B,3GJD A,3GJD B,2GMR L,2GMR M,2GMR H,2GNU H,2GNU L,2GNU M,3GP6 A,4GP4 A,4GP4 B,4GP4 C,4GP5 A,4GP5 B,4GP5 C,4GP8 A,4GP8 B,4GP8 C,4GPO A,4GPO B,2GR7 A,2GR7 B,2GR7 C,2GR7 D,2GR7 E,2GR7 F,2GR8 A,2GR8 C,2GR8 D,2GRX A,4GRV A,2GSK A,2GSM A,2GSM B,1GU8 A,1GUE A,2GUF A,3GWU A,3GWU B,3GWV A,3GWV B,3GWW A,3GWW B,4GX0 A,4GX0 B,4GX0 E,4GX0 F,4GX1 A,4GX1 B,4GX1 E,4GX1 F,4GX2 A,4GX2 B,4GX2 E,4GX2 F,4GX5 A,4GX5 B,4GX5 E,4GX5 F,4GYC A,4GYC B,4GYC C,4GYC D,1GZM A,1GZM C,4H0L A,4H0L B,4H0L C,4H0L D,4H0L E,4H0L F,4H0L G,4H0L H,4H0L I,4H0L J,4H0L K,4H0L L,4H0L M,4H0L N,4H0L O,4H0L P,3H1H C,3H1H D,3H1H E,3H1H G,3H1H J,3H1H P,3H1H Q,3H1H R,3H1H T,3H1H W,3H1I C,3H1I D,3H1I E,3H1I G,3H1I J,3H1I P,3H1I Q,3H1I R,3H1I T,3H1I W,3H1J C,3H1J D,3H1J E,3H1J G,3H1J J,3H1J P,3H1J Q,3H1J R,3H1J T,3H1J W,3H1K C,3H1K D,3H1K E,3H1K G,3H1K J,3H1K P,3H1K Q,3H1K R,3H1K T,3H1K W,3H1L C,3H1L D,3H1L E,3H1L G,3H1L J,3H1L P,3H1L Q,3H1L R,3H1L T,3H1L W,4H13 A,4H13 B,4H13 C,4H13 D,4H13 E,4H13 F,4H13 G,4H13 H,4H13 I,4H13 J,4H13 K,4H13 L,4H13 M,4H13 N,4H13 O,4H13 P,4H1D A,4H1D B,4H1D C,4H1W A,4H1W B,1H2S A,1H2S B,1H2S C,1H2S D,2H2P A,2H2P B,2H2S A,2H2S B,2H3O A,4H33 A,4H33 B,4H33 C,4H33 D,4H37 A,4H37 B,4H37 C,4H37 D,4H44 A,4H44 B,4H44 C,4H44 D,4H44 E,4H44 F,4H44 G,4H44 H,4H44 I,4H44 J,4H44 K,4H44 L,4H44 M,4H44 N,4H44 O,4H44 P,1H68 A,1H6I A,1H6S 1,2H88 C,2H88 D,2H89 C,2H89 D,2H8A A,2H8A B,2H8A C,2H8P C,2H8P D,2H8P E,2H8P F,2H8P G,2H8P H,2H8P I,2H8P J,3H90 A,3H9V A,3H9V B,3H9V C,4H99 L,4H99 M,4H99 H,4H9L L,4H9L M,4H9L H,2HAC A,2HAC B,3HAN A,3HAO A,3HAO D,3HAO C,3HAP A,3HAQ A,3HAR A,3HAS A,3HB3 A,3HB3 B,4HBH L,4HBH M,4HBH H,4HBJ L,4HBJ M,4HBJ H,2HDF A,2HDI A,3HD6 A,3HD6 B,3HD6 C,3HD7 A,3HD7 B,2HE6 ,4HE8 B,4HE8 D,4HE8 E,4HE8 F,4HE8 G,4HE8 I,4HE8 C,4HEA A,4HEA J,4HEA K,4HEA L,4HEA M,4HEA N,4HEA H,2HFE C,2HFE D,2HFE E,2HFE F,2HFE G,2HFE H,2HFE I,2HFE J,3HFX A,3HFX B,3HFX C,4HFB A,4HFB B,4HFB C,4HFB D,4HFB E,4HFC

A,4HFC B,4HFC C,4HFC D,4HFC E,4HFD A,4HFD B,4HFD C,4HFD D,4HFD E,4HFE A,4HFE B,4HFE C,4HFE D,4HFE E,4HFH A,4HFH B,4HFH C,4HFH D,4HFH E,4HFI A,4HFI B,4HFI C,4HFI D,4HFI E,2HG3 L,2HG3 M,2HG3 H,2HG5 C,2HG5 D,2HG5 E,2HG5 F,2HG5 G,2HG5 H,2HG5 I,2HG5 J,2HG9 L,2HG9 M,2HG9 H,4HG6 A,4HG6 B,2HH1 L,2HH1 M,2HH1 H,2HHK L,2HHK M,2HHK H,2HI7 B,2HIT L,2HIT M,2HIT H,2HJ6 L,2HJ6 M,2HJ6 H,2HJF C,2HJF D,2HJF E,2HJF F,3HKK A,3HKK B,3HKK C,4HKR A,4HKR B,4HKR C,4HKR D,4HKR E,4HKR F,4HKS A,4HKS B,4HKS C,4HKS D,4HKS E,4HKS F,2HLF A,2HLF B,4HMK A,4HMK B,2HN2 A,2HN2 B,2HN2 C,2HN2 D,2HN2 E,4HOD A,4HOD B,2HPY A,3HPL C,3HPL D,3HPL E,3HPL F,2HQC A,2HQC B,2HQC C,2HQD A,2HQD B,2HQD C,2HQF A,2HQF B,2HQF C,2HQG A,2HQG B,2HQG C,4HQJ C,4HQJ D,4HQJ E,2HRT A,2HRT B,2HRT C,2HT2 A,2HT2 B,2HT3 A,2HT3 B,2HT4 A,2HT4 B,2HTG A,2HTK A,2HTK B,2HTL A,2HTL B,4HTS A,4HTT A,4HUK A,4HUL A,4HUM A,4HUN A,4HUQ S,4HUQ T,2HVJ C,2HVJ D,2HVJ E,2HVJ F,2HVK C,2HVK D,2HVK E,2HVK F,3HW9 A,3HWB A,3HWB C,3HWB D,4HW9 A,4HW9 B,4HW9 C,4HW9 D,4HW9 E,4HW9 F,4HW9 G,4HWA A,4HWA B,4HWA C,4HWA D,4HWA E,4HWA F,4HWA G,4HWL A,1HXT A,1HXT B,1HXT C,1HXU A,1HXU B,1HXU C,1HXX A,1HXX B,1HXX C,2HYD A,2HYD B,2HYN A,2HYN B,2HYN C,2HYN D,2HYN E,4HYC A,4HYC B,4HYC C,4HYC D,4HYD A,4HYD B,4HYD C,4HYD D,4HYG A,4HYJ A,4HYO A,4HYO B,4HYO C,4HYO D,4HYT A,4HYT B,4HYT G,4HYX A,1HZX A,1HZX C,4HZ3 D,4HZ3 C,4HZ3 A,4HZ3 B,4HZU T,4HZU S,4I0U A,4I0U B,4I0U C,4I0U D,4I0U E,1I15 A,1I15 B,1I15 C,1I15 D,1I15 E,1I15 F,1I15 G,2I1X A,2I1X B,2I1X C,2I20 A,2I20 B,2I20 C,2I21 A,2I21 B,2I21 C,2I35 A,2I36 A,2I36 B,2I37 A,2I37 B,3I4D L,3I4D M,3I4D H,2I5N H,2I5N L,2I5N M,3I5D A,3I5D B,3I5D C,2I6W A,2I6W B,2I6W C,1I78 A,4I7Z A,4I7Z B,4I7Z C,4I7Z D,4I7Z E,4I7Z F,4I7Z G,4I7Z H,4I7Z I,4I7Z J,4I7Z K,4I7Z L,4I7Z M,4I7Z N,4I7Z O,4I7Z P,4I9W A,4I9W B,2IAH A,4IA4 A,4IA4 B,4IA4 C,4IA4 D,4IAQ A,4IAR A,2IBZ C,2IBZ D,2IBZ E,2IBZ G,2IBZ I,4IB4 A,2IC8 A,3IFX A,3IFX B,3IFX C,3IFX D,4IFF A,4IFF B,1IGK A,1IGK B,1IGK C,1IGK D,1IGK E,1IGK F,1IGK G,3IGA C,3IGA D,3IGA E,3IGA F,1IH5 A,1IH5 B,1IH5 C,1IH5 D,2IH1 C,2IH1 D,2IH1 E,2IH1 F,2IH3 C,2IH3 D,2IH3 E,2IH3 F,1IIJ A,1IIV A,1IIV B,1IIV C,2IIL ,1IJD A,1IJD B,1IJD C,1IJD D,1IJD E,1IJD F,1IJD G,1IJD H,1IJD I,1IJD J,1IJD K,1IJD L,1IJD M,1IJD N,1IJD P,1IJD Q,1IJD R,1IJD S,1IJP A,3IJ4 A,3IJ4 B,3IJ4 C,2IK3 ,2IK5 ,4IKV A,4IKW A,4IKX A,4IKY A,4IKZ A,1ILD A,1ILX A,1ILX B,1ILX C,1ILX D,1ILX E,1ILX F,1ILX G,1ILX J,1ILX K,1ILX L,1ILX M,1ILX N,1ILX O,1ILX P,1ILZ A,4IL3 A,4IL4 A,4IL4 B,4IL4 C,4IL4 D,4IL4 E,4IL6 A,4IL6 B,4IL6 C,4IL6 D,4IL6 E,4IL6 F,4IL6 H,4IL6 I,4IL6 J,4IL6 K,4IL6 L,4IL6 M,4IL6 T,4IL6 Y,4IL6 X,4IL6 Z,4IL6 R,4IL6 A,4IL6 B,4IL6 C,4IL6 D,4IL6 E,4IL6 F,4IL6 H,4IL6 I,4IL6 J,4IL6 K,4IL6 L,4IL6 M,4IL6 T,4IL6 Y,4IL6 X,4IL6 Z,4IL9 A,4IL9 B,4IL9 C,4IL9 D,4IL9 E,4ILA A,4ILA B,4ILA C,4ILA D,4ILA E,4ILB A,4ILB B,4ILB C,4ILB D,4ILB E,4ILC A,4ILC B,4ILC C,4ILC D,4ILC E,1IM0 A,4IN5 L,4IN5 M,4IN5 H,4IN6 L,4IN6 M,4IN6 H,4IN7 H,4IN7 L,4IN7 M,3IPD A,3IPD B,2IQ4 ,2IQK A,2IQK C,2IQL A,2IQM A,2IQN A,2IQO A,2IQP A,2IQR A,2IQS A,2IQU A,2IQV A,2IQW A,2IRV A,2IRV D,2IRV C,3IR5 C,3IR5 F,3IR6 C,3IR6 F,3IR7 C,3IR7 H,4IRE A,4IRE B,4IRE C,4IRE D,4IRE E,2ITA A,2ITC C,2ITC D,2ITC E,2ITC F,2ITD C,2ITD D,2ITD E,2ITD F,2IUB A,2IUB B,2IUB C,2IUB D,2IUB E,4IU8 A,4IU9 B,1IW6 A,1IW6 B,1IW6 C,1IW9 A,1IW9 B,1IW9 C,1IWG A,1IWG B,1IWG C,1IWO A,2IWV A,2IWW A,1IXF A,1IXF B,1IXF C,3IXZ A,3IXZ B,4IXQ A,4IXQ B,4IXQ C,4IXQ D,4IXQ E,4IXQ F,4IXQ

H,4IXQ I,4IXQ J,4IXQ K,4IXQ L,4IXQ M,4IXQ T,4IXQ Y,4IXQ X,4IXQ Z,4IXQ Y,4IXQ A,4IXQ B,4IXQ C,4IXQ D,4IXQ E,4IXQ F,4IXQ H,4IXQ I,4IXQ J,4IXQ K,4IXQ L,4IXQ M,4IXQ T,4IXQ G,4IXQ X,4IXQ Z,4IXQ G,4IXR A,4IXR B,4IXR C,4IXR D,4IXR E,4IXR F,4IXR H,4IXR I,4IXR J,4IXR K,4IXR L,4IXR M,4IXR T,4IXR Y,4IXR X,4IXR Z,4IXR G,4IXR A,4IXR B,4IXR C,4IXR D,4IXR E,4IXR F,4IXR H,4IXR I,4IXR J,4IXR K,4IXR L,4IXR M,4IXR T,4IXR G,4IXR X,4IXR Z,4IXR Y,3IYZ A,3IYZ B,3IYZ C,3IYZ D,3IZ1 A,3IZ1 B,3IZ1 C,3IZ1 D,3IZ1 E,3IZ1 F,3IZ2 A,3IZ2 B,3IZ2 C,3IZ2 D,3IZ2 E,3IZ2 F,4IZM A,4IZM B,4IZM C,3J08 A,3J08 B,3J09 A,3J09 B,4J05 A,2J1H A,2J1N A,2J1N B,2J1N C,3J1Z P,3J1Z Q,4J2T A,1J4N A,1J4N B,1J4N C,1J4N D,2J4U P,2J4U Q,2J4U R,2J4Y A,3J41 A,3J41 B,3J41 C,3J41 D,4J4Q A,2J58 A,2J58 B,2J58 C,2J58 D,2J58 E,2J58 F,2J58 G,2J58 H,3J5P B,3J5P A,3J5P C,3J5P D,3J5Q D,3J5Q B,3J5Q E,3J5Q G,3J5R B,3J5R A,3J5R C,3J5R D,1J7F A,1J7F B,1J7F C,1J7F D,1J7F E,1J7F F,1J7F G,1J7F H,1J7F J,1J7F I,1J7F K,1J7F L,2J7A C,2J7A F,3J7T A,4J72 A,4J72 B,4J7C K,4J7C L,4J7T A,4J7T B,4J7T C,4J7Y A,4J7Y B,4J7Y C,2J8C H,2J8C L,2J8C M,2J8D H,2J8D L,2J8D M,2J8S A,2J8S B,2J8S C,3J8A F,3J8A G,1J95 A,1J95 B,1J95 C,1J95 D,3J9J A,3J9J B,3J9J C,3J9J D,3J9P D,3J9P A,3J9P B,3J9P C,3J9T Y,3J9T R,3J9T U,3J9T V,3J9T T,3J9T W,3J9T S,3J9T X,3J9T Z,3J9T A,3J9U U,3J9U X,3J9U A,3J9U R,3J9U Z,3J9U S,3J9U Y,3J9U T,3J9U V,3J9U W,3J9V R,3J9V U,3J9V X,3J9V Y,3J9V W,3J9V Z,3J9V V,3J9V A,3J9V S,3J9V T,4J9U A,4J9U B,2JAF A,2JAF B,2JAF C,2JAG A,2JAG B,2JAG C,3JAC A,3JAC B,3JAC C,3JAD A,3JAD B,3JAD C,3JAD D,3JAD E,3JAE A,3JAE B,3JAE C,3JAE D,3JAE E,3JAF A,3JAF B,3JAF C,3JAF D,3JAF E,3JAV A,3JAV B,3JAV C,3JAV D,4JA3 A,4JA4 A,1JB0 A,1JB0 B,1JB0 F,1JB0 I,1JB0 J,1JB0 K,1JB0 L,1JB0 M,1JB0 X,2JBL H,2JBL L,2JBL M,3JBR A,3JBR E,4JBW F,4JBW G,3JC2 1,3JC2 2,3JC2 3,4JC6 A,4JC6 B,4JC6 C,4JC6 D,4JC7 A,4JC7 B,4JC7 C,4JCZ A,4JCZ B,4JCZ C,1JDM A,1JFP A,4JFB A,4JFB B,4JFB C,1JGJ A,1JGW L,1JGW M,1JGW H,1JGX L,1JGX M,1JGX H,1JGY L,1JGY M,1JGY H,1JGZ L,1JGZ M,1JGZ H,1JH0 L,1JH0 M,1JH0 H,2JIY H,2JIY L,2JIY M,2JJ0 H,2JJ0 L,2JJ0 M,2JK4 A,2JK5 C,2JK5 D,2JK5 E,2JK5 F,4JKV A,4JKV B,2JLN A,2JMM A,1JO5 A,2JO1 A,2JP3 A,1JQ1 A,1JQ1 B,1JQ1 C,1JQ1 D,1JQ2 A,1JQ2 B,1JQ2 C,1JQ2 D,2JQY A,4JQ6 A,4JQ6 B,4JQ6 C,4JQ6 D,4JQ6 E,4JQ6 F,4JR8 A,4JR8 B,4JR8 C,4JR9 A,4JRE A,4JRZ A,4JRZ B,4JRZ C,2JTW A,3JTY A,4JTA B,4JTA D,4JTA F,4JTA H,4JTC B,4JTD B,1JV6 A,1JV7 A,1JVM A,1JVM B,1JVM C,1JVM D,2JWA A,2JWA B,3JYC A,3JYC B,3JYC C,3JYC D,2K0L A,3K03 A,3K03 C,3K03 D,3K03 E,3K04 A,3K04 C,3K04 D,3K04 E,3K06 A,3K06 C,3K06 D,3K06 E,3K07 A,3K07 B,3K07 C,3K08 A,3K08 C,3K08 D,3K08 E,3K0D A,3K0D C,3K0D D,3K0D E,3K0G A,3K0G C,3K0G D,3K0G E,3K0I A,3K0I B,3K0I C,4K0E A,4K0E B,4K0E C,4K0J A,4K0J B,4K0J C,2K1A A,2K1K A,2K1K B,2K1L A,2K1L B,3K19 A,3K19 B,3K19 C,3K1B A,3K1B B,3K1B C,4K1C A,1K24 A,2K21 A,2K3C A,3K3F A,3K3F B,3K3F C,3K3G A,3K3G B,3K3G C,4K3B A,4K3C A,1K4C C,1K4C F,1K4C I,1K4C L,1K4D C,1K4D F,1K4D I,1K4D L,2K4T A,2K58 B,2K59 B,4K5Y A,1K6L L,1K6L M,1K6L H,1K6N L,1K6N M,1K6N H,2K73 A,2K74 A,4K7Q A,4K7Q B,4K7Q C,4K7R A,4K7R B,4K7R C,1K9N A,1K9N B,1K9N C,1K9N D,1K9N E,2K9J A,2K9J B,2K9P A,2K9Y A,1KAD A,2KA1 A,2KA1 B,2KA2 A,2KA2 B,1KB9 C,1KB9 D,1KB9 E,1KB9 H,1KB9 I,1KBY L,1KBY M,1KBY H,2KB7 P,2KBV A,3KBC A,3KBC B,3KBC C,1KCH A,1KCH B,1KCH C,1KCH D,1KCH E,3KCU A,3KCU B,3KCU C,3KCU D,3KCU E,3KCV A,3KCV B,3KCV C,3KCV

D,3KCV E,2KDC A,2KDC B,2KDC C,3KDP C,3KDP D,3KDP H,1KF6 C,1KF6 D,1KF6 O,1KF6 P,1KFY C,1KFY D,1KFY O,1KFY P,4KFM A,4KFM C,4KFM F,4KFM J,1KG8 A,1KG8 B,1KG8 C,1KG9 A,1KG9 B,1KG9 C,1KGB A,1KGB B,1KGB C,3KG2 A,3KG2 B,3KG2 C,3KG2 D,4KHZ F,4KHZ G,2KI9 A,2KIH A,2KIH B,2KIH C,2KIH D,4KI0 F,4KI0 G,1KJU A,3KJ6 A,4KJP A,4KJP B,4KJQ A,4KJQ B,4KJR A,4KJR C,4KJR D,4KJS A,4KJS C,4KJS D,4KJW A,4KJW B,4KK5 A,4KK5 B,4KK6 A,4KK6 B,4KK8 A,4KK8 B,4KK9 A,4KK9 B,4KKA A,4KKA B,4KKB A,4KKB B,4KKC A,4KKC B,4KKL A,4KKL B,2KLU A,3KLY A,3KLY B,3KLY C,3KLY D,3KLY E,3KLZ A,3KLZ B,3KLZ C,3KLZ D,3KLZ E,1KME A,1KMO A,1KMP A,2KNC A,4KNF A,4KNF B,4KNF C,4KNF D,4KNF E,2KOG A,1KP1 A,1KPK A,1KPK B,1KPL A,1KPL B,1KPN A,1KPW A,1KPX A,2KPE A,2KPE B,2KPF A,3KP9 A,4KPP A,1KQF B,1KQF C,1KQF E,1KQF F,1KQF H,1KQF I,1KQG B,1KQG C,1KQG E,1KQG F,1KQG H,1KQG I,4KR4 A,4KR4 B,4KR4 C,4KR8 A,4KR8 B,4KR8 C,4KRA A,4KRA B,4KRA C,2KS1 A,2KS1 B,2KS9 A,2KSA A,2KSB A,2KSD A,2KSE A,2KSF A,2KSR A,2KSY A,3KSO A,3KSO B,3KSO C,3KSS A,3KSS B,3KSS C,4KSB A,4KSC A,4KSD A,4KT0 A,4KT0 B,4KT0 F,4KT0 J,4KT0 K,4KT0 M,3KVN X,2KWX A,2KWX B,2KWX C,2KWX D,4KX6 C,4KX6 D,4KX6 O,4KX6 P,1KYK A,1KYK B,1KYK C,1KYK D,1KYK E,1KYL A,1KYL B,1KYL C,1KYL D,1KYL E,1KYO C,1KYO D,1KYO E,1KYO H,1KYO I,1KYO N,1KYO O,1KYO P,1KYO S,1KYO T,2KYH A,2KYV A,2KYV B,2KYV C,2KYV D,2KYV E,4KY0 A,4KY0 B,4KY0 C,4KYT A,4KYT B,1KZU A,1KZU B,1KZU D,1KZU E,1KZU G,1KZU H,1KZU C,1KZU F,1KZU I,1KZU J,1KZU K,1KZU L,1KZU M,1KZU N,1KZU P,1KZU Q,1KZU R,1KZU S,3KZI A,3KZI B,3KZI C,3KZI D,3KZI E,3KZI F,3KZI H,3KZI I,3KZI J,3KZI K,3KZI L,3KZI M,3KZI T,3KZI Y,3KZI X,3KZI Z,1L0L C,1L0L D,1L0L E,1L0L G,1L0L J,1L0L K,1L0M A,1L0N C,1L0N D,1L0N E,1L0N G,1L0N J,1L0N K,1L0V C,1L0V D,1L0V O,1L0V P,2L0J A,2L0J B,2L0J C,2L0J D,2L0L A,2L0M A,2L0N A,2L0O A,3L1L A,2L2T A,2L2T B,2L34 A,2L34 B,2L35 A,2L35 B,4L35 A,4L35 B,4L35 C,1L6T A,2L6W A,2L6X A,4L6R A,4L6V A,4L6V B,4L6V F,4L6V L,4L6V M,4L6V K,1L7V A,1L7V B,3L70 C,3L70 D,3L70 E,3L70 G,3L70 J,3L70 P,3L70 Q,3L70 R,3L70 T,3L70 W,3L71 C,3L71 D,3L71 E,3L71 G,3L71 J,3L71 P,3L71 Q,3L71 R,3L71 T,3L71 W,3L72 C,3L72 D,3L72 E,3L72 G,3L72 J,3L72 P,3L72 Q,3L72 R,3L72 T,3L72 W,3L73 C,3L73 D,3L73 E,3L73 G,3L73 J,3L73 P,3L73 Q,3L73 R,3L73 T,3L73 W,3L74 C,3L74 D,3L74 E,3L74 G,3L74 J,3L74 P,3L74 Q,3L74 R,3L74 T,3L74 W,3L75 C,3L75 D,3L75 E,3L75 G,3L75 J,3L75 P,3L75 Q,3L75 R,3L75 T,3L75 W,2L8S A,1L9B L,1L9B M,1L9B H,1L9H A,1L9J L,1L9J M,1L9J H,2L91 A,2L9U A,2LAT A,1LBN A,2LBG A,4LBE C,4LBE D,4LBE E,4LBE F,2LCK A,2LCX A,2LCX B,4LCU C,4LCU D,4LCU E,4LCU F,4LCZ A,4LCZ B,4LCZ C,1LDA A,1LDA B,1LDA C,1LDA D,1LDF A,1LDF B,1LDF C,1LDF D,1LDI A,1LDI B,1LDI C,1LDI D,3LDC A,3LDC B,3LDC C,3LDC D,3LDD A,3LDD B,3LDD C,3LDD D,3LDE A,3LDE B,3LDE C,3LDE D,4LDE A,4LDL A,4LDO A,4LDS A,2LEG B,3LEO A,3LEO B,3LEO C,4LEP A,4LEP B,1LGH A,1LGH B,1LGH D,1LGH E,1LGH M,1LGH N,1LGH P,1LGH Q,1LGH R,1LGH S,1LGH T,1LGH U,1LGH V,1LGH W,1LGH Y,1LGH X,2LHF A,2LJ2 A,2LKG A,2LKH A,1LLK A,1LLY A,2LLM A,2LLY A,3LLQ A,4LLH A,4LLH B,4LLH C,2LM2 A,2LME A,2LME B,2LME C,4LMJ E,4LMJ B,4LMJ C,4LMJ A,4LMJ D,4LMK A,4LMK E,4LMK C,4LMK B,4LMK D,4LML A,4LML B,4LML C,4LML D,4LML E,1LN6 A,1LNQ A,1LNQ B,1LNQ C,1LNQ D,2LNL A,3LNM B,3LNM E,3LNM F,3LNM G,2LOH A,2LOH B,2LOM A,2LOO A,2LOP A,2LOQ A,2LOR A,2LOS A,2LOT A,2LOU A,2LOV A,2LOW A,4LOU

A,4LOU B,2LP1 A,2LPF A,4LP8 A,4LP8 B,4LP8 C,4LP8 D,3LRB A,3LRB B,3LRC A,3LRC B,2LS2 A,2LS3 A,2LS4 A,3LSV A,3LSV B,3LSV C,3LSV D,3LSV E,4LSE A,4LSE B,4LSE C,4LSF A,4LSF C,4LSF D,4LSG A,4LSH A,4LSH C,4LSH D,4LSI A,4LSI B,4LSI C,2LTQ A,4LTO A,4LTO B,4LTO C,4LTO D,4LTP A,4LTP B,4LTP C,4LTP D,4LTQ A,4LTQ B,4LTQ C,4LTQ D,4LTR A,4LTR B,4LTR C,4LTR D,3LUT B,3LUT D,3LUT F,3LUT H,1LVI A,3LW5 A,3LW5 B,3LW5 F,3LW5 G,3LW5 H,3LW5 I,3LW5 J,3LW5 K,3LW5 L,3LW5 1,3LW5 2,3LW5 3,3LW5 4,4LWY H,4LWY L,4LWY M,2LX0 A,4LXJ A,2LZ3 A,2LZ3 B,2LZ4 A,2LZ4 B,2LZL A,4LZ6 A,4LZ9 A,1M0K A,1M0K B,1M0K C,1M0L A,1M0L B,1M0L C,1M0M A,1M0M B,1M0M C,2M06 A,2M07 A,2M0B A,2M0Q A,4M1M A,1M2U A,2M20 A,2M20 B,3M2L A,3M2L B,3M2L C,3M2L D,3M2L E,3M2L F,3M2L G,4M2S A,4M2T A,1M3X L,1M3X M,1M3X H,2M3B A,2M3B B,2M3B C,2M3B D,2M3B E,2M3E A,2M3G A,2M3G B,2M3G C,3M3R A,3M3R B,3M3R C,3M3R D,3M3R E,3M3R F,3M3R G,3M4D A,3M4D B,3M4D C,3M4D D,3M4D E,3M4D F,3M4D G,3M4E A,3M4E B,3M4E C,3M4E D,3M4E E,3M4E F,3M4E G,4M48 A,1M56 A,1M56 B,1M56 C,1M56 D,1M57 A,1M57 B,1M57 C,1M57 D,2M59 A,2M59 B,4M58 A,4M5B A,4M5C A,2M67 A,2M6B A,2M6I A,2M6I B,2M6I C,2M6I D,2M6I E,3M6E A,3M6E B,3M6E C,4M64 B,2M7G A,2M7X A,3M71 A,3M71 B,3M71 C,3M72 A,3M72 B,3M72 C,3M73 A,3M73 B,3M73 C,3M74 A,3M74 B,3M74 C,3M75 A,3M75 B,3M75 C,3M76 A,3M76 B,3M76 C,3M77 A,3M77 B,3M77 C,3M78 A,3M78 B,3M78 C,3M7B A,3M7B B,3M7B C,3M7C A,3M7C B,3M7C C,3M7E A,3M7E B,3M7E C,3M7L A,3M7L B,3M7L C,3M8B A,3M8D A,4M8J A,4M8J B,4M8J C,3M9C L,3M9C M,3M9C N,3M9C R,3M9I A,3M9S L,3M9S M,3M9S N,3M9S R,3M9S H,1MAL A,1MAL B,1MAL C,2MAF A,2MAW A,3MBV A,3MBV B,3MBV C,4MBS A,2MC7 A,4MD1 A,4MD1 B,4MD2 A,4MD2 B,2MET A,2MET B,2MET C,2MEU A,2MEU B,4MEE A,4MES A,2MFR A,1MGY A,2MGY A,1MHS A,1MHS B,2MHL A,4MHW A,2MIC A,2MIC B,2MJO A,4MJN A,4MJN B,4MJN C,4MJN D,4MJN E,4MJN F,4MJN G,4MJN H,4MJN I,4MJN J,4MJN K,4MJN L,4MJN M,4MJN N,2MK9 A,2MKA A,2MKA B,2MKV A,3MK7 A,3MK7 B,3MK7 C,3MK7 U,3MKT A,3MKU A,2MLH A,4MLB A,1MM4 A,1MM5 A,1MMH 1,1MMH 2,1MMH 3,1MMH 4,1MMH 5,1MMH 6,1MMH 7,2MM8 A,2MMU A,4MM4 A,4MM4 B,4MM5 A,4MM6 A,4MM7 A,4MM8 A,4MM8 B,4MM9 A,4MMA A,4MMB A,4MMC A,4MMD A,4MMD B,4MME A,4MME B,4MMF A,4MMF B,2MN6 B,2MN7 A,2MNH A,4MND A,4MND B,1MOT A,2MOF A,2MOM A,2MOM B,2MOM C,2MOZ A,1MPF A,1MPF B,1MPF C,1MPM A,1MPM B,1MPM C,1MPN A,1MPN B,1MPN C,1MPO A,1MPO B,1MPO C,1MPQ A,1MPQ B,1MPQ C,1MPR A,1MPR B,1MPR C,1MPS L,1MPS M,1MPS H,2MPN A,2MPN B,2MPR A,2MPR B,2MPR C,3MP7 A,3MP7 B,3MPN A,3MPN B,3MPQ A,3MPQ B,4MQS A,4MQT A,4MQX A,4MQX B,3MRA A,4MRN A,4MRN B,4MRP A,4MRP B,4MRR A,4MRR B,4MRS A,4MRS B,4MRV A,4MRV B,1MSR A,1MSR B,4MSW C,4MSW D,4MSW E,4MSW F,4MT0 A,4MT0 B,4MT0 C,4MT1 A,4MT1 B,4MT1 C,4MT4 A,4MT4 B,4MT4 C,4MUU A,2MV6 A,2MXB A,4MYC A,4MYC B,4MYH A,4MYH B,2N02 A,2N2L A,2N2M A,3N23 A,3N23 B,3N23 G,1N3M A,1N3M C,4N4D A,4N4R A,4N4W A,4N4Y A,4N4Y B,4N4Y C,2N5S A,3N5K A,2N6L A,2N6P A,4N6H A,1N7L A,4N74 A,4N75 A,4N7K H,4N7K L,4N7K M,4N7L H,4N7L L,4N7L M,4N7W A,4N7W B,4N7X A,3N8G A,2NA6 A,2NA6 B,2NA6 C,2NA7 A,2NA7 B,2NA7 C,3NAL A,3NAM A,3NAN A,4NAB A,3NCY A,3NCY B,4NC3 A,1ND8 A,3ND0 A,3ND0 B,1NE0 A,1NEK C,1NEK D,1NEK G,1NEK H,1NEK K,1NEK L,1NEN

C,1NEN D,1NEN G,1NEN H,1NEN K,1NEN L,3NE2 A,3NE2 B,3NE2 C,3NE2 D,3NE5 A,3NE5 H,3NE5 I,4NEF A,4NEF B,4NEF C,4NEF D,3NFF A,4NH2 A,4NH2 B,4NH2 C,4NH2 D,4NH2 E,4NH2 F,3NJT A,4NJN A,4NJP A,1NKZ A,1NKZ B,1NKZ C,1NKZ D,1NKZ E,1NKZ F,1NKZ G,1NKZ H,1NKZ I,1NKZ J,1NKZ K,1NKZ L,1NKZ M,1NKZ N,1NKZ P,1NKZ Q,1NKZ R,1NKZ S,3NK5 A,3NKA A,3NKA C,3NKA D,3NKA E,3NKC A,3NKC C,3NKC D,3NKC E,2NLJ C,2NLJ D,2NLJ E,2NLJ F,2NMR A,2NMR B,2NMR C,3NMO A,2NOP A,2NOP B,2NOP C,2NOW A,2NOW B,2NOW C,3NOC A,3NOC B,3NOC C,3NOG A,3NOG B,3NOG C,2NPC A,2NPC B,2NPC C,2NPD A,2NPD B,2NPD C,2NPE A,2NPE B,2NPE C,2NPG A,2NPG B,2NPG C,2NPJ A,2NPJ B,2NPJ C,2NPK A,2NPK B,2NPK C,4NPP A,4NPP B,4NPP C,4NPP D,4NPP E,1NQE A,1NQF A,1NQG A,1NQH A,2NQ2 A,2NQ2 B,2NR1 A,2NR9 A,2NRF A,2NRF D,2NRF C,2NRG A,2NS1 A,2NS1 C,2NS1 E,3NS0 A,3NS0 B,3NS0 C,3NSB A,3NSB B,3NSB C,3NSG A,3NSG B,3NSG C,1NTK C,1NTK D,1NTK E,1NTK G,1NTK J,1NTK K,1NTM C,1NTM D,1NTM E,1NTM G,1NTM J,1NTM K,1NTZ C,1NTZ D,1NTZ E,1NTZ G,1NTZ J,1NTZ K,2NTU A,2NTU B,2NTU C,2NTW A,2NTW B,2NTW C,4NTA A,4NTA B,4NTA C,4NTB A,4NTB B,4NTB C,4NTF A,4NTF B,4NTF C,4NTJ A,4NTW A,4NTW D,4NTW G,4NTX A,4NTX D,4NTX G,4NTY A,4NTY D,4NTY G,1NU1 C,1NU1 D,1NU1 E,1NU1 G,1NU1 J,1NU1 K,2NUU A,2NUU B,2NUU C,4NV2 A,4NV5 A,4NV6 A,1NW0 A,1NW0 B,2NWL A,2NWL B,2NWL C,2NWW A,2NWW B,2NWW C,2NWX A,2NWX B,2NWX C,3NY8 A,3NY9 A,3NYA A,3NYM A,4NYK A,4NYK B,4NYK C,1O0A A,1O0A B,1O0A C,2O01 A,2O01 B,2O01 F,2O01 G,2O01 H,2O01 I,2O01 J,2O01 L,2O01 1,2O01 2,2O01 3,2O01 4,3O0E A,3O0R B,3O0R C,2O4V A,2O4V B,2O4V C,3O44 A,3O44 B,3O44 C,3O44 D,3O44 E,3O44 F,3O44 G,1O5W A,2O5P A,4O6M A,4O6M B,4O6N A,4O6N B,4O6Y A,4O6Y B,2O7L A,3O7P A,3O7Q A,4O79 A,4O79 B,4O7G A,4O7G B,2O9D A,2O9E A,2O9E B,2O9E C,2O9E D,2O9F A,2O9G A,2O9G B,2O9G C,2O9G D,2O9J A,4O93 A,4O93 B,4O93 C,4O93 D,4O9P C,4O9P D,4O9P A,4O9P B,4O9R A,4O9T A,4O9T B,4O9T C,4O9T D,4O9U A,4O9U B,4O9U C,4O9U D,2OA0 A,2OAR A,2OAR B,2OAR C,2OAR D,2OAR E,2OAU A,2OAU B,2OAU C,2OAU D,2OAU E,2OAU F,2OAU G,3OAX A,3OAX C,4OAA A,3OB6 A,3OB6 B,1OCC A,1OCC B,1OCC C,1OCC D,1OCC G,1OCC I,1OCC J,1OCC K,1OCC L,1OCC M,1OCC N,1OCC O,1OCC P,1OCC Q,1OCC T,1OCC V,1OCC W,1OCC X,1OCC Y,1OCC Z,1OCO A,1OCO B,1OCO C,1OCO D,1OCO G,1OCO I,1OCO J,1OCO K,1OCO L,1OCO M,1OCO N,1OCO O,1OCO P,1OCO Q,1OCO T,1OCO V,1OCO W,1OCO X,1OCO Y,1OCO Z,1OCR A,1OCR B,1OCR C,1OCR D,1OCR G,1OCR I,1OCR J,1OCR K,1OCR L,1OCR M,1OCR N,1OCR O,1OCR P,1OCR Q,1OCR T,1OCR V,1OCR W,1OCR X,1OCR Y,1OCR Z,1OCZ A,1OCZ B,1OCZ C,1OCZ D,1OCZ G,1OCZ I,1OCZ J,1OCZ K,1OCZ L,1OCZ M,1OCZ N,1OCZ O,1OCZ P,1OCZ Q,1OCZ T,1OCZ V,1OCZ W,1OCZ X,1OCZ Y,1OCZ Z,2OCC A,2OCC B,2OCC C,2OCC D,2OCC G,2OCC I,2OCC J,2OCC K,2OCC L,2OCC M,2OCC N,2OCC O,2OCC P,2OCC Q,2OCC T,2OCC V,2OCC W,2OCC X,2OCC Y,2OCC Z,2ODJ A,3ODJ A,3ODU A,3ODU B,4OD4 A,4OD5 A,1OEA A,1OED A,1OED B,1OED C,1OED D,1OED E,3OE0 A,3OE0 B,3OE6 A,3OE6 B,3OE8 B,3OE8 C,3OE9 A,3OE9 B,1OGV H,1OGV L,1OGV M,3OGC C,3OGC D,3OGC E,3OGC F,4OGQ A,4OGQ B,4OGQ C,4OGQ D,4OGQ E,4OGQ F,4OGQ G,4OGQ H,4OGQ I,4OGQ J,4OGQ K,4OGQ L,4OGQ M,4OGQ N,4OGQ O,4OGQ P,1OH2 P,1OH2 Q,1OH2 R,3OHN A,1OJ9 A,1OJA A,1OJC A,4OJ2 X,4OJ2 A,4OJ2 B,4OJ2

C,1OKC A,2OMF A,2OMF B,2OMF C,3OM3 A,3OM3 B,3OMA A,3OMA B,3OMI A,3OMI B,3OMN A,3OMN B,2ONJ A,2ONJ B,2ONK C,2ONK D,1OPF A,1OPF B,1OPF C,1OPN A,1ORM A,1ORQ C,1ORQ D,1ORQ E,1ORQ F,1ORS C,3OR6 C,3OR6 D,3OR6 E,3OR6 F,3OR7 C,3OR7 D,3OR7 E,3OR7 F,3ORG A,4OR2 A,4OR2 B,1OSM A,1OSM B,1OSM C,1OTS A,1OTS B,1OTT A,1OTT B,1OTU A,1OTU B,3OUF A,3OUF C,3OUF D,3OUF E,3OUS A,3OUS B,3OUS C,3OUS D,1OV0 A,1OV1 A,4OV0 A,4OV0 B,4OV0 C,4OXS A,4OXS B,4OXS E,4OXS F,1OY6 A,1OY6 B,1OY6 C,1OY8 A,1OY8 B,1OY8 C,1OY9 A,1OY9 B,1OY9 C,1OYD A,1OYD B,1OYD C,1OYE A,1OYE B,1OYE C,4OYE A,4OYE B,4OYE C,4OYF A,4OYF B,4OYF C,4OYG A,4OYG B,4OYG C,1OZ5 A,1OZC A,3P03 A,3P03 B,3P03 C,3P0G A,4P00 A,4P00 B,4P02 A,4P02 B,4P19 A,4P19 B,4P19 C,4P1A A,4P1A B,4P1A C,4P2Z A,4P2Z B,4P2Z E,4P2Z F,4P30 A,4P30 B,4P30 E,4P30 F,4P3J A,4P3J B,4P3J C,1P49 A,1P4T A,1P4T B,1P4T C,3P4P C,3P4P D,3P4P O,3P4P P,3P4Q C,3P4Q D,3P4Q O,3P4Q P,3P4R C,3P4R D,3P4R O,3P4R P,3P4S C,3P4S D,3P4S O,3P4S P,3P4W A,3P4W B,3P4W C,3P4W D,3P4W E,3P50 A,3P50 B,3P50 C,3P50 D,3P50 E,3P5N A,4P6H A,4P6H B,4P6H C,4P6J A,4P6J B,4P6K A,4P6K B,4P6L A,4P6L B,4P6V B,4P6V C,4P6V D,4P6V E,4P6V F,1P7B A,1P7B B,1P7B C,1P7B D,2P7T C,2P7T D,2P7T E,2P7T F,4P79 A,1P84 C,1P84 D,1P84 E,1P84 H,1P84 I,1P84 N,1P84 P,1P84 Q,1P84 T,1P84 U,1P8H A,1P8H B,1P8H C,1P8I A,1P8I B,1P8I C,1P8U A,1P8U B,1P8U C,4P9O A,4P9O B,4P9O E,4P9O F,4P9P A,4P9P B,4P9P E,4P9P F,4PA3 A,4PA3 B,4PA3 E,4PA3 F,4PA4 A,4PA4 B,4PA4 E,4PA4 F,4PA6 A,4PA6 B,4PA6 E,4PA6 F,4PA7 A,4PA7 B,4PA7 E,4PA7 F,4PA9 A,4PA9 B,4PA9 E,4PA9 F,1PB2 A,1PB4 C,1PB4 D,3PBL A,4PB1 A,4PB1 B,4PB1 C,4PB2 A,4PB2 B,4PB2 C,4PBU A,4PBU B,4PBU C,4PBU D,4PBU E,4PBU F,4PBU H,4PBU I,4PBU J,4PBU K,4PBU L,4PBU M,4PBU T,4PBU Y,4PBU X,4PBU Z,4PBU A,4PBU B,4PBU C,4PBU D,4PBU E,4PBU F,4PBU H,4PBU I,4PBU J,4PBU K,4PBU L,4PBU M,4PBU T,4PBU Y,4PBU X,4PBU Z,1PCR L,1PCR M,1PCR H,3PCQ A,3PCQ B,3PCQ F,3PCQ I,3PCQ J,3PCQ K,3PCQ L,3PCQ M,3PCQ X,3PCV A,3PCV B,3PCV C,3PDS A,4PD4 C,4PD4 D,4PD4 E,4PD4 H,4PD4 I,4PD5 A,4PD5 B,4PD5 C,4PD6 A,4PD6 B,4PD6 C,4PD7 A,4PD7 B,4PD7 C,4PD8 A,4PD8 B,4PD8 C,4PD9 A,4PD9 B,4PD9 C,4PDA A,4PDA B,4PDA C,4PDL A,4PDL C,4PDL D,4PDL E,4PDM A,4PDM C,4PDM D,4PDM E,4PDR B,4PDR F,4PDR G,4PDR H,4PDV A,4PDV C,4PDV D,4PDV E,2PED A,4PE5 A,4PE5 B,4PE5 C,4PE5 D,3PF1 A,3PGR A,3PGS A,3PGU A,4PGR A,4PGS A,4PGU A,4PGV A,4PGW A,1PHO A,1PHO B,1PHO C,4PHU A,4PHZ D,4PHZ H,4PHZ K,4PHZ N,4PHZ A,4PHZ B,4PHZ E,4PHZ I,4PHZ F,4PHZ J,4PHZ C,4PHZ G,3PIK A,3PIK B,3PIK C,4PI0 D,4PI0 H,4PI0 F,4PI0 K,4PI0 E,4PI0 N,4PI0 A,4PI0 I,4PI0 C,4PI0 G,4PI0 J,4PI0 B,4PI2 D,4PI2 H,4PI2 F,4PI2 E,4PI2 N,4PI2 A,4PI2 I,4PI2 G,4PI2 J,4PI2 B,4PI2 K,4PI2 C,4PIR A,4PIR B,3PJS K,3PJS L,3PJS M,3PJS N,3PJZ A,3PJZ B,4PJ0 A,4PJ0 B,4PJ0 C,4PJ0 D,4PJ0 E,4PJ0 F,4PJ0 H,4PJ0 I,4PJ0 J,4PJ0 K,4PJ0 L,4PJ0 M,4PJ0 T,4PJ0 Y,4PJ0 X,4PJ0 Z,4PJ0 A,4PJ0 B,4PJ0 C,4PJ0 D,4PJ0 E,4PJ0 F,4PJ0 H,4PJ0 I,4PJ0 J,4PJ0 K,4PJ0 L,4PJ0 M,4PJ0 T,4PJ0 X,4PJ0 R,4PJ0 R,4PJ0 Y,4PJ0 Z,1PLN A,1PLN B,1PLN C,1PLN D,1PLN E,3PL9 A,4PL0 A,4PL0 B,1PNZ A,2PNO A,2PNO B,2PNO C,1PO0 A,1PO3 A,2POR A,2POR B,2POR C,3PO7 A,3POQ A,3POQ B,3POQ C,3POR A,3POR B,3POR C,3POU A,3POU B,3POU C,3POX A,3POX B,3POX C,4POP A,4POV A,1PP9 C,1PP9 D,1PP9 E,1PP9 G,1PP9 J,1PP9 P,1PP9 Q,1PP9 R,1PP9 T,1PP9 W,1PPJ C,1PPJ

D,1PPJ E,1PPJ G,1PPJ P,1PPJ Q,1PPJ R,1PPJ T,1PPJ W,2PPS A,2PPS B,2PPS L,2PPS K,2PPS F,3PQR A,1PRC L,1PRC M,1PRC H,1PRN A,1PRN B,1PRN C,2PRC L,2PRC M,2PRC H,2PRN A,2PRN B,2PRN C,3PRC L,3PRC M,3PRC H,3PRN A,3PRN B,3PRN C,4PR7 A,5PRC L,5PRC M,5PRC H,5PRN A,5PRN B,5PRN C,6PRC L,6PRC M,6PRC H,6PRN A,6PRN B,6PRN C,7PRC L,7PRC M,7PRC H,7PRN A,7PRN B,7PRN C,8PRN A,8PRN B,8PRN C,1PSL A,1PSL B,1PSL C,1PSL D,1PSL E,1PSS L,1PSS M,1PSS H,1PST L,1PST M,1PST H,3PUV F,3PUV G,3PUW F,3PUW G,3PUX F,3PUX G,3PUY F,3PUY G,3PUZ F,3PUZ G,1PV6 A,1PV7 A,3PV0 F,3PV0 G,4PV1 A,4PV1 B,4PV1 C,4PV1 D,4PV1 E,4PV1 F,4PV1 G,4PV1 H,4PV1 I,4PV1 J,4PV1 K,4PV1 L,4PV1 M,4PV1 N,4PV1 O,4PV1 P,1PW4 A,3PWH A,1PXR A,1PXR D,1PXR C,1PXS A,1PXS D,1PXS C,3PXO A,4PX7 A,4PXF A,4PXF C,4PXK A,4PXK B,4PXK C,4PXZ A,1PY6 A,1PY6 D,1PY6 C,1PYH A,1PYH B,1PYH C,1PYH D,1PYH E,1PYH F,1PYH G,1PYH H,1PYH I,1PYH J,1PYH K,1PYH L,1PYH M,1PYH N,1PYH O,1PYH P,1PYH Q,1PYH R,1PYH S,1PYH T,1PYH U,1PYH V,1PYH W,1PYH X,1PYH Y,1PYH Z,1PYH 1,1PYH 2,1PYH 3,1PYH 4,1PYH 5,1PYH 6,1PYH 7,1PYH 8,4PY0 A,4PYP A,1Q16 C,1Q16 F,3Q17 A,3Q17 B,4Q2E A,4Q2E B,4Q2G A,4Q2G B,4Q35 A,4Q4A A,4Q4A B,4Q4H A,4Q4H B,4Q4J A,4Q4J B,1Q5I A,1Q5I D,1Q5I C,1Q5J A,1Q5J D,1Q5J C,2Q67 A,2Q67 B,2Q67 C,2Q67 D,2Q68 A,2Q68 B,2Q68 C,2Q68 D,2Q69 A,2Q69 B,2Q69 C,2Q69 D,2Q6A A,2Q6A B,2Q6A C,2Q6A D,2Q6H A,4Q65 A,2Q72 A,2Q72 B,2Q7M A,2Q7M B,2Q7M C,2Q7R A,2Q7R B,2Q7R C,3Q7K A,3Q7K B,3Q7K C,3Q7K D,3Q7K E,4Q7C A,4Q7C B,1Q90 A,1Q90 B,1Q90 D,1Q90 R,1Q90 G,1Q90 L,1Q90 M,1Q90 N,1Q90 E,1Q90 F,1Q90 I,1Q90 J,1Q90 K,1Q90 O,1Q90 P,1Q90 Q,1Q9F A,1Q9G A,4Q9H A,4Q9I A,4Q9J A,4Q9K A,4Q9L A,3QAK A,3QAP A,2QB4 A,2QB4 B,3QBG A,3QBG B,3QBG D,3QBI A,3QBI B,3QBI D,3QBK A,3QBK B,3QBK D,3QBL A,3QBL B,3QBL D,1QCR C,1QCR D,1QCR E,1QCR G,1QCR J,1QCR K,1QD5 A,1QD6 C,1QD6 D,3QDC A,4QD6 A,2QEI A,2QEI B,3QE7 A,3QE7 B,4QE7 A,4QE7 B,4QE7 C,4QE7 D,4QE9 A,4QE9 B,4QE9 C,4QE9 D,1QFF A,1QFG A,2QFI A,3QF4 A,3QF4 B,1QHJ A,1QHJ B,1QHJ C,4QH1 A,4QH1 B,4QH1 C,4QH1 D,4QH1 E,4QH4 A,4QH4 B,4QH4 C,4QH4 D,4QH4 E,4QH5 A,4QH5 B,4QH5 C,4QH5 D,4QH5 E,2QI9 A,2QI9 B,4QI1 A,4QI1 B,4QI1 C,4QID A,4QIM A,4QIN A,4QIN B,4QIQ A,1QJ8 A,1QJ9 A,1QJP A,1QJQ A,2QJK A,2QJK B,2QJK C,2QJK D,2QJK E,2QJK F,2QJP A,2QJP B,2QJP C,2QJP D,2QJP E,2QJP F,2QJU A,2QJY A,2QJY B,2QJY C,2QJY D,2QJY E,2QJY F,3QJQ A,3QJQ B,3QJQ C,3QJR A,3QJR B,3QJR C,3QJS A,3QJS B,3QJS C,3QJT A,3QJT B,3QJT C,3QJU A,3QJU B,3QJU C,3QJV A,3QJV B,3QJV C,1QKC A,1QKO A,1QKO B,1QKO C,1QKP A,2QKS A,2QKS C,2QKS D,2QKS E,4QKX A,4QKY A,1QLB C,1QLB F,1QLE A,1QLE B,1QLE C,1QLE D,3QLB A,3QLB B,4QL0 A,1QM8 A,1QM8 B,1QM8 C,3QNQ A,3QNQ B,4QNC A,4QNC B,4QND A,4QND B,4QNZ A,1QO1 K,1QO1 L,1QO1 M,1QO1 N,1QO1 O,1QO1 P,1QO1 Q,1QO1 R,1QO1 S,1QO1 T,1QOV L,1QOV M,1QOV H,2QOM A,4QO0 A,4QO0 B,4QO2 A,2QPD A,2QPD B,2QPD C,2QPE A,2QPE B,2QPE C,3QQ2 A,3QRA A,3QRC A,4QRY A,4QRY B,4QRY C,3QS4 A,3QS4 B,3QS5 A,3QS5 B,3QS6 A,3QS6 B,2QTK A,2QTO A,2QTO B,2QTO C,2QTO D,2QTS A,2QTS B,2QTS C,4QTN A,4QTN B,4QTN C,4QUV A,4R0C A,4R0C B,4R1I A,4R1I B,1R2C L,1R2C M,1R2C H,1R2N A,1R3I C,1R3I D,1R3I G,1R3I K,1R3J C,1R3J F,1R3J I,1R3J L,1R3K C,1R3K F,1R3K I,1R3K L,1R3L C,1R3L F,1R3L I,1R3L L,2R4L B,2R4N A,2R4O A,2R4P A,2R4R A,2R4S A,4R50 A,4R50 C,4R50 D,4R50 E,2R6G

F,2R6G G,3R65 A,3R65 B,3R65 C,3R65 D,4R6Z A,4R6Z C,4R6Z D,4R6Z E,4R7C A,4R7C B,4R7C E,4R7C F,1R84 A,2R88 A,2R89 A,2R8A A,4R8C A,4R8C C,4R8C D,4R8C E,2R9H A,2R9H B,2R9R B,2R9R C,2R9R D,2R9R E,4R9U A,4R9U B,4RAI A,4RAI C,4RAI D,4RAI E,3RBH A,1RC2 B,2RCR L,2RCR M,2RCR H,3RCE A,4RCR L,4RCR M,4RCR H,2RDD A,2RDD B,2RDD C,2RDD D,2RDD E,2RDD F,4RDQ A,4RDQ B,4RDQ C,4RDQ D,4RDQ E,4RDR A,4RDT A,3REY A,4RES A,4RES B,4RES G,4RET A,4RET B,4RET G,4RET E,3RFM A,3RFU A,3RFZ B,4RFS S,4RFS T,1RG5 L,1RG5 M,1RG5 H,1RGN L,1RGN M,1RGN H,3RGB A,3RGB B,3RGB C,3RGB E,3RGB I,3RGB J,3RGB F,3RGB K,3RGB G,3RGM A,3RGN A,1RH5 A,1RH5 B,1RH5 C,1RHZ A,1RHZ B,1RHZ C,2RH1 A,3RHW A,3RHW B,3RHW C,3RHW D,3RHW E,4RHB A,3RI5 A,3RI5 B,3RI5 C,3RI5 D,3RI5 E,3RIA A,3RIA B,3RIA C,3RIA D,3RIA E,3RIF A,3RIF B,3RIF C,3RIF D,3RIF E,4RI2 A,4RI2 B,4RI3 A,4RI3 B,4RJW A,4RJW B,4RJW C,4RJX A,4RJX B,4RJX C,1RKL A,3RKO L,3RKO M,3RKO N,3RKO K,3RKO A,3RKO J,3RLB A,3RLF F,3RLF G,4RL8 A,4RL9 A,4RLB A,4RLB B,4RLC A,2RMZ A,2RN0 A,4RNG A,4RNG C,4RO2 A,4RO2 B,4RO2 E,4RO2 F,4RP8 A,4RP8 C,4RP9 A,4RP9 B,1RQK L,1RQK M,1RQK H,3RQU A,3RQU B,3RQU C,3RQU D,3RQU E,3RQW A,3RQW B,3RQW C,3RQW D,3RQW E,4RUE A,4RUE B,4RUF A,4RUF B,1RVJ L,1RVJ M,1RVJ H,3RVY A,3RVY B,3RVY C,3RVY D,3RVZ A,3RVZ B,3RVZ C,3RVZ D,4RVW A,4RVY A,4RVY A,4RVY B,4RVY B,4RVY C,4RVY C,4RVY D,4RVY D,4RVY E,4RVY E,4RVY F,4RVY F,4RVY H,4RVY H,4RVY I,4RVY I,4RVY J,4RVY J,4RVY K,4RVY K,4RVY L,4RVY L,4RVY M,4RVY M,4RVY T,4RVY T,4RVY X,4RVY X,4RVY Y,4RVY Y,4RVY Z,4RVY Z,1RWT B,1RWT F,1RWT G,3RW0 A,3RW0 B,3RW0 C,3RW0 D,4RWA A,4RWA B,4RWD A,4RWD B,1RY5 L,1RY5 M,1RY5 H,4RY2 A,4RY2 B,4RYI A,4RYI B,4RYJ A,4RYJ B,4RYM A,4RYN A,4RYO A,4RYQ A,4RYR A,1RZH L,1RZH M,1RZH H,1RZZ L,1RZZ M,1RZZ H,3RZE A,1S00 L,1S00 M,1S00 H,3S0X A,3S0X B,4S0F A,4S0F B,4S0V A,1S2Q A,1S2Y A,1S33 A,1S33 B,1S33 C,1S33 D,1S3B A,1S3E A,3S33 A,3S33 B,3S33 C,3S38 A,3S38 B,3S38 C,3S39 A,3S39 B,3S39 C,3S3A A,3S3A B,3S3A C,3S3B A,3S3B B,3S3B C,3S3C A,3S3C B,3S3C C,3S3D A,3S3D B,3S3D C,3S3W A,3S3W B,3S3W C,3S3X A,3S3X B,3S3X C,1S51 A,1S51 C,1S51 E,1S52 A,1S53 A,1S53 C,1S53 E,1S54 A,1S54 C,1S54 E,1S5H C,1S5H F,1S5H I,1S5H L,1S5L A,1S5L B,1S5L C,1S5L D,1S5L E,1S5L F,1S5L H,1S5L I,1S5L J,1S5L K,1S5L L,1S5L M,1S5L T,1S5L X,1S5L N,1S5L Z,1S5L A,1S5L B,1S5L C,1S5L D,1S5L E,1S5L F,1S5L H,1S5L I,1S5L J,1S5L K,1S5L L,1S5L M,1S5L T,1S5L X,1S5L N,1S5L Z,1S6E A,1S8J A,1S8L A,3S8F A,3S8F B,3S8F C,3S8G A,3S8G B,3S8G C,3SFD C,3SFD D,3SFE C,3SFE D,1SIW C,1SIW F,1SKH A,3SLJ A,3SLO A,3SLT A,3SN6 R,1SOR A,1SOR B,1SOR C,1SOR D,3SPC A,3SPC B,3SPC C,3SPC D,3SPG A,3SPG B,3SPG C,3SPG D,3SPH A,3SPH B,3SPH C,3SPH D,3SPI A,3SPI B,3SPI C,3SPI D,3SPJ A,3SPJ B,3SPJ C,3SPJ D,1SQB C,1SQB D,1SQB E,1SQB G,1SQB J,1SQB K,1SQP C,1SQP D,1SQP E,1SQP G,1SQP J,1SQP K,1SQQ C,1SQQ D,1SQQ E,1SQQ G,1SQQ J,1SQQ K,1SQV C,1SQV D,1SQV E,1SQV G,1SQV J,1SQV K,1SQX C,1SQX E,1SQX D,1SQX G,1SQX K,1SQX J,1SR1 A,3STL C,3STL D,3STL E,3STL F,3STZ C,3STZ D,3STZ E,3STZ F,1SU4 A,1SUK A,3SY7 A,3SY9 A,3SYA A,3SYA B,3SYA C,3SYA D,3SYB A,3SYC A,3SYC B,3SYC C,3SYC D,3SYO A,3SYO B,3SYO C,3SYO D,3SYP A,3SYP B,3SYP C,3SYP D,3SYQ A,3SYQ B,3SYQ C,3SYQ D,3SYS A,3SZD B,3SZV A,3T0S A,1T16 A,1T1L A,1T1L B,3T1C A,3T1C C,3T1C

D,3T1C E,3T20 A,3T24 A,3T2M A,3T2M C,3T2M D,3T2M E,3T45 A,3T45 B,3T45 C,3T4D B,3T4D F,3T4D G,3T4D H,3T4Z A,3T4Z C,3T4Z D,3T4Z E,1T5S A,1T5T A,3T51 A,3T51 F,3T51 I,3T53 A,3T53 F,3T53 I,3T56 A,3T56 D,3T56 G,3T6D H,3T6D L,3T6D M,3T6E H,3T6E L,3T6E M,1T78 A,1T9T A,1T9T B,1T9T C,1T9U A,1T9U B,1T9U C,1T9V A,1T9V B,1T9V C,1T9W A,1T9W B,1T9W C,1T9X A,1T9X B,1T9X C,1T9Y A,1T9Y B,1T9Y C,3T9N A,3T9N B,3T9N C,3T9N D,3T9N E,3T9N F,3T9N G,3TCU A,3TCU C,3TCU D,3TCU E,3TDO A,3TDO B,3TDO C,3TDO D,3TDO E,3TDP A,3TDP B,3TDP C,3TDP D,3TDP E,3TDR A,3TDR B,3TDR C,3TDR D,3TDR E,3TDS E,3TDS A,3TDS B,3TDS C,3TDS D,3TDX A,3TDX B,3TDX C,3TDX D,3TDX E,3TE0 A,3TE0 B,3TE0 C,3TE0 D,3TE0 E,3TE1 A,3TE1 B,3TE1 C,3TE1 D,3TE1 E,3TE2 A,3TE2 B,3TE2 C,3TE2 D,3TE2 E,3TET A,3TET C,3TET D,3TET E,1THQ A,3TIJ A,3TIJ B,3TIJ C,4TKQ A,4TKR A,1TLW A,1TLY A,1TLZ A,3TLM A,3TLS A,3TLS B,3TLS C,3TLS D,3TLS E,3TLT A,3TLT B,3TLT C,3TLT D,3TLT E,3TLU A,3TLU B,3TLU C,3TLU D,3TLU E,3TLV A,3TLV B,3TLV C,3TLV D,3TLV E,3TLW A,3TLW B,3TLW C,3TLW D,3TLW E,4TL3 A,4TL3 B,4TLL A,4TLL B,4TLL C,4TLL D,4TLM A,4TLM B,4TLM C,4TLM D,1TN0 A,1TN0 D,1TN0 C,1TN5 A,1TN5 D,1TN5 C,4TNH A,4TNH B,4TNH C,4TNH D,4TNH E,4TNH F,4TNH H,4TNH I,4TNH J,4TNH K,4TNH L,4TNH M,4TNH T,4TNH Y,4TNH X,4TNH Y,4TNH Z,4TNH A,4TNH B,4TNH C,4TNH D,4TNH E,4TNH F,4TNH H,4TNH I,4TNH J,4TNH K,4TNH L,4TNH M,4TNH T,4TNH G,4TNH X,4TNH G,4TNH Z,4TNI A,4TNI B,4TNI C,4TNI D,4TNI E,4TNI F,4TNI H,4TNI I,4TNI J,4TNI K,4TNI L,4TNI M,4TNI T,4TNI Y,4TNI X,4TNI Y,4TNI Z,4TNI A,4TNI B,4TNI C,4TNI D,4TNI E,4TNI F,4TNI H,4TNI I,4TNI J,4TNI K,4TNI L,4TNI M,4TNI T,4TNI G,4TNI X,4TNI G,4TNI Z,4TNJ A,4TNJ B,4TNJ C,4TNJ D,4TNJ E,4TNJ F,4TNJ H,4TNJ I,4TNJ J,4TNJ K,4TNJ L,4TNJ M,4TNJ T,4TNJ Y,4TNJ X,4TNJ Y,4TNJ Z,4TNJ A,4TNJ B,4TNJ C,4TNJ D,4TNJ E,4TNJ F,4TNJ H,4TNJ I,4TNJ J,4TNJ K,4TNJ L,4TNJ M,4TNJ T,4TNJ G,4TNJ X,4TNJ G,4TNJ Z,4TNK A,4TNK B,4TNK C,4TNK D,4TNK E,4TNK F,4TNK H,4TNK I,4TNK J,4TNK K,4TNK L,4TNK M,4TNK T,4TNK Y,4TNK X,4TNK Y,4TNK Z,4TNK A,4TNK B,4TNK C,4TNK D,4TNK E,4TNK F,4TNK H,4TNK I,4TNK J,4TNK K,4TNK L,4TNK M,4TNK T,4TNK G,4TNK X,4TNK G,4TNK Z,4TNV A,4TNV B,4TNV C,4TNV D,4TNV E,4TNW A,4TNW B,4TNW C,4TNW D,4TNW E,4TPG A,4TPG B,4TPH A,4TPH B,4TPJ A,4TPJ B,1TQQ A,1TQQ B,1TQQ C,4TQ3 A,4TQ4 A,4TQ5 A,4TQ6 A,4TQQ H,4TQQ L,4TQQ M,4TQU M,4TQU N,4TQV A,4TQV B,4TSY A,4TSY B,3TT1 A,3TT1 B,3TT3 A,3TT3 B,3TU0 A,3TU0 B,3TUI A,3TUI B,3TUJ A,3TUJ B,3TUZ A,3TUZ B,4TWD A,4TWD B,4TWD C,4TWD D,4TWD E,4TWF A,4TWF B,4TWF C,4TWF D,4TWF E,4TWH A,4TWH B,4TWH C,4TWH D,4TWH E,4TWK A,4TWK B,1TXH A,1TXH B,1TXH C,1TXH D,1TXH E,1TXH F,3TX3 A,3TXT A,3TXT B,3TXT C,1U19 A,4U14 A,4U15 A,4U16 A,4U1W A,4U1W B,4U1W C,4U1W D,4U1X A,4U1X B,4U1X C,4U1X D,4U1Y A,4U1Y B,4U1Y C,4U1Y D,3U2F K,3U2F L,3U2F M,3U2F N,3U2F O,3U2F A,3U2F B,3U2F C,3U2F D,3U2F E,3U2Y K,3U2Y L,3U2Y M,3U2Y N,3U2Y O,3U2Y A,3U2Y B,3U2Y C,3U2Y D,3U2Y E,4U2P A,4U2P B,4U2P D,4U2P C,4U2Q A,4U2Q B,4U2Q C,4U2Q D,3U32 K,3U32 L,3U32 M,3U32 N,3U32 O,3U32 A,3U32 B,3U32 C,3U32 D,3U32 E,4U3F C,4U3F D,4U3F E,4U3F G,4U3F J,4U3F P,4U3F Q,4U3F R,4U3F T,4U3F W,4U4F A,4U4F B,4U4F C,4U4F D,4U4G A,4U4G B,4U4G C,4U4G D,4U4T A,4U4V A,4U4W A,4U4W B,1U5N A,4U5B A,4U5B B,4U5B C,4U5B D,4U5C A,4U5C B,4U5C C,4U5C D,4U5D

A,4U5D B,4U5D C,4U5D D,4U5E A,4U5E B,4U5E C,4U5E D,4U5F A,4U5F B,4U5F C,4U5F D,1U77 A,1U77 B,1U77 C,1U7C A,1U7C B,1U7C C,1U7G A,1U7G B,1U7G C,4U8V A,4U8V B,4U8Y A,4U8Y B,4U95 A,4U95 B,4U96 A,4U96 B,4U96 C,4U9L A,4U9L B,4U9N A,4U9N B,1UAZ A,1UAZ C,1UAZ F,3UBB A,3UBB B,3UBB C,4UB6 A,4UB6 B,4UB6 C,4UB6 D,4UB6 E,4UB6 F,4UB6 H,4UB6 I,4UB6 J,4UB6 K,4UB6 L,4UB6 M,4UB6 T,4UB6 Y,4UB6 X,4UB6 Z,4UB6 R,4UB6 A,4UB6 B,4UB6 C,4UB6 D,4UB6 E,4UB6 F,4UB6 H,4UB6 I,4UB6 J,4UB6 K,4UB6 L,4UB6 M,4UB6 T,4UB6 Y,4UB6 X,4UB6 Z,4UB8 A,4UB8 B,4UB8 C,4UB8 D,4UB8 E,4UB8 F,4UB8 H,4UB8 I,4UB8 J,4UB8 K,4UB8 L,4UB8 M,4UB8 T,4UB8 Y,4UB8 X,4UB8 Z,4UB8 R,4UB8 A,4UB8 B,4UB8 C,4UB8 D,4UB8 E,4UB8 F,4UB8 H,4UB8 I,4UB8 J,4UB8 K,4UB8 L,4UB8 M,4UB8 T,4UB8 Y,4UB8 X,4UB8 Z,1UCQ A,1UCQ B,1UCQ C,4UC1 A,4UC1 B,4UC2 A,4UC2 B,4UC3 A,4UC3 B,3UD0 K,3UD0 L,3UD0 M,3UD0 N,3UD0 O,3UD0 A,3UD0 B,3UD0 C,3UD0 D,3UD0 E,3UDC A,3UDC B,3UDC C,3UDC D,3UDC E,3UDC F,3UDC G,1UFD A,1UFD B,1UFD C,1UFD D,3UG9 A,3UG9 B,4UG2 A,4UHR A,4UIS A,4UIS B,4UIS C,4UIS D,1UJW A,3UKM A,3UKM B,1UMX H,1UMX L,1UMX M,3UM7 A,3UM7 B,4UMV A,4UMW A,3UON A,1UPE A,3UPG A,3UPG B,3UPG C,4UP6 A,4UP6 B,4UP6 C,3UQ4 A,3UQ4 B,3UQ4 C,3UQ4 D,3UQ4 E,3UQ5 A,3UQ5 B,3UQ5 C,3UQ5 D,3UQ5 E,3UQ7 A,3UQ7 B,3UQ7 C,3UQ7 D,3UQ7 E,4UQQ A,4UQQ B,4UQQ C,4UQQ D,3USG A,3USG B,3USI A,3USJ A,3USK A,3USK B,3USL A,3USL B,3USM A,3USM B,3USO A,3USO B,3USP A,3USP B,4US3 A,4US4 A,3UTV A,3UTW A,3UTX A,3UTY A,1UUN A,1UUN B,1UUN C,1UUN D,1UUN E,1UUN F,1UUN G,1UUN H,2UUH A,2UUH B,2UUH C,2UUI A,2UUI B,2UUI C,3UU2 A,3UU2 B,3UU2 C,3UU3 A,3UU3 B,3UU3 C,3UU3 D,3UU3 E,3UU4 A,3UU4 B,3UU4 C,3UU4 D,3UU4 E,3UU5 A,3UU5 B,3UU5 C,3UU5 D,3UU5 E,3UU6 A,3UU6 B,3UU6 C,3UU6 D,3UU6 E,3UU8 A,3UU8 B,3UU8 C,3UU8 D,3UU8 E,3UUB A,3UUB B,3UUB C,3UUB D,3UUB E,4UU0 A,4UU1 A,4UUJ C,4UUJ D,4UUJ E,4UUJ F,1UV3 A,4UV3 A,4UV3 B,4UV3 C,4UV3 D,4UV3 E,4UV3 F,4UV3 G,4UV3 H,4UV3 I,4UVM A,2UWS H,2UWS L,2UWS M,2UWT H,2UWT L,2UWT M,2UWU H,2UWU L,2UWU M,2UWV H,2UWV L,2UWV M,2UWW H,2UWW L,2UWW M,4UWA A,4UWA B,4UWA C,4UWA D,4UWE A,4UWE B,4UWE C,4UWE D,1UXF A,2UX3 H,2UX3 L,2UX3 M,2UX4 H,2UX4 L,2UX4 M,2UX5 H,2UX5 L,2UX5 M,2UXJ H,2UXJ L,2UXJ M,2UXK H,2UXK L,2UXK M,2UXL H,2UXL L,2UXL M,2UXM H,2UXM L,2UXM M,3UX4 A,3UX4 B,3UX4 C,4UX1 A,4UX1 B,4UX2 A,4UX2 B,4UXW A,4UXW B,4UXW C,4UXX A,4UXX B,4UXX C,4UXZ A,4UXZ B,4UXZ C,1UYN X,1UYO X,4UYO A,4UYO B,4UYO C,3UZA A,3UZC A,4V1F A,4V1F B,4V1F C,4V1F D,4V1F E,4V1F F,4V1F G,4V1F H,4V1F I,4V1G A,4V1G B,4V1G C,4V1G D,4V1G E,4V1G F,4V1G G,4V1G H,4V1G I,4V1H A,4V1H B,4V1H C,4V1H D,4V1H E,4V1H F,4V1H G,4V1H H,4V1H I,3V2W A,3V2Y A,1V36 A,1V36 B,1V36 C,1V36 D,1V36 E,1V36 F,1V36 G,3V3C A,3V3C B,3V3C C,3V3C D,3V3C E,3V3C F,3V3C G,3V3C H,3V3C I,3V3C J,3V3C K,3V3C L,3V3C M,3V3C N,3V3Y H,3V3Y L,3V3Y M,3V3Z H,3V3Z L,3V3Z M,4V3G A,4V3H A,1V54 A,1V54 B,1V54 C,1V54 D,1V54 G,1V54 I,1V54 J,1V54 K,1V54 L,1V54 M,1V54 N,1V54 O,1V54 P,1V54 Q,1V54 T,1V54 V,1V54 W,1V54 X,1V54 Y,1V54 Z,1V55 A,1V55 B,1V55 C,1V55 D,1V55 G,1V55 I,1V55 J,1V55 K,1V55 L,1V55 M,1V55 N,1V55 O,1V55 P,1V55 Q,1V55 T,1V55 V,1V55 W,1V55 X,1V55 Y,1V55 Z,2V50 A,2V50 B,2V50 C,2V5Z A,3V5S A,3V5U A,2V60 A,2V61 A,2V8N A,3V89 A,3V8F A,3V8F B,3V8F C,3V8G A,3V8G B,3V8G C,3V8X A,1VCR A,2VDD

A,2VDD B,2VDD C,2VDE A,2VDE B,2VDE C,2VDF A,1VF5 A,1VF5 B,1VF5 C,1VF5 D,1VF5 E,1VF5 F,1VF5 G,1VF5 H,1VF5 N,1VF5 O,1VF5 P,1VF5 Q,1VF5 R,1VF5 S,1VF5 T,1VF5 U,1VFP A,1VGO A,1VGO C,1VGO F,3VG9 A,3VGA A,3VHZ A,3VHZ B,3VHZ C,3VI0 A,3VI0 B,3VI0 C,1VJM A,1VJM B,1VJM C,2VL0 A,2VL0 B,2VL0 C,2VL0 D,2VL0 E,3VMQ A,3VMR A,3VMS A,3VMT A,2VOY B,2VOY C,2VOY D,2VOY E,2VOY G,2VOY H,2VOY K,2VOY L,3VOU A,3VOU B,3VOU C,3VOU D,2VPW C,2VPW G,2VPX C,2VPX G,2VPY C,2VPY G,2VPZ C,2VPZ G,2VQI A,1VRN H,1VRN L,1VRN M,1VRY A,2VR0 C,2VR0 F,2VRL A,2VRM A,3VR8 C,3VR8 D,3VR9 C,3VR9 D,3VRA C,3VRA D,3VRB C,3VRB D,2VT4 A,2VV5 A,2VV5 B,2VV5 C,2VV5 D,2VV5 E,2VV5 F,2VV5 G,3VVK A,3VVK B,3VVK C,3VVN A,3VVO A,3VVP A,3VVR A,3VVS A,3VW7 A,3VY8 X,3VY8 A,3VY8 B,3VY9 X,3VY9 A,3VY9 B,1VZ1 A,3VZT X,3VZT A,3VZT B,3VZU X,3VZU A,3VZU B,3VZW X,3VZW A,3VZW B,2W0F C,2W0F D,2W0F E,2W0F F,2W16 A,2W1B A,2W1B B,2W1B C,2W1P A,2W1P B,2W1P C,2W1P D,2W2E A,2W2E B,2W2E C,2W2E D,3W4T A,1W5C A,1W5C B,1W5C C,1W5C D,1W5C E,1W5C F,1W5C G,1W5C H,1W5C I,1W5C J,1W5C K,1W5C L,1W5C X,1W5C Y,2W5J A,2W5J B,2W5J C,2W5J D,2W5J E,2W5J F,2W5J G,2W5J H,2W5J I,2W5J J,2W5J K,2W5J L,2W5J M,2W5J V,3W5A A,3W5A C,3W5B A,3W5C A,3W5D A,2W6T A,2W6U A,4W6V A,2W75 A,2W76 A,2W77 A,2W78 A,3W9H A,3W9H B,3W9H C,3W9I A,3W9I B,3W9I C,3W9J A,3W9J B,3W9J C,3W9T A,3W9T C,3W9T G,3W9T B,3W9T F,3W9T E,3W9T D,1WAZ A,3WAJ A,3WAK A,4WAB A,4WAB B,4WAV A,3WBN A,2WDQ C,2WDQ D,2WDQ G,2WDQ H,2WDQ K,2WDQ L,2WDR C,2WDR D,2WDR G,2WDR H,2WDV C,2WDV D,2WDV G,2WDV H,2WDV K,2WDV L,3WDO A,4WD7 A,4WD7 B,4WD7 C,4WD7 D,4WD7 E,4WD8 A,4WD8 B,4WD8 C,4WD8 D,4WD8 E,3WFB B,3WFB C,3WFC B,3WFC C,3WFD B,3WFD C,3WFE B,3WFE C,4WFE A,4WFE B,4WFF A,4WFF B,4WFG A,4WFG B,4WFH A,4WFH B,2WGM A,2WGM B,2WGM C,2WGM D,2WGM E,2WGM F,2WGM G,2WGM H,2WGM I,2WGM J,2WGM K,3WG7 A,3WG7 B,3WG7 C,3WG7 D,3WG7 G,3WG7 I,3WG7 J,3WG7 K,3WG7 L,3WG7 M,3WG7 N,3WG7 O,3WG7 P,3WG7 Q,3WG7 T,3WG7 V,3WG7 W,3WG7 X,3WG7 Y,3WG7 Z,3WGU C,3WGU D,3WGU E,3WGV C,3WGV D,3WGV E,4WGV A,4WGV C,4WGW A,4WGW C,2WIE A,2WIE B,2WIE C,2WIE D,2WIE E,2WIE F,2WIE G,2WIE H,2WIE I,2WIE J,2WIE K,2WIE L,2WIE M,2WIE N,2WIE P,2WIT A,2WIT B,2WIT C,3WI4 A,3WI4 B,3WI4 C,3WI5 A,3WI5 B,3WI5 C,4WIB A,4WIB B,4WIS A,4WIS B,4WIT A,4WIT B,2WJK A,2WJK B,2WJK C,2WJL A,2WJL B,2WJL C,2WJM H,2WJM L,2WJM M,2WJN H,2WJN M,2WJN L,2WJQ A,2WJR A,2WLH A,2WLH B,2WLH C,2WLH D,2WLI A,2WLI B,2WLI D,2WLI E,2WLJ A,2WLJ B,2WLJ C,2WLJ D,2WLK A,2WLK B,2WLK C,2WLK D,2WLL A,2WLL B,2WLL C,2WLL D,2WLM A,2WLM B,2WLM C,2WLM D,2WLN A,2WLN B,2WLN C,2WLN D,2WLO A,2WLO B,3WME A,3WME B,3WMF A,3WMF B,3WMG A,3WMM L,3WMM M,3WMM H,3WMM A,3WMM B,3WMM D,3WMM E,3WMM F,3WMM G,3WMM I,3WMM J,3WMM K,3WMM N,3WMM O,3WMM P,3WMM R,3WMM S,3WMM T,3WMM U,3WMM V,3WMM W,3WMM X,3WMM Y,3WMM Z,3WMM 1,3WMM 2,3WMM 3,3WMM 4,3WMM 5,3WMM 6,3WMM 7,3WMM 8,3WMM 9,3WMM 0,4WMZ A,3WO6 A,3WO7 A,4WO1 A,4WOL A,4WOL B,1WP1 A,1WP1 C,1WP1 D,1WPG A,2WP9 C,2WP9 D,2WP9 G,2WP9 H,2WP9 K,2WP9 L,2WPD J,2WPD K,2WPD L,2WPD M,2WPD N,2WPD O,2WPD P,2WPD Q,2WPD R,2WPD S,2WQY C,2WQY D,3WQJ A,3WQJ B,3WQJ C,1WRG

A,2WS3 C,2WS3 D,2WS3 G,2WS3 H,2WS3 K,2WS3 L,2WSC A,2WSC B,2WSC F,2WSC G,2WSC H,2WSC I,2WSC J,2WSC K,2WSC L,2WSC R,2WSC 1,2WSC 2,2WSC 3,2WSC 4,2WSE A,2WSE B,2WSE F,2WSE G,2WSE H,2WSE I,2WSE J,2WSE K,2WSE L,2WSE R,2WSE 1,2WSE 2,2WSE 3,2WSE 4,2WSF A,2WSF B,2WSF F,2WSF G,2WSF H,2WSF I,2WSF J,2WSF K,2WSF L,2WSF R,2WSF 1,2WSF 2,2WSF 3,2WSF 4,2WSW A,2WSW B,2WSW C,2WSX A,2WSX B,2WSX C,1WU0 A,2WU2 C,2WU2 D,2WU2 G,2WU2 H,2WU2 K,2WU2 L,2WU5 C,2WU5 D,2WU5 G,2WU5 H,2WU5 K,2WU5 L,3WU2 A,3WU2 B,3WU2 C,3WU2 D,3WU2 E,3WU2 F,3WU2 H,3WU2 I,3WU2 J,3WU2 K,3WU2 L,3WU2 M,3WU2 T,3WU2 Y,3WU2 X,3WU2 Z,3WU2 A,3WU2 B,3WU2 C,3WU2 D,3WU2 E,3WU2 F,3WU2 H,3WU2 I,3WU2 J,3WU2 K,3WU2 L,3WU2 M,3WU2 T,3WU2 Y,3WU2 X,3WU2 Z,2WVP A,3WVF A,4WW3 A,2WX5 H,2WX5 L,2WX5 M,3WXV A,3WXW A,1X0I 1,1X0K 1,1X0S A,1X0S B,1X0S C,4X1H A,2X27 X,2X2V A,2X2V B,2X2V C,2X2V D,2X2V E,2X2V F,2X2V G,2X2V H,2X2V I,2X2V J,2X2V K,2X2V L,2X2V M,3X29 A,3X2Q A,3X2Q B,3X2Q C,3X2Q D,3X2Q G,3X2Q I,3X2Q J,3X2Q K,3X2Q L,3X2Q M,3X2Q N,3X2Q O,3X2Q P,3X2Q Q,3X2Q T,3X2Q V,3X2Q W,3X2Q X,3X2Q Y,3X2Q Z,3X2R D,3X2R A,3X2R B,3X2R C,3X2R E,3X2R F,3X2R G,3X2R H,3X2R I,4X2S A,4X2S B,4X2S C,3X3B A,3X3B B,3X3C A,3X3C B,4X31 A,4X31 B,4X31 C,4X32 A,4X32 B,4X32 C,2X4M A,2X55 A,2X56 A,2X5U H,2X5U L,2X5U M,2X5V H,2X5V M,2X5V L,4X5M B,4X5M C,4X5N A,4X5N B,4X5T A,4X5T B,4X5T C,4X5T D,4X5T E,2X6A A,2X6A B,2X6A C,2X6A D,2X6B A,2X6B B,2X6B C,2X6B D,2X6C A,2X6C B,2X6C C,2X6C D,2X72 A,2X72 C,2X79 A,2X9K A,4XDJ A,4XDJ B,4XDL A,4XDL B,2XE1 A,2XE1 B,2XE1 C,2XE2 A,2XE2 B,2XE2 C,2XE3 A,2XE3 C,2XE3 D,2XE5 A,2XE5 B,2XE5 C,4XEE A,4XES A,1XFH A,1XFH B,1XFH C,2XFN A,2XFO A,2XFP A,2XFQ A,2XFU A,2XG6 A,2XG6 B,2XG6 C,1XIO A,1XIO B,4XIG M,4XIG N,1XJI A,1XKH A,1XKW A,4XK8 A,4XK8 B,4XK8 F,4XK8 G,4XK8 H,4XK8 I,4XK8 J,4XK8 K,4XK8 L,4XK8 1,4XK8 2,4XK8 3,4XK8 4,1XL4 A,1XL4 B,1XL4 C,1XL4 D,1XL6 A,1XL6 B,1XL6 C,1XL6 D,1XME A,1XME B,1XME C,2XMN A,2XMN B,2XMN C,1XNU A,1XNU B,1XNU C,1XNU D,1XNU E,2XND J,2XND K,2XND L,2XND M,2XND N,2XND O,2XND P,2XND Q,4XNI A,4XNJ A,4XNK A,4XNL A,4XNU A,4XNV A,4XNW A,4XNX A,2XOK K,2XOK L,2XOK M,2XOK N,2XOK O,2XOK P,2XOK Q,2XOK R,2XOK S,2XOK T,2XOV A,2XOW A,4XOU A,1XP5 A,4XP1 A,4XP4 A,4XP5 A,4XP6 A,4XP9 C,4XPA A,4XPB A,4XPF A,4XPG A,4XPH A,4XPT A,1XQE A,1XQE B,1XQE C,1XQF A,1XQF B,1XQF C,2XQ2 A,2XQ2 B,2XQ3 A,2XQ3 B,2XQ3 C,2XQ3 D,2XQ3 E,2XQ4 A,2XQ4 B,2XQ4 C,2XQ4 D,2XQ4 E,2XQ5 A,2XQ5 B,2XQ5 C,2XQ5 D,2XQ5 E,2XQ6 A,2XQ6 B,2XQ6 C,2XQ6 D,2XQ6 E,2XQ7 A,2XQ7 B,2XQ7 C,2XQ7 D,2XQ7 E,2XQ8 A,2XQ8 B,2XQ8 C,2XQ8 D,2XQ8 E,2XQ9 A,2XQ9 B,2XQ9 C,2XQ9 D,2XQ9 E,2XQA A,2XQA B,2XQA C,2XQA D,2XQA E,2XQS A,2XQS B,2XQS C,2XQS D,2XQS E,2XQS F,2XQS G,2XQS H,2XQS I,2XQS J,2XQS K,2XQS L,2XQS M,2XQS N,2XQS P,2XQT A,2XQT B,2XQT C,2XQT D,2XQT E,2XQT F,2XQT G,2XQT H,2XQT I,2XQT J,2XQT K,2XQT L,2XQT M,2XQT N,2XQT P,2XQU A,2XQU B,2XQU C,2XQU D,2XQU E,2XQU F,2XQU G,2XQU H,2XQU I,2XQU J,2XQU K,2XQU L,2XQU M,2XQU N,2XQU P,1XRD A,2XTU A,2XTV A,4XTL A,4XTN A,4XTN B,4XTO A,4XTO B,2XUT A,4XU4 A,4XU4 B,4XU4 C,4XU5 A,4XU5 B,4XU5 C,4XU6 A,4XU6 B,4XU6 C,4XXJ A,4XXJ B,4XXJ C,2XZB A,2XZB B,2Y00 A,2Y01 A,2Y02 A,2Y03 A,2Y04 A,2Y0H A,2Y0K A,2Y0L A,4Y1K A,4Y1K B,4Y1K C,1Y2L

A,2Y2X A,4Y28 A,4Y28 B,4Y28 I,4Y28 J,4Y28 F,4Y28 G,4Y28 L,4Y28 H,4Y28 K,4Y28 2,4Y28 4,4Y28 1,4Y28 3,1Y36 A,4Y3U A,4Y3U B,4Y3U C,1Y4Z C,1Y4Z F,1Y5D A,1Y5I C,1Y5I F,1Y5L C,1Y5L F,1Y5N C,1Y5N F,2Y5Y A,2Y69 A,2Y69 B,2Y69 C,2Y69 D,2Y69 G,2Y69 I,2Y69 J,2Y69 K,2Y69 L,2Y69 M,2Y69 N,2Y69 O,2Y69 P,2Y69 Q,2Y69 T,2Y69 V,2Y69 W,2Y69 X,2Y69 Y,2Y69 Z,4Y6K A,4Y7J A,4Y7J B,4Y7J C,4Y7J D,4Y7J E,4Y7K A,4Y7K B,4Y7K C,4Y7K D,4Y7K E,1Y8S A,1Y9C A,4YAY A,2YBB C,2YBB D,2YBB E,2YBB G,2YBB J,2YBB K,2YBB L,2YBB M,2YBB N,2YBB O,2YBB R,2YBB T,2YBB U,2YBB V,2YBB W,2YBB X,2YBB C,2YBB D,2YBB E,2YBB G,2YBB J,2YBB K,2YBB M,2YBB N,2YBB O,2YBB P,4YB9 D,4YBQ A,4YBQ B,1YC9 A,1YC9 B,1YC9 C,1YCE A,1YCE B,1YCE C,1YCE D,1YCE E,1YCE F,1YCE G,1YCE H,1YCE I,1YCE J,1YCE K,2YCW A,2YCX A,2YCY A,2YCZ A,4YCL A,4YCM A,4YCN A,4YCR A,4YCR B,4YCR C,2YDO A,2YDV A,1YEW A,1YEW B,1YEW C,1YEW E,1YEW F,1YEW G,1YEW I,1YEW J,1YEW K,2YEV A,2YEV B,2YEV C,4YEU A,4YEU B,4YEU C,4YEU D,4YEU E,1YF6 L,1YF6 M,1YF6 H,2YFY A,1YG1 A,1YG7 A,2YIU A,2YIU B,2YIU C,2YIU D,2YIU E,2YIU F,2YKS A,2YKS B,2YKS C,2YKS D,2YKS E,4YK5 A,4YK5 B,4YK5 C,4YL0 A,4YL0 B,4YL0 C,4YL1 A,4YL1 B,4YL1 C,4YL3 A,4YL3 B,4YL3 C,1YMG A,1YMG B,1YMG C,1YMG D,4YMK A,4YMK D,4YMS D,4YMS C,4YMT C,4YMT B,4YMU D,4YMU C,4YMV D,4YMV C,4YMW D,4YMW C,2YN6 A,2YN6 B,2YN6 C,2YN6 D,2YN6 E,2YN9 A,2YN9 B,2YNK A,1YO9 A,1YO9 B,1YO9 F,1YO9 G,1YO9 H,1YO9 I,1YO9 J,1YO9 K,1YO9 L,1YO9 1,1YO9 2,1YO9 3,1YO9 4,2YOE A,2YOE B,2YOE C,2YOE D,2YOE E,2YPT A,1YQ3 C,1YQ3 D,1YQ4 C,1YQ4 D,1YST L,1YST M,1YST H,2YSU A,4YSX C,4YSX D,4YSY C,4YSY D,4YSZ C,4YSZ D,4YT0 C,4YT0 D,4YTM C,4YTM D,4YTN C,4YTN D,4YTP C,4YTP D,2YVX A,2YVX B,2YXQ A,2YXQ B,2YXQ C,2YXR A,2YXR B,2YXR C,4YZF A,4YZF B,4YZI A,4YZI B,4Z34 A,4Z35 A,4Z36 A,4Z3N A,4Z3P A,2Z55 A,2Z55 C,2Z55 F,2Z5X A,2Z5Y A,1Z65 A,2Z73 A,4Z7F A,4Z7F B,1Z8E A,1Z98 A,1Z98 B,1Z98 C,1Z98 D,1Z9J A,1Z9J B,1Z9J C,1Z9K A,1Z9K B,1Z9K C,4Z90 A,4Z90 B,4Z90 C,4Z90 D,4Z90 E,4Z91 A,4Z91 B,4Z91 C,4Z91 D,4Z91 E,2ZBD A,2ZBE A,2ZBF A,2ZBG A,4ZBM A,4ZBM C,4ZBM D,4ZBM E,1ZC7 A,1ZCD A,1ZCD C,2ZD9 A,2ZD9 B,2ZD9 C,2ZD9 D,3ZDQ A,3ZDQ B,3ZE3 A,3ZE3 B,3ZE3 C,3ZE4 A,3ZE4 B,3ZE4 C,3ZE5 A,3ZE5 B,3ZE5 C,3ZEB A,3ZEV A,2ZFE A,2ZFE B,2ZFE C,2ZFG A,2ZFG B,2ZFG C,2ZIY A,2ZJS Y,2ZJS E,3ZJZ A,3ZJZ B,3ZJZ E,3ZJZ F,3ZK1 A,3ZK1 B,3ZK1 C,3ZK1 D,3ZK1 E,3ZK1 F,3ZK1 G,3ZK1 H,3ZK1 I,3ZK1 J,3ZK1 K,3ZK2 A,3ZK2 B,3ZK2 C,3ZK2 D,3ZK2 E,3ZK2 F,3ZK2 G,3ZK2 H,3ZK2 I,3ZK2 J,3ZK2 K,3ZKR A,3ZKR B,3ZKR C,3ZKR D,3ZKR E,1ZLL A,1ZLL B,1ZLL C,1ZLL D,1ZLL E,2ZLD B,2ZLD E,2ZLD G,2ZLE D,2ZLE N,2ZLE P,3ZMH A,3ZMI A,3ZMJ A,1ZOY C,1ZOY D,3ZO6 A,3ZO6 B,3ZO6 C,3ZO6 D,3ZO6 E,3ZO6 F,3ZO6 H,3ZO6 I,3ZO6 J,3ZO6 K,3ZO6 L,3ZO6 M,3ZOJ A,3ZOJ B,3ZOJ C,3ZOJ D,3ZOT A,4ZOW A,1ZP0 C,1ZP0 D,3ZPQ A,3ZPR A,4ZP0 A,4ZP2 A,2ZQP Y,2ZQP E,1ZRT C,1ZRT D,1ZRT E,1ZRT P,1ZRT Q,1ZRT R,3ZRS A,3ZRS B,3ZRS C,3ZRS D,3ZRY J,3ZRY K,3ZRY L,3ZRY M,3ZRY N,3ZRY O,3ZRY P,3ZRY Q,3ZRY R,3ZRY S,4ZR0 A,4ZR1 A,1ZTI A,2ZT9 A,2ZT9 B,2ZT9 C,2ZT9 D,2ZT9 E,2ZT9 F,2ZT9 G,2ZT9 H,2ZT9 I,2ZT9 J,2ZT9 K,2ZT9 L,2ZT9 M,2ZT9 N,2ZT9 P,2ZT9 Q,2ZUP B,2ZUQ A,3ZUM H,3ZUM L,3ZUM M,3ZUW H,3ZUW L,3ZUW M,3ZUX A,3ZUY A,4ZUD A,1ZV0 B,1ZWI C,1ZWI D,1ZWI E,1ZWI F,2ZW3 A,2ZW3 B,2ZW3 C,2ZW3 D,2ZW3 E,2ZW3 F,4ZW9 A,4ZWB A,4ZWC A,4ZWJ A,2ZXE A,2ZXE B,2ZXE G,2ZXW A,2ZXW B,2ZXW C,2ZXW

D,2ZXW G,2ZXW I,2ZXW J,2ZXW K,2ZXW L,2ZXW M,2ZXW N,2ZXW O,2ZXW P,2ZXW Q,2ZXW T,2ZXW V,2ZXW W,2ZXW X,2ZXW Y,2ZXW Z,1ZYY A,1ZYY B,2ZY9 A,2ZY9 B,4ZYO A,4ZYR A,1ZZA A,2ZZ9 A,2ZZ9 B,2ZZ9 C,2ZZ9 D,2ZZL A,2ZZL B,2ZZL C

The PDB ID is followed by the chain ID used in this study.

II. Supplemental Figures

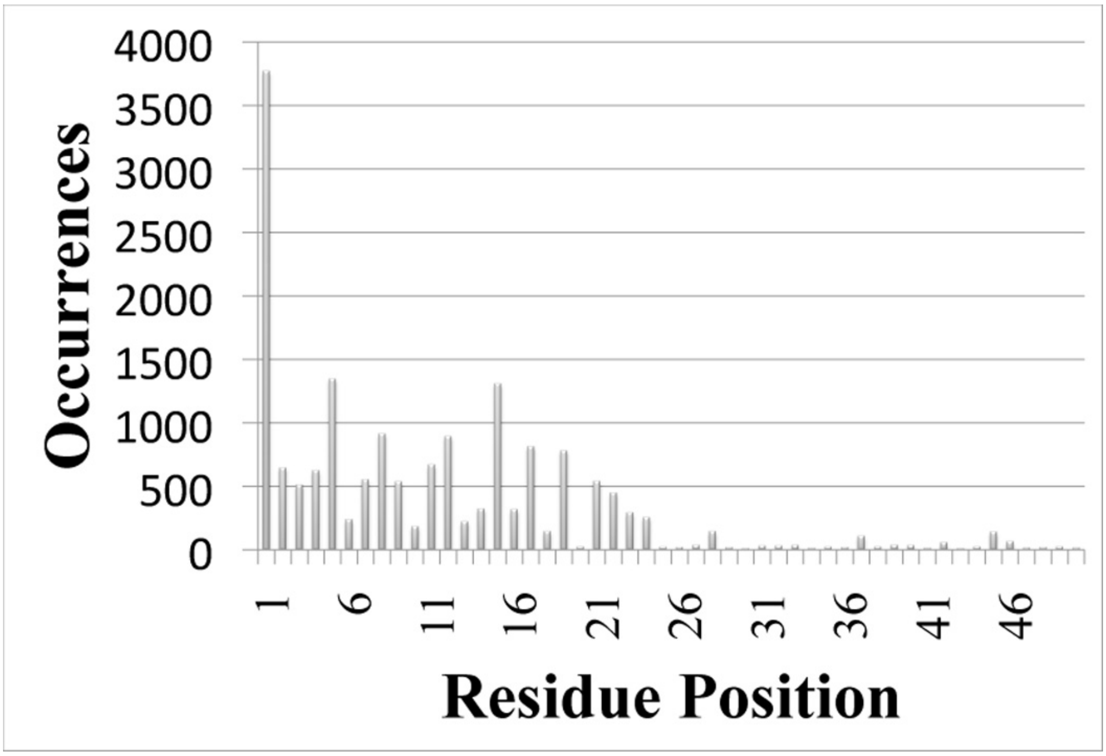

Figure S1. Distribution of identified mutants over HokC sequence. The number of mutants observed (Occurrances, Y axis) at every residue position (X axis) along the HokC sequence are presented as histograms.

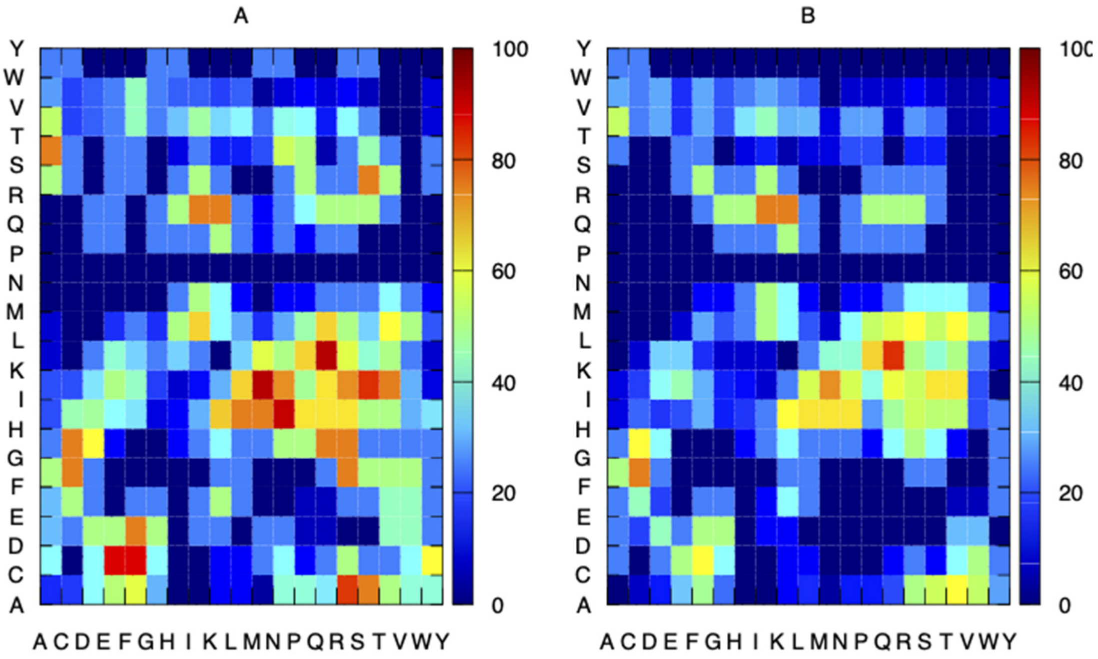

Figure S2. Heat map for HokC substitutions. The frequency of every substitution (Y axis) on any of the original HokC amino acids (X axis) rendering a wild-type (A) and mutant (B) phenotype are represented by a color scale, where the dark blue represent the lowest frequency and dar red the highest. The data for this figure was derived from Table S3A and S3B.

A)

B)

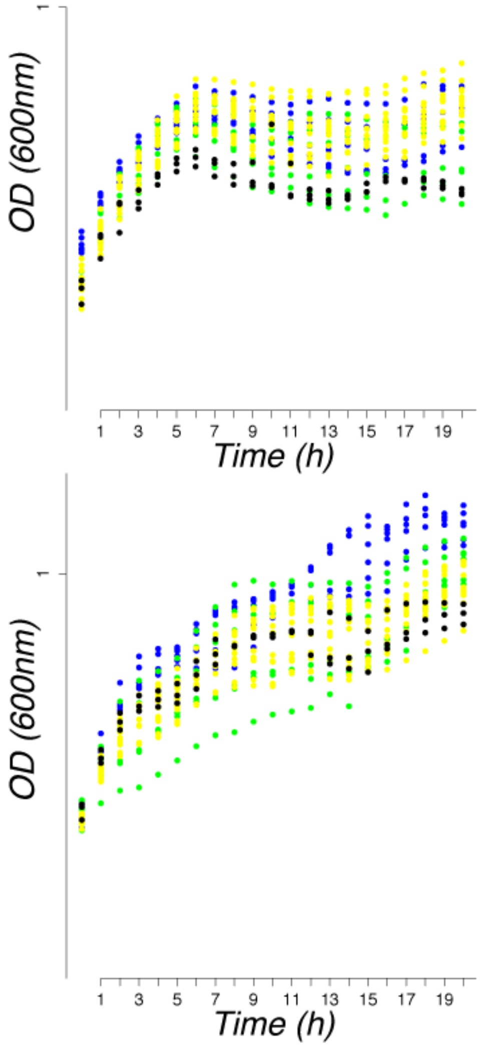

Figure S3. Cell growth of HokC fusions. *E. coli* cells expressing HokC (black circles) or any selected variant were grown to reach 0.4-0.5 of optical density (OD)

measured at 600 nm in rich media and then were exposed to an inducer of HokC-GFP (IPTG) or HokC-phoA (Arabinose) expression. The image presents in colored circles each variant: Met7Trp (blue circles), Ile12Ser (green), Met7Trp-Ile12Ser (yellow). **A)** Optical density measured during 20 h for cells expressing HokC, or any of its variants, fused to GFP. **B)** Optical density measured during 20 h for cells expressing HokC, or any of its variants, fused to phoA. Each panel presents the results of 5 independent experimental results.

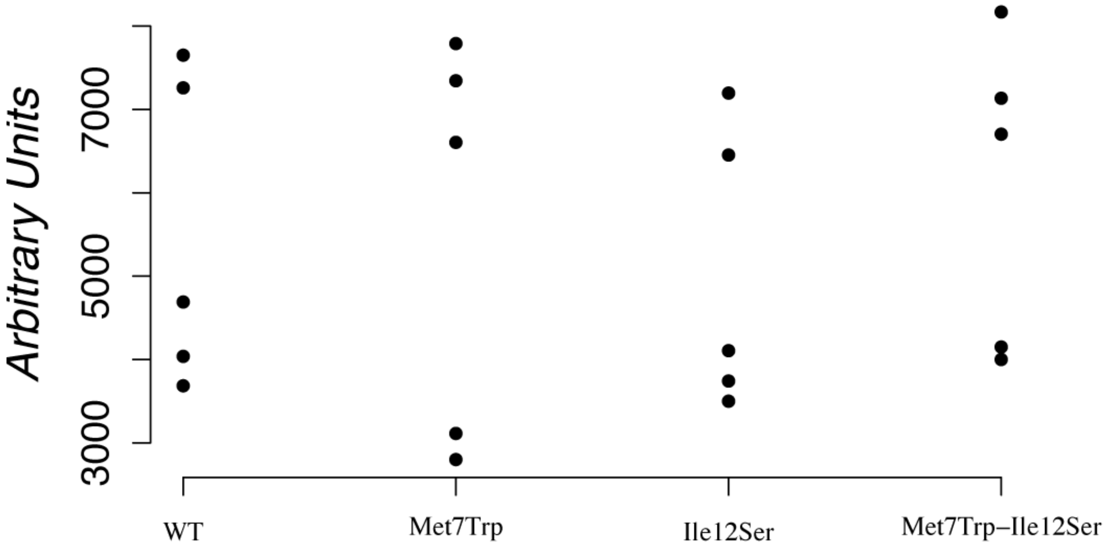

Figure S4. Phosphatase activity measured on cells expressing HokC or variants. Arbitrary units of phosphatse activity measured on cells expressing HokC, HokC:Met7Trp, HokC:Ile12Ser or HokC:Met7Trp-Ile12Ser. Please refer to Methods for the details on how this activity was measured.
